# The Genome of the Generalist Plant Pathogen *Fusarium avenaceum* Is Enriched with Genes Involved in Redox, Signaling and Secondary Metabolism

**DOI:** 10.1371/journal.pone.0112703

**Published:** 2014-11-19

**Authors:** Erik Lysøe, Linda J. Harris, Sean Walkowiak, Rajagopal Subramaniam, Hege H. Divon, Even S. Riiser, Carlos Llorens, Toni Gabaldón, H. Corby Kistler, Wilfried Jonkers, Anna-Karin Kolseth, Kristian F. Nielsen, Ulf Thrane, Rasmus J. N. Frandsen

**Affiliations:** 1 Department of Plant Health and Plant Protection, Bioforsk - Norwegian Institute of Agricultural and Environmental Research, Ås, Norway; 2 Eastern Cereal and Oilseed Research Centre, Agriculture and Agri-Food Canada, Ottawa, Canada; 3 Department of Biology, Carleton University, Ottawa, Canada; 4 Section of Mycology, Norwegian Veterinary Institute, Oslo, Norway; 5 Biotechvana, València, Spain; 6 Bioinformatics and Genomics Programme, Centre for Genomic Regulation, Barcelona, Spain; 7 Universitat Pompeu Fabra, Barcelona, Spain; 8 Institució Catalana de Recerca i Estudis Avançats, Barcelona, Spain; 9 ARS-USDA, Cereal Disease Laboratory, St. Paul, Minnesota, United States of America; 10 Department of Crop Production Ecology, Swedish University of Agricultural Sciences, Uppsala, Sweden; 11 Department of Systems Biology, Technical University of Denmark, Lyngby, Denmark; Seoul National University, Republic Of Korea

## Abstract

*Fusarium avenaceum* is a fungus commonly isolated from soil and associated with a wide range of host plants. We present here three genome sequences of *F. avenaceum,* one isolated from barley in Finland and two from spring and winter wheat in Canada. The sizes of the three genomes range from 41.6–43.1 MB, with 13217–13445 predicted protein-coding genes. Whole-genome analysis showed that the three genomes are highly syntenic, and share>95% gene orthologs. Comparative analysis to other sequenced Fusaria shows that *F. avenaceum* has a very large potential for producing secondary metabolites, with between 75 and 80 key enzymes belonging to the polyketide, non-ribosomal peptide, terpene, alkaloid and indole-diterpene synthase classes. In addition to known metabolites from *F. avenaceum*, fuscofusarin and JM-47 were detected for the first time in this species. Many protein families are expanded in *F. avenaceum*, such as transcription factors, and proteins involved in redox reactions and signal transduction, suggesting evolutionary adaptation to a diverse and cosmopolitan ecology. We found that 20% of all predicted proteins were considered to be secreted, supporting a life in the extracellular space during interaction with plant hosts.

## Introduction

*Fusarium* is a large, ubiquitous genus of ascomycetous fungi that includes many important plant pathogens, as well as saprophytes and endophytes. The genomes of sixteen *Fusarium* spp. have been sequenced during the past decade with a focus on species that either display a narrow host plant range or which have a saprophytic life style. *Fusarium avenaceum* is a cosmopolitan plant pathogen with a wide and diverse host range and is reported to be responsible for disease on>80 genera of plants [1]. It is well-known for causing ear blight and root rot of cereals, blights of plant species within genera as diverse as *Pinus* and *Eustoma* [2], as well as post-harvest storage rot of numerous crops, including potato [3], broccoli [4], apple [5] and rutabaga [6]. *Fusarium avenaceum* has also been described as an endophyte [7], [8] and an opportunistic pathogen of animals [9], [10]. The generalist pathogen nature of *F. avenaceum* is supported by several reports on isolates that lack host specificity. One example of this is the report of *F. avenaceum* isolates from *Eustroma* sp. (aka Lisianthus) being phylogenetically similar to isolates from diverse geographical localities or which have been isolated from other hosts [11].

*Fusarium avenaceum* is often isolated from diseased grains in temperate areas, but an increased prevalence has also been reported in warmer regions throughout the world [12], [13]. The greatest economic impact of *F. avenaceum* is associated with crown rot and head blight of wheat and barley, and the contamination of grains with mycotoxins [12]. Co-occurrence of multiple *Fusarium* species in head blight infections is often observed, and several studies covering the boreal and hemiboreal climate zones in the northern hemisphere have revealed that *F. avenaceum* is often among the dominating species [14]. Previously, *F. avenaceum* has been shown to produce several secondary metabolites, including moniliformin, enniatins, fusarin C, antibiotic Y, 2-amino-14,16-dimethyloctadecan-3-ol (2-AOD-3-ol), chlamydosporol, aurofusarin [12], [15] and recently also fusaristatin A [16].

The genus *Fusarium* includes both broad-host pathogenic species, utilizing a generalist strategy, and narrow-host pathogenic species, which are specialized to a limited number of plant species. The *F. oxysporum* complex is a well-documented example of the specialist strategy, as each *forma specialis* displays a narrow host range. The genetic basis for this host specialization is dictated by a limited number of transferable genes, encoded on dispensable chromosomes [17]. However, the genetic foundation that allows *F. avenaceum* to infect such a wide range of host plant species and cope with such a diverse set of environmental conditions is currently not well understood. In an effort to shed light on the genetic factors that separates generalists from specialists within *Fusarium*, we sequenced the genomes of three different *F. avenaceum* strains isolated from two geographical locations, Finland and Canada, and from three small grain host plants: barley, spring and winter wheat. Comparison with existing *Fusarium* genomes would further explore pathogenic strategies.

## Results and Discussion

### Fusarium avenaceum genome sequences

We have sequenced three *F. avenaceum* genomes, one Finnish isolate from barley (Fa05001) and two Canadian isolates from spring (FaLH03) and winter wheat (FaLH27). Assembly of the 454 pyrosequencing based genomic sequence data from Fa05001 resulted in a total genome size of 41.6 Mb, while assembly of the Illumina HiSeq data for FaLH03 and FaLH27 resulted in genome sizes of 42.7 Mb and 43.1 Mb, respectively. Additional details on the assemblies can be found in (Table 1). Gene calling of the three *F. avenaceum* strains resulted in 13217 (Fa05001, gene naming convention *FAVG1_XXXXX*), 13293 (FaLH03, genes named *FAVG2_XXXXX*) and 13445 (FaLH27, genes named *FAVG3_XXXXX*) unique protein coding gene models. Previous comparative genomics studies of filamentous fungi have identified 69 core genes that are found ubiquitously across all fungal clades [18]. All three gene sets included the 69 core genes, suggesting a good assembly and reliable protein-coding gene prediction. Genome sequence data has been deposited at NCBI GenBank in the Whole Genome Shotgun (WGS) database as accession no. JPYM00000000 (Fa05001), JQGD00000000 (FaLH03) and JQGE00000000 (FaLH27), within BioProject PRJNA253730. The versions described in this paper are JPYM01000000, JQGD01000000, and JQGE01000000.

**Table 1:** Main assembly summary and annotation features of the three *F. avenaceum* genomes.

Strain	Fa05001	FaLH03	FaLH27
Sequencing technology	454	Hiseq	Hiseq
Genome size (Mb)	41.6	42.7	43.2
Sequencing coverage*	21.6x	426.6x	986.2x
Number of contigs	110	180	169
Number of scaffolds	83	104	77
Number of Large Scaffolds (>100 Kb)	40	22	18
Number of Large Scaffolds (>1 Mb)	17	14	11
N50 scaffold length (Mb)	1.43	4.11	4.14
L50 scaffold count	10	5	5
GC content (%)	48%	48%	48%
Number of predicted genes	13217	13293	13445
Average no of genes per Mb	317.7	311.6	311.8
Mean gene length (base pairs)	1554	1557	1552

*Post- removal of mitochondrial genome.

The mitochondrial genome sequence was contained within a single assembled contig for each strain (Fa05001, 49075 bp; FaLH03, 49402 bp; FaLH27, 49396 bp), supporting sufficient coverage and a high quality assembly. Prior to trimming, the FaLH03 and FaLH27 mitochondrial contigs contained 39 and 53 bp, respectively, of sequence duplicated at each end, as expected with the acquisition of a circular sequence. As found in other *Fusarium* mitochondrial genomes [19], [20], the *F. avenaceum* mitochondrial genome sequences contain a low G+C content (about 33%) and encode 26 tRNAs and the ribosomal rRNAs *rnl* and *rns*. In addition, the 14 expected core genes (*cob, cox1, cox2, cox3, nad1, nad2, nad3, nad4, nad4L, nad5, nad6, atp6, atp8, atp9*) involved in oxidative phosphorylation and ATP production are present and in the same order as other *Fusarium* mitochondrial genomes.

### Genome structure in *F. avenaceum*

Electrophoretic karyotyping was performed to resolve the number of chromosomes in Fa05001. Previous karyotyping via fluorescence *in situ* hybridization has suggested that *F. avenaceum* isolated from wheat had 8–10 chromosomes [21]. Our attempt to determine the chromosome number in *F. avenaceum* Fa05001 strain by electrophoretic karyotyping was hampered due to the large size of several of the chromosomes. Southern analysis using a telomeric probe did however result in the detection of four distinct bands ranking from 1 to 5 MB, and several diffuse bands above the detection limit of the method (∼5 Mb) (Figure S1 in File S1). A high order reordering of the scaffolds from the three sequenced genomes resulted in 11 supercontigs ranging from 0.8 Mb to 6.5 Mb in size, likely corresponding to entire chromosomes or chromosome arms (Figure S2 in File S1). The three genomes display a high level of microcolinearity and only a single putative large genome rearrangement was observed in an internal region of Supercontig 1 between Fa5001 and Canadian isolates (Figure S3 in File S1).

**Figure 1:**
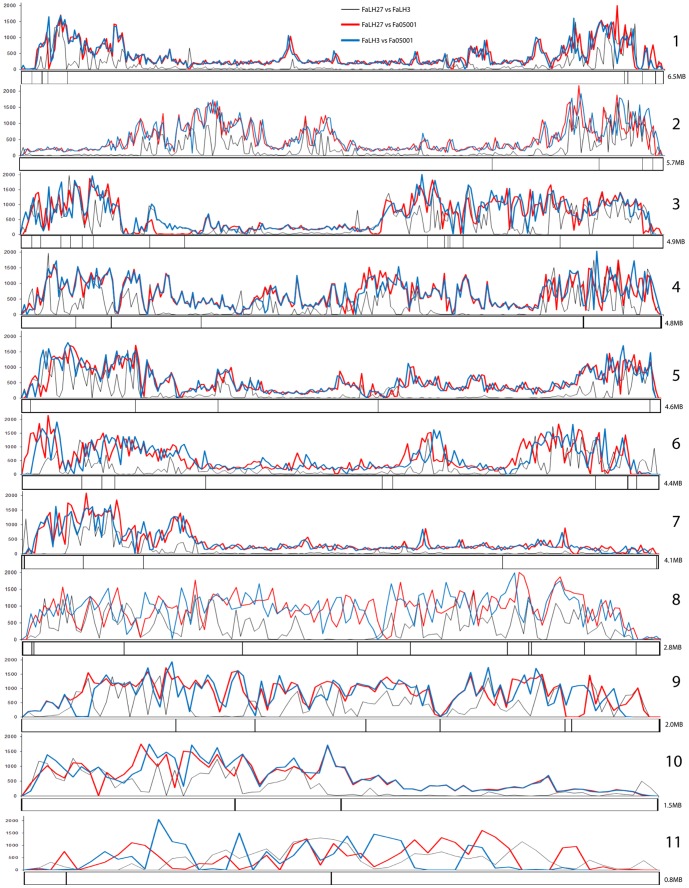
Sliding window map with numbers of SNP's and indels per 20 kb in the three *F. avenaceum* strains Fa05001, FaLH03 and FaLH27 on the 11 supercontigs. Locations of the polyketide synthase and non-ribosomal peptide synthetase genes in the strain FaLH27 are plotted on the supercontigs.

Sequence comparisons between the three genomes revealed a 91–96% nucleotide alignment, with the two Canadian isolates having the fewest unaligned bases. In addition, approximately 1.4–3.2% of the aligned nucleotides exhibited single nucleotide polymorphisms (SNPs), insertions, or deletions between isolates; these were also fewer between the Canadian isolates (Figure 1). These genetic differences were unevenly distributed across the genomes and were largely concentrated at the ends of the supercontigs, while centrally located regions remained relatively conserved. This is similar to what has been previously observed between chromosomes of other *Fusarium* spp. [22]. BLASTn analysis indicated that more than 95% of predicted genes had a significant hit within the two other *F. avenaceum* genomes (Figure S4 in File S1). Together, the results suggest that, despite the large geographical distance between the collection sites, there is a high level of similarity between the three *F. avenaceum* genomes, both in genome structure and gene content. However, some instances of poorly conserved or missing genes were observed in either one or two isolates out of the three (Figure S4 in File S1, File S2, File S3). For example, the three isolates contained some unique polyketide synthases and non-ribosomal peptide synthases. This suggests that there may be some differences in secondary metabolism between the isolates.

### Comparison of genome structure to other *Fusarium* species

**Figure 2:**
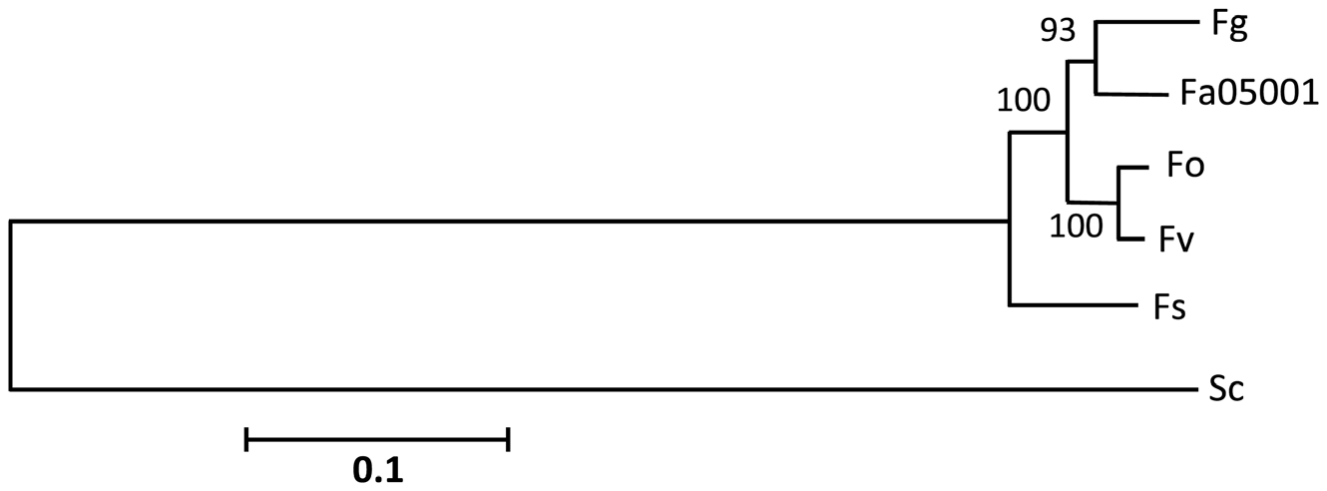
Molecular phylogenetic analysis of Fusarium species based on 69 orthologous proteins. The evolutionary history was inferred by using the Maximum Likelihood method and the tree with the highest log likelihood (−152577,9625) is shown. Bootstrap values, as percentages, are shown next to the individual branches. The tree is drawn to scale, with branch lengths measured in the number of substitutions per site. All positions containing gaps and missing data were eliminated prior to the ML analysis and the final data set contained 25535 positions.

**Figure 3:**
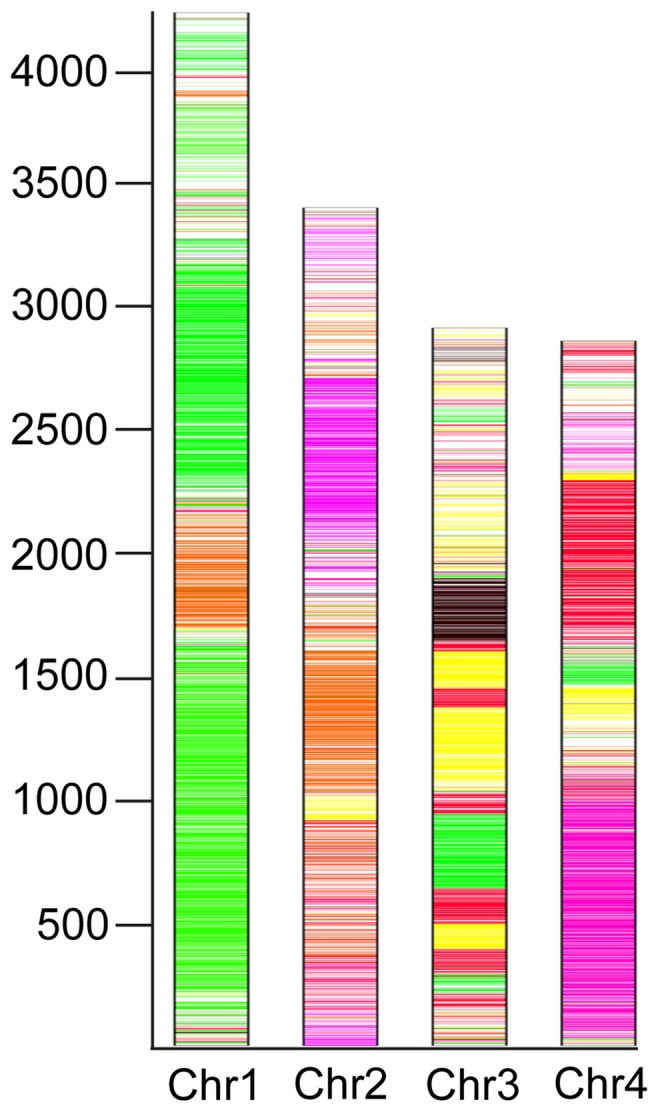
Shared gene homology map between Fa05001 and *F. graminearum* PH-1 has been created using the four defined *F. graminearum* chromosomes as templates. Genes in *F. graminearum* are coloured according to whether genes have corresponding orthologs in Fa05001, with one gene being one strip. Regions of the same color match to the same supercontigs (Figure S2 in File S1) in *F. avenaceum*. White regions represent lack of orthologs in Fa05001.

Phylogenetic analysis of genome-sequenced Fusaria based on *RPB1, RPB2*, rDNA cluster (18S rDNA, ITS1, 5.8S rDNA and 28S rDNA), *EF-1a* and *Lys2* suggest that *F. avenaceum* is more closely related to *F. graminearum*, with greater phylogenetic distance to *F. verticillioides, F. oxysporum* and *F. solani* [23], [24]. Phylogeny using *β-tub* alone [24] suggested that *F. avenaceum* is more closely related to *F. verticillioides* than the other three species. The genome data for *F. avenaceum* allowed us to reanalyse the evolutionary history within the *Fusarium* genus based on the 69 conserved proteins, initially identified by Marcet-Houben and Gabaldón [18]. The Maximum Likelihood analysis was based on 25,535 positions distributed on six super-proteins and showed that *F. graminearum* and *F. avenaceum* clustered together in 93% of 500 iterations, with *F. oxysporum* and *F. verticillioides* as sister taxa in 100% of the cases (Figure 2). *Fusarium avenaceum* scaffolds have good alignment with supercontigs of both *F. graminearum* and *F. verticillioides* with long, similar stretches of syntenic regions (Figure S5 in File S1). The synteny of *F. avenaceum* with *F. graminearum* is visualized in Figure 3, in which long stretches of genes from the same *F. avenaceum* supercontig have orthologs to neighbouring genes on *F. graminearum* chromosomes, indicating a shared genomic architecture. *F. graminearum* genes lacking orthologs in *F. avenaceum* are not distributed uniformly across the supercontig, and are mostly confined to telomeric regions, except for some at interstitial chromosomal sites. Such chromosome regions in *F. graminearum* have been shown to have a higher SNP density [22] and are influencing host specific gene expression patterns [25].

### *Fusarium avenaceum* possesses the genetic hallmarks of a heterothallic sexual life cycle

The observation of two mating-type idiomorphs was another dissimilarity between the *F. avenaceum* isolates [26], [27]. The Finnish *F. avenaceum* isolate is of the mating-type *MAT1-1*, possessing the three genes *MAT1-1-1*, *MAT1-1-2*, and *MAT1-1-3* (*FAVG1_07020*, *FAVG1_07021*, and *FAVG1_07022*), while the two Canadian isolates are of mating-type *MAT1-2*, containing the genes *MAT1-2-1* and *MAT1-2-3* (*FAVG2_03853* and *FAVG2_03854* or *FAVG3_03869* and *FAVG3_03870*). Such idiomorphs with different sets of genes has been observed previously in *Fusarium* spp. [26], [27], and surveys of *F. avenaceum* populations often find isolates evenly split between mating types [28]. The sexual stage of *F. avenaceum* has been observed [29], [30], and both *MAT1-1* and *MAT1-2* transcripts have been detected in this species under conditions favorable for perithecial production in other Fusaria [31], suggesting that *F. avenaceum* is likely capable of heterothallism. This is further supported by our data, in which a single mating-type is present in a given *F. avenaceum* isolate. This is characteristic for heterothallic fungal species, differing from homothallic species such as *F. graminearum*, which contain both mating-types in a single nucleus [32].

### Occurrence of few repetitive elements supports the hypothesis that *F. avenaceum* is sexually active

A search for repetitive elements in the Fa05001 genome (using RepeatMasker [33] with CrossMatch) identified 1.0% of the Fa05001 genome as being repetitive or corresponding to transposable elements (Table S1 in File S1). This value is comparable to the 1.12% found for *F. graminearum*, although there were differences in the distributions of the various types of genetic elements. Tad1 and MULE-MuDR transposons were more enriched in *F. avenaceum* while *F. graminearum* had higher proportions of the TcMar-Ant1 transposon and small RNA. The low level of repeats supports the hypothesis that *F. avenaceum* is sexually active in nature, as such low levels are typical for species with an active sexual cycle, such as *F. graminearum*, *F. verticillioides* [1.21% repeats] and *F. solani* [3.8% repeats], while species which rely on asexual reproduction, such as *F. oxysporum*, have higher levels [10.6% repeats]. These results could be somewhat influenced by the fact that the Fusaria genomes are generated with different technologies.

### Gene families enriched in *F. avenaceum*

**Figure 4:**
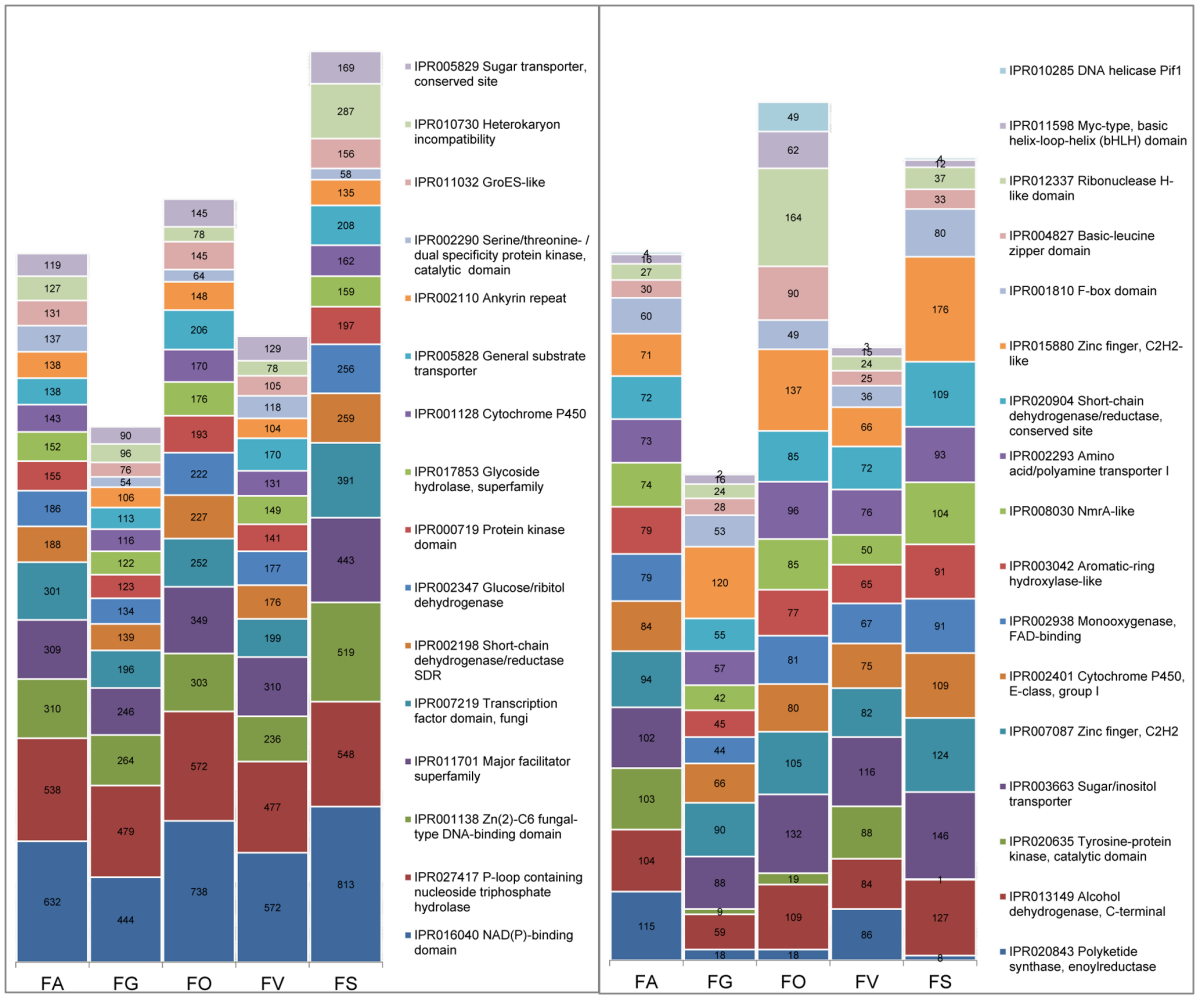
Functional analysis of Fa05001 and other sequenced Fusaria based on InterPro visualizing similarities and differences between the fungi. Categories with most differences between Fa05001 and others are presented with the number of proteins in each category. All details are listed in File S4. FA  =  *F. avenaceum* Fa05001, FG  =  *F. graminearum* PH-1, FO * =  F. oxysporum* f. sp. *lycopersici* 4287, FV  =  *F. verticillioides,* FS  =  *F. solani.*

Analysis of the predicted function of the 13217 Fa05001 gene models showed that Fa05001 contains a greater diversity of InterPro families than the other four analyzed Fusaria (Table 2, Figure 4, and File S4). Two highly enriched InterPro categories stand out in Fa05001; “Polyketide synthase, enoylreductase” (IPR020843) and “Tyrosine-protein kinase catalytic domain” (IPR020635), involved in secondary metabolism and signal transduction, respectively. With the exception of *F. solani*, Fa05001 also has the highest number of predicted transcription factors (Table S2 in File S1). Sixty-eight InterPro domains were predicted to be unique to Fa05001, including four tryptophan dimethylallyltransferase (IPR012148) proteins, commonly found in alkaloid biosynthesis. A comparison of Gene Ontology (GO) terms [34] indicated additional differences between the analyzed *Fusarium* species. Functional categories in which Fa05001 had higher numbers of proteins than the other genome-sequenced Fusaria were: “Cellular response to oxidative stress”, “Branched-chain amino acid metabolic process”, “Toxin biosynthetic process”, “Oxidoreductase activity”, “rRNA binding” and “Glutathione transferase activity” (Table S3, S4, S5 in File S1).

**Table 2:** InterproScan analysis and comparison of Fa05001 with other Fusaria.

InterPro analysis	Families	Total domains
Fa05001	5,710	27,474
*F. graminearum*	5,602	23,560
*F. oxysporum*	5,609	29,873
*F. verticillioides*	5,545	25,393
*F. solani*	5,582	30,538

Reciprocal BLAST revealed that about ¾ of the predicted proteins in Fa05001 have orthologs in *F. graminearum* (76.7%), *F. verticillioides* (76.9%), *F. oxysporum* (78.8%) and *F. solani* (74.1%) (File S5). The *F. avenaceum* proteins for which no ortholog (no hits or e-value>10^−10^) was found in the other *Fusarium* genomes were especially enriched in GO biological categories “Oxidation-reduction process”; “Toxin biosynthetic process”, “Alkaloid metabolic process”; “Cellular polysaccharide catabolic process” and “Transmembrane transport” (Table 3, Table 4, Figure S6 in File S1).

**Table 3:** Enriched biological processes in Fa05001 proteins without orthologs in other genome sequenced Fusaria (P<0.05).

*F. avenaceum vs F. graminearum*			
GO-ID	Term	FDR	P-Value
GO: 0055114	Oxidation-reduction process	2.16E-06	9.72E-10
GO: 0009820	Alkaloid metabolic process	0.35	6.33E-04
GO: 0003333	Amino acid transmembrane transport	0.37	0.001
GO: 0009403	Toxin biosynthetic process	0.71	0.004
GO: 0016998	Cell wall macromolecule catabolic process	1	0.02
GO: 0006366	Transcription from RNA polymerase II promoter	1	0.03
GO: 0044247	Cellular polysaccharide catabolic process	1	0.03
GO: 0006573	Valine metabolic process	1	0.03

*F. avenaceum vs F. verticillioides*			
GO-ID	Term		
GO: 0055114	Oxidation-reduction process	0.02	3.67E-05
GO: 0009403	Toxin biosynthetic process	0.98	0.003
GO: 0009820	Alkaloid metabolic process	1	0.01
GO: 0044247	Cellular polysaccharide catabolic process	0.02	1
GO: 0018890	Cyanamide metabolic process	1	0.02
GO: 0042218	1-aminocyclopropane-1-carboxylate Biosynthetic process	1	0.03
GO: 0006032	Chitin catabolic process	1	0.03
GO: 0006366	Transcription from RNA polymerase II promoter	1	0.04
GO: 0009636	Response to toxin	1	0.05

*F. avenaceum vs F. oxysporum*			
GO-ID	Term		
GO: 0016106	Sesquiterpenoid biosynthetic process	0.44	0.002
GO: 0009403	Toxin biosynthetic process	0.44	0.002
GO: 0055114	Oxidation-reduction process	1	0.02
GO: 0046355	Mannan catabolic process	1	0.04
GO: 0006558	L-phenylalanine metabolic process	1	0.05

*F. avenaceum vs F. solani*			
GO-ID			
GO: 0055114	Oxidation-reduction process	0.023	4.28E-05
GO: 0007155	Cell adhesion	0.13	5.08E-04
GO: 0045493	Xylan catabolic process	0.17	8.33E-04
GO: 0009403	Toxin biosynthetic process	0.8	0.005
GO: 0009820	Alkaloid metabolic process	1	0.017
GO: 0000162	Tryptophan biosynthetic process	1	0.033

See also Figure S6 in File S1.

**Table 4:** Genes annotated as reduction-oxidation process in Fa05001 proteins without orthologs in other genome sequenced Fusaria (with expect>1e-10).

Reduction-oxidation function	Genes
4-carboxymuconolactone decarboxylase	*FAVG1_07738*
ABC multidrug	*FAVG1_08208*
Acyl dehydrogenase	*FAVG1_08563, FAVG1_04763*
Alcohol dehydrogenase	*FAVG1_04699, FAVG1_12680*
Aryl alcohol dehydrogenase	*FAVG1_08747*
Bifunctional p-450: nadph-p450 reductase	*FAVG1_11632*
Transcription factor	*FAVG1_09453, FAVG1_07648*
Choline dehydrogenase	*FAVG1_12183*
Cytochrome p450 monooxygenase	*FAVG1_08576, FAVG1_13151, FAVG1_07923, FAVG1_10161, FAVG1_08627, FAVG1_02807, FAVG1_08721, FAVG1_10699*
Delta-1-pyrroline-5-carboxylate dehydrogenase	*FAVG1_04749*
Dimethylaniline monooxygenase	*FAVG1_11196*
Ent-kaurene synthase	*FAVG1_10701*
Glutaryl- dehydrogenase	*FAVG1_02842*
Homoserine dehydrogenase	*FAVG1_09776*
l-lactate dehydrogenase a	*FAVG1_08604*
Mitochondrial 2-oxoglutarate malate carrier protein	*FAVG1_08564*
Monooxygenase fad-binding protein	*FAVG1_10705*
Nadp-dependent alcohol dehydrogenase	*FAVG1_10174, FAVG1_12103, FAVG1_06950*
Nitrilotriacetate monooxygenase component b	*FAVG1_07690*
Pyoverdine dityrosine biosynthesis	*FAVG1_12703*
Salicylate 1-monooxygenase	*FAVG1_09825*
Salicylaldehyde dehydrogenase	*FAVG1_09676*
Short-chain dehydrogenase	*FAVG1_07710, FAVG1_12690, FAVG1_04239, FAVG1_10281, FAVG1_08519*

See also Figure S6 in File S1.

### The secretome of *F. avenaceum*

The interplay between the invading fungus and the host plant occurs mainly in the extra-cellular space. The proportion of genes encoding predicted secreted proteins in the Fa05001 genome (File S6, Table S6 in File S1) is remarkably high (∼20%; 2,580 proteins) as compared to plant pathogens such as *F. graminearum* (11%) and *Magnaporthe grisea* (13%), saprophytes such as *Neurospora crassa* (9%) and *Aspergillus nidulans* (9%) [22], and the insect pathogen *Cordyceps militaris* (16%) [35]. The secretome appears particularly enriched in proteins involved in redox reactions (Figure S7 in File S1). Inspecting the *F. avenaceum* proteins with no orthologs in other Fusaria (1223 proteins from Figure S6 in File S1), we found that 36% were predicted to be secreted.

Small cysteine-rich proteins (CRPs) can exhibit diverse biological functions, and some have been shown to play a role in virulence, including Avr2 and Avr4 in *Cladosporium fulvum* [36] and the Six effectors in *F. oxysporum* f. sp. *lycopersici* [37]. Other reported functions for CRPs have been adherence [38], antimicrobiosis [39] and carbohydrate binding activity that interferes with host recognition of the pathogen [40]. We found 19 candidates in Fa05001 containing more than four cysteine residues, and an additional 55 containing less than four cysteine residues, but with significant similarity to CRP HMM models (File S7, Table S7 in File S1). Of the predicted *F. avenaceum* CRPs, several are also found in other sequenced *Fusarium* species, but only two were noted with a putative function: CRP5760, with similarity to lectin-B, and CRP5810, a putative chitinase 3.

### Metabolic profiling of *F. avenaceum*

A determination of secondary metabolites produced by these three *F. avenaceum* strains was performed on agar media, additionally Fa05001 was also grown *in planta* (barley and oat). Extraction of *F. avenaceum* cultures grown on PDA and YES solid media revealed the presence of 2-amino-14,16-dimethyloctadecan-3-ol, acuminatopyrone, antibiotic Y, fusaristatin A, aurofusarin, butenolide, chlamydosporols, chrysogine, enniatin A, enniatin B, fusarin C, and moniliformin (Table 5). These metabolites have previously been reported from a broad selection of *F. avenaceum* strains [5], [16], [41], [42] whereas the following metabolites previously reported from *F. avenaceum* were not detected in the present study: beauvericin [43], fosfonochlorin [44], diacetoxyscirpenol, T-2 toxin and zearalenone [45]–[50]. These reports are based on single observations using insufficient specific chemical methods that could yield false positive detection and/or poor fungal identification and no deposition of the strain in a collection for verification. The lack of zearalenone production agrees with the finding that none of the three *F. avenaceum* genomes described here contains the genes required for its production [51], [52].

**Table 5:** Metabolic profiling of the three *F. avenaceum* strains.

	FaLH03		FaLH27		Fa05001				Other strains
	YES	PDA	YES	PDA	YES	PDA	Barley	OAT	*F. avenaceum*
**PKS**									
2-Amino-14,16-dimethyloctadecan-3-ol	+	+	+	+	+	+	ND	ND	+
Acuminatopyrone	+	+	ND	ND	+	+	ND	ND	+
Antibiotic Y	+	+	+	+	+	+	ND	ND	+
Aurofusarin	+	+	+	+	+	+	+	+	+
Fuscofusarin	+	+	+	+	+	+	ND	ND	NA
Moniliformin	+	+	+	+	+	+	NA	NA	+
Chlamydosporols	+	+	ND	ND	+	+	ND	ND	+
**NRPS and mixed NRPS-PKS**									
Butenolide	ND	ND	ND	ND	+	+	+	ND	+
Chrysogine	+	+	+	+	+	+	+	+ but 10 × lower than barley	+
Visoltricin	ND	ND	ND	ND	ND	ND	ND	ND	+ (by UV-Vis)
Fusarins C and A	+	ND	+	ND	+	+	ND	ND	+
Enniatins A's and B's	+	+	+	+	+	+	+	+ but 100 × lower than barley	+
Beauvericin	ND	ND	ND	ND	ND	ND	ND	ND	ND
Apicidin	ND	ND	ND	ND	ND	ND	ND	ND	+
Fusaristatins	+	+	+	+	+	+	+	Trace	+
Fusarielin A	ND	ND	ND	ND	ND	ND	ND	ND	ND
Ferricrocin	+	ND	+	ND	+	+	ND	ND	NA
Fusarinines	ND	ND	ND	ND	ND	ND	ND	ND	NA
JM-47	+	+	+	+	+	+	+	+	NA
Malonichrome	+	ND	+	ND	+	ND	ND	ND	NA
**Other**									
Fosfonochlorin	ND	ND	ND	ND	ND	ND	ND	ND	NA
Unknown 26 (NRPS)					+	+	+	+	
Fusarium unknown 31 (NRPS)					+	+	+	+	
ND not detected									
NA not analyzed									

Preliminary genomic analysis had predicted the production of ferricrocin, malonichrome, culmorin, fusarinine and gibberellins [23], but chemical analysis only verified the production of the first two metabolites. The polyketide fuscofusarin (an aurofusarin analogue or intermediate in the biosynthetic pathway) was detected for the first time in *F. avenaceum*. Furthermore, fusaristatin A was found for the first time to be produced *in planta* during climate chamber experiments, and was generally found in *F. avenaceum-*infected barley, but only in trace amounts in oat. In addition, the tetrapeptide JM-47, an HC-toxin analogue reported from an unidentified *Fusarium* strain [53], was detected from all three strains in all cultures, including in barley and oats in climate chamber experiments. Apicidins have been detected in other strains of *F. avenaceum* cultured on YES agar (unpublished results), whereas these compounds were not detected in cultures of the three sequenced strains. Further characterization of the metabolomes on PDA and YES media revealed three major peaks in the ESI^+^ chromatograms, which were also detected in the barley and oat extracts, that could not be matched to known compounds in Antibase [54]. Since these novel compounds are produced *in planta*, they are candidates for novel virulence factors.

### Large potential for secondary metabolite production

The three *F. avenaceum* genomes encode 75 (Fa05001), 77 (FaLH03) and 80 (FaLH27) key enzymes for the production of secondary metabolites, exhibiting a far greater biosynthetic potential than the known secondary metabolites produced by the species. These numbers include genes for 25–27 iterative type I polyketide synthases (PKSs), 2–3 type III PKSs, 25–28 non-ribosomal peptide synthases (NRPSs), four aromatic prenyltransferases (DMATS), 12–13 class I terpene synthases (head-to-tail incl. cyclase activity), two class I terpene synthase (head-to-head) and four class II terpene synthases (cyclases for the class I terpene synthase head-to-head type) (Table 6).

**Table 6:** The number of identified signature genes for secondary metabolism in the three *F. avenaceum* strains compared to the other public *Fusarium* genome sequences.

	PKS I	PKS III	NRPS	TS I HT*	TS I HH**	TS II***	DMATS
**Fa05001**	25 (12)	2 (1)	25 (9)	13 (2)	2(0)	4(0)	4(2)
**FaLH03**	25 (12)	3 (2)	26 (10)	12 (1)	2(0)	4(0)	4(2)
**FaLH27**	27 (14)	2 (1)	28 (12)	13 (1)	2(0)	4(0)	4(2)

The number of genes that are unique for *F. avenaceum* is given in the parentheses. Gene classes: type I iterative PKS (PKS I), type III PKS (PKS III), non-ribosomal peptide synthetases (NRPS), terpene synthase class I head-to-tail type (TS I HT), terpene synthase class I head-to-head type (TS I HH), terpene synthases class II (TS II) and aromatic prenyltransferases (DMATS).

*incl. ERG20, COQ1, BTS1,

**incl. ERG9,

***incl. ERG7.

**Figure 5:**
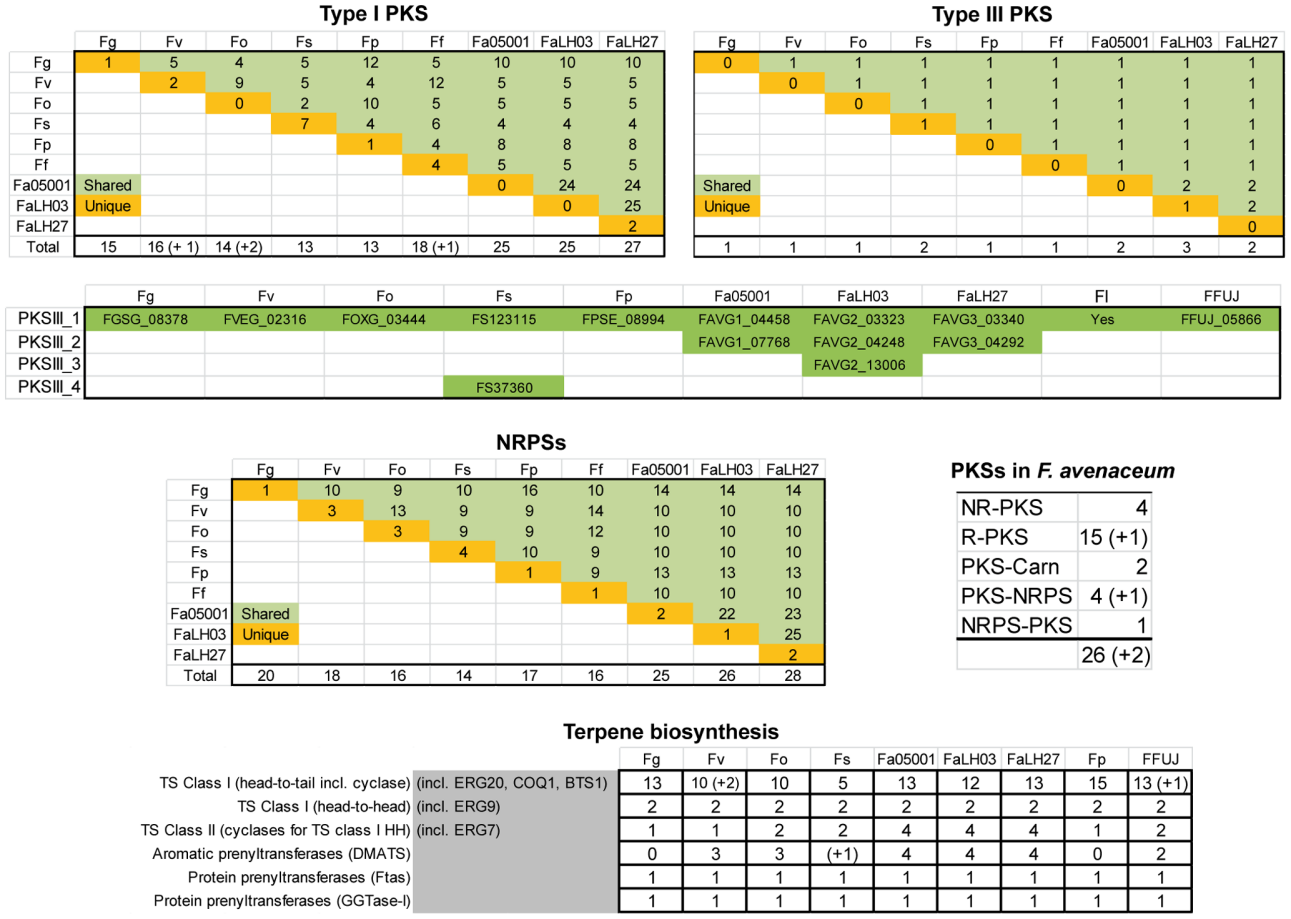
Shared and unique polyketide synthase (PKS), non-ribosomal peptide synthetases (NRPS) and terpene cyclase (TC) encoding genes in public available Fusaria genomes. Green and yellow boxes are the number of shared and unique genes, respectively. *Fg*  =  *F. graminearum, Fv*  =  *F. verticillioides, Fo  =  F. oxysporum*, *Fs*  =  *F. solani, Fp*  =  *F. pseudograminearum, Ff*  =  *F. fujikuroi* and *Fa05001, FaLH03* and *FaLH27*  =  *F. avenaceum*.

The number of type I PKSs is surprisingly high, considering that the six other public *Fusarium* genomes (*F. graminearum, F. verticillioides, F. oxysporum, F. solani, F. pseudograminearum* and *F. fujikuroi*) only encode between 13 and 18 type I PKSs. The three *F. avenaceum* genomes share a core set of 24 type I PKS, and the individual isolates also encode unique type I PKS: oPKS47 (FAVG1_08496) is unique to Fa05001, while the two Canadian isolates share a single oPKS53 (FAVG2_01811, FAVG3_01846) and FaLH27 has two additional PKSs, oPKS54 (FAVG3_02030) and oPKS55 (FAVG3_06174). Of the 55 different type I PKSs described in the seven *Fusarium* genomes, only two (oPKS3 and oPKS7) are found in all species. Orthologs for 12 of the type I PKSs found in *F. avenaceum* could be identified in one or several of the publicly available *Fusarium* genomes, and hence 16 are new to the genus (Figure 5). None of these 16 have obvious characterized orthologs in other fungal genomes or in the GenBank database. The PKSs with characterized orthologs within the *Fusarium* genus includes the PKSs responsible for formation of fusarubin (oPKS3), fusaristatins (oPKS6), fusarins (oPKS10), aurofusarin (oPKS12) and fusaristatin A (oPKS6) [16], [55]–[57]. Prediction of domain architecture of the 28 PKSs found in *F. avenaceum* showed that 17 belong to the reducing subclass, four to the non-reducing subclass, two are PKSs with a carboxyl terminal Choline/Carnitine O-acyltransferase domain, four are PKS-NRPS hybrids and one is a NRPS-PKS hybrid. The iterative nature of this enzyme class makes it impossible to predict the products of these enzymes without further experimental data; this research has been initiated. The *F. avenaceum* genomes also encode type III PKSs, a class that among fungi was first described in *Aspergillus oryzae* [58], and recently in *F. fujikuroi* [59]. The FaLH03 isolate possesses three proteins (oPKSIII-1 to oPKSIII-3), while the two other isolates only have two (oPKSIII-1 and oPKSIII-3).

NRPSs provide an alternative to ribosomal-based polypeptide synthesis and in addition allow for the joining of proteinous amino acids, nonproteinous amino acids, α-hydroxy acids and fatty acids as well as cyclization of the resulting polypeptide [60]. The non-ribosomal peptide group of metabolites includes several well characterized bioactive compounds, such as HC-toxin (pathogenicity factor) and apicidin (histone deacetylase inhibitor) [61], [62]. Of the 30 unique NRPSs encoded by the three *F. avenaceum* isolates, 16 are novel to the *Fusarium* genus, and include seven mono-modular and nine multi-modular NRPS, with between 2 and 11 modules. Of these, NRPS41 (FAVG1_08623, FAVG2_11354 and FAVG3_11434) is a likely ortholog to gliP2 (similar to gliotoxin synthetase) from *Neosartorya fischeri* (Genbank accession no. EAW21276), sharing 75% amino acid identity. The other 14 NRPSs are orthologs to previously reported proteins in other *Fusarium* species [63], and include the three NRPSs responsible for the formation of the siderophores malonichrome (oNRPS1), ferricrocin (sidC, oNRPS2) and fusarinine (sidA, oNRPS6).

**Figure 6:**
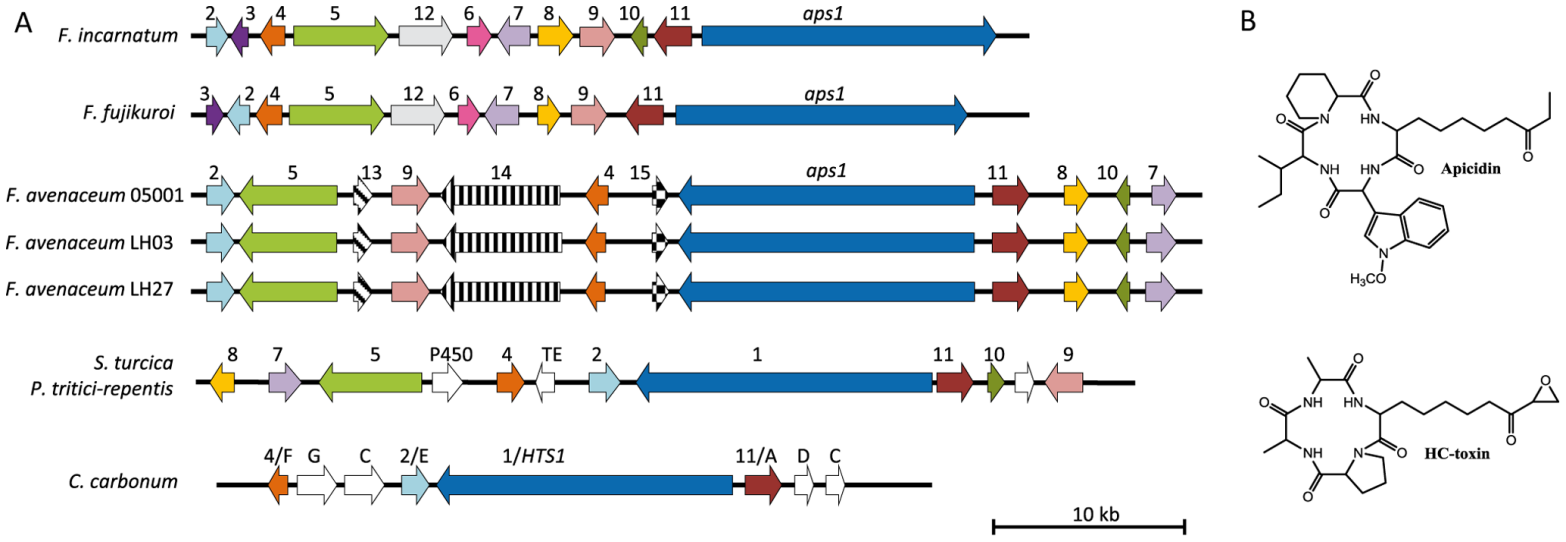
Apicidin-like gene cluster (oNRPS31) in the three *F. avenaceum* strains (Fa05001_Scaffold14, FaLH03_contig11, FaLH27_contig13) compared to the characterized apicidin gene cluster from *F. incarnatum* and the HC-toxin gene clusters from *Cochliobolus carbonum*, *Pyrenophora tritici-repentis* and *Setosphaeria turcica* (A). The genes are colored based on homology across the species. Chemical structure of apicidin and HC-toxin (B). See Table S8 in File S1 for further details.

All three strains encoded oNRPS31 which shows a significant level of identity to the apicidin NRPS (APS1) described in *F. incarnatum* and *F. fujikuroi* [59], [64], and the HC-toxin NRPS (HTS1) from *Cochliobolus carbonum* SB111, *Pyrenophora tritici-repentis* and *Setosphaeria turcica* [65], [66]. It has not yet been investigated whether NRPS31 is involved in the production of JM-47. Alignment of the genomic regions surrounding oNRPS31 with the APS1 and HTS1 clusters, showed that the three *F. avenaceum* strains encode nine of the twelve APS proteins found in the other two Fusaria, but lack clear orthologs encoding APS3 (pyrroline reductase), APS6 (O-methyltransferase) and APS12 (unknown function). The *APS*-like gene cluster in *F. avenaceum* has undergone extensive rearrangements, resulting in the loss of the three *APS* genes and gain of three new ones (*APS13-15*) (Figure 6 and Table S8 in File S1). Of these, *APS14* (*FAVG1_08581*, *FAVG2_02887* and *FAVG3_02926*) encodes a fatty acid synthase β subunit, which shares 60% identity with the FAS β subunit (encoded by *FAVG1_03575*, *FAVG2_06952* and *FAVG3_07032*) involved in primary metabolism. APS14 likely interacts with APS5 (fatty acid synthase α subunit) to form a functional fungal FAS (α6β6) responsible for the formation of the decanoic acid core of (S)-2-amino-8-oxodecanoic acid, proposed by Jin and co-workers [64] to be incorporated into apicidin by APS1. It has previously been hypothesized that APS5 (FAS α) interacts with the FAS α unit from primary metabolism to fulfill this role [64], however, the new model implies that the *F*. *incarnatum* genome encodes an unknown APS14 ortholog (no genome sequence available). The *F. fujikuroi* genome does not contain an APS14 ortholog (TBLASTn against the genome), but apicidin F has been detected in this species [59], [67]. Alignment of the apicidin and HC-toxin gene clusters confirmed the observations made by Manning *et al.* [66] and Condon *et al.* [65], regarding the similarities of the two types of gene clusters, which yield very different products (Figure 6).

Dimethylallyltransferase and indole-diterpene biosynthesis proteins are common in the production of bioactive compounds in endophytes [68], [69]. The three *F. avenaceum* genomes encoded four tryptophan dimethylallyltransferase (DMATS), aromatic prenyltransferases, typically involved in alkaloid biosynthesis or modification of other types of secondary metabolites [68]. This is one more than is found in *F. verticillioides* and *F. oxysporum*, and two more than found in *F. fujikuroi.* One putative indole-diterpene biosynthesis gene was found in all three *F. avenaceum* genomes (*FAVG1_08136*, *FAVG2_00906* and *FAVG3_00934*).

The three *F. avenaceum* genomes are also rich in genes involved in terpene biosynthesis. In the case of the class I terpene synthase, responsible for the head-to-tail joining of isoprenoid and extended isoprenoid units and eventually cyclization, the three genomes encode 12–13 enzymes, of which three were putatively identified as being involved in primary metabolism (ERG20, COQ1, BTS1). In the case of the head-to-head class I systems, *F. avenaceum* encodes two enzymes, similar to the other fully genome-sequenced Fusaria, of which one gene encodes ERG9 and the other is involved in carotenoid biosynthesis. Cyclization of the formed head-to-head type product, if such a reaction occurs, is probably catalyzed by a class II terpene synthase/cyclase, of which *F. avenaceum* encodes four, including an ERG7 ortholog. This is more than the other Fusaria spp. genomes, as *F. graminearum*, *F. pseudograminearum* and *F. verticillioides* only have ERG7, while *F. solani*, *F. oxysporum* and *F. fujikuroi* have two.

**Figure 7:**
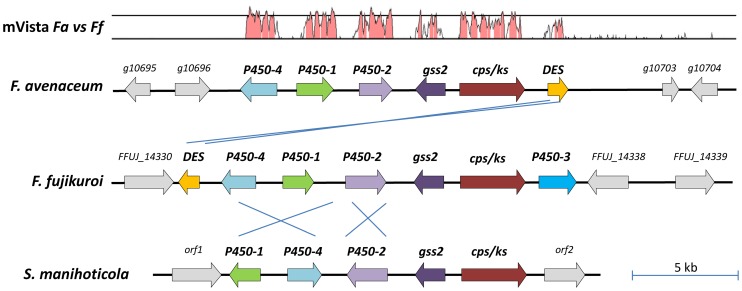
Architecture of the gibberellic acid (GA) gene clusters from *F. fujikuroi* MP-A, *F. avenaceum* and *Sphaceloma manihoticola*. The gene cluster and surrounding genes are identical in the three *F. avenaceum* strains and only Fa05001 is shown (FaLH03 cluster: *FAVG2_04186 - FAVG2_04192* and FaLH27 cluster: *FAVG3_04219 - FAVG3_04224*). The mVista trace shows the similarity over a 100 bp sliding windows (Shuffle-LAGAN plot) between the *F. avenaceum* and *F. fujikuroi* clusters, bottom line  = 50% and second line  = 75% identity. Genes: *gss2*  =  geranylgeranyldiphosphate synthase, *cps/ks*  =  copalyldiphosphate/ent-kaurene synthase, *P450-4*  =  *ent-*kaurene oxidase, *P450-1*  =  GA_14_ synthase, *P450-2*  =  C20-oxidase, *P450-3*  =  13-hydroxylase and *DES*  =  desaturase. Note that the intergenic regions are unknown for the *S. manihoticola* GA cluster, while the size of these regions is not to drawn to size.

One of the four Type II terpene synthase encoding genes (TS_II_01: *FAVG1_10701*, *FAVG2_04190* and *FAVG3_04223*) shared by all three *F. avenaceum* strains was found to be orthologous to the gibberellic acid (GA) copalyldiphosphate/ent-kaurene synthase (cps/ks) from *F. fujikuroi* (Table S9 in File S1). Previously, the ability to synthesize the GA group of diterpenoid plant growth hormones in fungi has only been found in *F. fujikuroi* mating population A, *Fusarium proliferatum, Sphaceloma manihoticola* and *Phaeosphaeria sp.* strain L487 [70]–[74]. Biosynthesis of GA was first thoroughly characterized in *F. fujikuroi* and depends on the coordinated activity of seven enzymes, encoded by the GA gene cluster, that convert dimethylallyl diphosphate (DMAPP) to various types of gibberellic acids, with the main end-products being GA_1_ and GA_3_ [70]. *S. manihoticola*'*s* GA biosynthesis ends at the intermediate GA_4_ due to the lack two of genes (DES and P450-3), compared to *F. fujikuroi*, responsible for converting GA_4_ to GA_7_, GA_3_ and GA_1_ [73]. Analysis of the genes surrounding the *F. avenaceum* CPS/KS encoding gene showed that six of the seven genes from the *F. fujikuroi* GA gene cluster are also found in all three *F. avenaceum* genomes, with only P450-3 (C13-oxidase) missing (Figure 7). The architecture of the GA cluster in *F. avenaceum* is highly similar to the *F. fujikuroi* cluster, and a single inversion in five of the six genes can explain the relocation of the desaturase (*des*) encoding gene. The inversion could potentially have involved the P450-3 gene and resulted in the disruption of its coding sequence, however the shuffle-LAGAN analysis (Figure 7) and dot plot showed that this has not been the case. The missing gene is not found elsewhere in the genome based on a tblastn search. The presence of six of the seven GA biosynthesis genes suggest that *F. avenaceum* has the potential to produce all the GA's up to G_4_ and G_7_, but lack the ability to convert these into the GA_1_ and GA_3_. None of the three *F. avenaceum* isolates have been reported to produce this plant growth hormone.

In summary, *F. avenaceum* has a very large potential for producing secondary metabolites belonging to the PKS, NRPS and terpene classes, with a total of 75–80 key enzymes, see File S8 that summarizes all orthology groups. However, it is expected that multiple enzymes will participate in a single biosynthetic pathway, thereby reducing the potential number of final metabolites. It is possible that some of the metabolites function as virulence factors during infection; however systematic deletion of all PKS encoding genes in *F. graminearum* showed that none of the 15 PKSs in this fungus had significant effect on virulence [75]. It is therefore more likely that at least some of the secondary metabolites function as antibiotics towards competing microorganisms in the diverse set of niches that the species inhabits. When plotting the PKSs and NRPSs on the 11 supercontigs, areas with higher numbers of SNPs, insertions, or deletions between the three strains, were also more populated with secondary metabolite genes (Figure 1), as seen in other Fusaria [22].

### Transcriptomics of *F. avenaceum* in barley

To increase our understanding of *F. avenaceum* behaviour *in planta*, we performed RNA-seq on *F. avenaceum*-inoculated barley. Table S10 in File S1 shows a list of *F. avenaceum* genes with the most stable and significant expression (FDR<0.05). Due to putative false positive genes expressed in the host, we applied high stringency in the analysis, and only genes found expressed at 14 days post inoculation (dpi), and which were absent in control, were considered. Genes involved in stress related responses, especially oxidative stress (as defined in the fungal stress response database [76]) were highly represented. This strongly supports our hypothesis formulated from the comparative genomic analysis that the broad host range of *F. avenaceum* is likely due to a generalized mechanism allowing it to cope with and overcome the innate immune response of plants, such as the generation of reactive oxygen species (ROS) [77].

Genes involved in signal transduction were also overrepresented in the transcriptome, including GO categories for GTP binding, ATP binding, calcium ion binding, and membrane activity (Figure S8 in File S1, File S8). Approximately 33% of all proteins predicted in the genome with Interpro “Tyrosine-protein kinase, catalytic domain” (IPR020635) were found in the transcriptome. This was one of the most highly enriched *F. avenaceum* categories when compared to other *Fusarium* genome sequences, and the *in planta* transcriptome hence supports the comparative results from the genome analysis. During plant infection, fungi need to monitor the nutrient status and presence of host defenses, and respond to or tolerate osmotic or oxidative stress, light and other environmental variables [78]. Stress-signaling/response genes of fungal pathogens are known to play important roles in virulence, pathogenesis and defense against oxidative burst (rapid production of ROS) from the host [79], [80]. It is plausible to predict that tyrosine-protein kinases assist in the stress related response. There is a tendency that *F. avenaceum* isolates from one host can be pathogenic on other distantly related plants [11]. This is in contrast to, for example, *F. oxysporum*, a pathogen with a remarkably broad host range at the species level, but where individual isolates often cause disease only on one or a few plant species [81]. Our results support the chameleon nature of *F. avenaceum*, as it is capable of adapting to diverse hosts and environments. This lack of host specialization is likely to be a driving force in *F. avenaceum* evolution. Apart from the general functional categories, the *in planta* transcriptome of *F. avenaceum* also revealed many orthologs of *F. graminearum* pathogenicity and virulence factors, especially those involved in signal transduction and metabolism (Table S11 in File S1, [82]).

## Conclusions

In summary, the comparative genomic analyses of *F. avenaceum* to other Fusaria point out several functional categories that are enriched in this fungal genome, and which indicate a great potential for *F. avenaceum* to sense and transduce signals from the surroundings, and to respond to the environment accordingly. *Fusarium avenaceum* has a large potential for redox, signaling and secondary metabolite production, and 20% of all predicted proteins were considered to be secreted. This could suggest that interaction with plant hosts is predominantly in the extracellular space. These genome sequences provide a valuable tool for the discovery of genes and mechanisms for bioactive compounds, and to increase our knowledge of the mechanisms contributing to a fungal lifestyle on diverse plant hosts and in different environments.

## Materials and Methods

### Sequencing, assembly, gene prediction and annotation

*Fusarium avenaceum* isolate Fa05001 (ARS culture collection: NRRL 54939, Bioforsk collection: 202103, DTU collection: IBT 41708) was isolated from barley in 2005 in Finland [83]. The strain was grown on complete medium [84] at room temperature for three days on a shaker, before mycelium was vacuum filtered and harvested for storage at −80°C. DNA was isolated using the Qiagen DNeasy Plant Maxi. The sequencing and assembly was performed by Eurofins MGW, using a combination of shotgun (1.5 plate) and paired-end (¼ plate of 3 kb, and ¼ plate of 20 kb) 454 pyrosequencing. Newbler 2.6 (www.roche.com) was used for automatic assembly, and gap closure and further assembly was performed using GAP4 (Version 4.4; 2011, http://www.gap-system.org), a total of 50 primer pairs, and manual editing. PCR products from gaps were Sanger sequenced in both directions, and 38 PCR products were successfully integrated into the assembly. These approaches significantly improved the results, starting from 502 contigs and 89 scaffolds after automatic annotation to 110 contigs and 83 scaffolds (Table 1).

Two Canadian *F. avenaceum* strains, FaLH03 (spring wheat, New Brunswick) and FaLH27 (winter wheat, Nova Scotia), were isolated from wheat samples harvested in 2011 (Canadian Grain Commission, Winnipeg, MB) and deposited in the Canadian Collection of Fungal Cultures (AAFC, Ottawa, ON) with the strain designations DAOM242076 and DAOM242378, respectively. Species identification was confirmed by sequencing the *tef1-α* gene [85]. Strains were single-spored prior to any analysis and confirmed to retain virulence towards durum wheat. After growth for 3 days in glucose-yeast extract-peptone liquid culture, mycelia was collected and freeze-dried. Genomic DNA was extracted using the Nucleon Phytopure genomic DNA extraction kit (GE Healthcare, Baie d'Urfe, Québec) and then used to prepare an Illumina TruSeq library. Each library was sequenced in a single lane on an Illumina HiSeq platform (100 bp paired-end) at the Génome Québec Innovation Centre (Montréal, Québec), yielding 99,386,445 and 233,211,138 reads for FaLH03 and FaLH27, respectively. Reads were assembled in CLC Genomics Workbench 6.0.1. The higher order of the obtained scaffolds in the three isolates was resolved through comparison between the strains. The contigs from the Canadian isolates were ordered to each other by ABACAS [86]. A reiterative reordering approach was used to generate a stable order of the contigs. The successful reordering is illustrated in the alignment of the ordered contigs by MUMMER [87] (Figure S2 in File S1). The scaffolds from the Fa05001 were then ordered according to the Canadian isolates by ABACAS, and aligned to the Canadian isolates by ‘MUMMER’ (Figure S2 in File S1). Contig/scaffold overlaps and boundaries in the ‘MUMMER’ alignments were used to determine the higher order assembly.

Gene prediction was performed with Augustus v2.5.5 (Fa05001) and v2.6 (FaLH03, FaLH27) [88], using default settings and *F. graminearum* as a training set. Protein sequences were annotated and enrichment analysis of gene ontology categories were compared using Blast2GO [89]. Table 1 shows the general statistics of the three genome sequences.

The species phylogenies were constructed based on 69 orthologous proteins from the included species. The 69 protein sets were first aligned individually using MUSCLE with default parameters [18], then manually inspected and concatenated. These super-protein alignments were then analyzed using MEGA6.0 by first identifying the best substitution model and then inferring the evolutionary history of the species using the Maximum Likelihood method, Nearest-Neighbour-Interchange, [90] using the LG+(F) substitution model, uniform substitution rate, removal of all positions with gaps and missing data and bootstrapping with 500 iterations.

### Functional analysis and orthology prediction

For comparative genomics analysis, the previously sequenced genomes of *F. graminearum*, *F. verticillioides*, *F. oxysporum* f. sp. *lycopersici* 4287 (*Fusarium* Comparative Sequencing Project, Broad Institute of Harvard and MIT, http://www.broadinstitute.org/) and *F. solani* [91] were re-annotated using Blast2GO [89] concurrently with Fa05001. A functional comparison was performed using the Gene Ontology (GO) categories Biological Process, Molecular Function, and Cellular Compartments at level six, and Interpro. To compare transcription factors, we used the Interpro list from the Fungal Transcription Factor Database [92]. To identify orthologs, protein sets were compared in both directions with BLASTp to identify the best reciprocal hit for each individual protein with expectation values less than 1e-10. RepeatMasker [11] was used on all species to find repetitive sequences in the *Fusarium* genomes, using CrossMatch (http://www.phrap.org/phredphrap/general.html). BLASTn was used to compare gene sequences between the three *F. avenaceum* genomes.

### Electrophoretic karyotyping

Plugs containing 4×10^8^/ml protoplasts were loaded on a CHEF gel (1% FastLane agarose [FMC BioProducts, Rockland, Maine] in 0.5×TBE) and ran for 255 hours, using switch times between 1200–4800 s at 1.8 V/cm. Chromosomes of *Schizosaccharomyces pombe* and *Hansenula wingei* were used as a molecular size marker (BioRad). Chromosomes separated in the gel were blotted to Hybond-N+. Southern hybridization was done overnight at 65°C using the CDP star method (GE Healthcare). A 350 bp *Hin*dIII-*Eco*RI fragment from plasmid pNLA17 was used as a probe, containing the *F. oxysporum* telomeric repeat TTAGGG 18 times.

### Prediction of putative secretome

To determine the putative secretome, we employed a pipeline consisting of the following: A combination of WolfPsort (http://wolfpsort.org/), IPsort (http://ipsort.hgc.jp/) and SignalP4.1 [93] to identify subcellular localization and/or signalP motifs of all *F. avenaceum* Fa05001 proteins. Then, we used TMHMM [94] to predict all transmembrane domains. The secretome fasta file with the sequences was created with GPRO [95], with proteins predicted by either the PSORT tools or SignalP that does not contain transmembrane domains. Of the whole set of 13217 predicted proteins in Fa05001, a subset of 2580 sequences has been determined as the putative secretome of *F. avenaceum* (File S6).

### Prediction of cysteine-rich proteins

Prediction of putative cysteine-rich proteins CRPs was based on a previously described approach [96]. In brief, this method is based on their expected sequence characteristics, with predicted small open reading frames (ORFs) (20 to 150 amino acids), containing at least four cysteine residues and a predicted signal peptide from the secretory pathway. The 83 Fa05001 scaffolds were used as input against GETORF available in the EMBOSS package [97]. We obtained 565.652 ORFs, which were translated to proteins using the tool Transeq [97]. All proteins were separated into two files (more/less than four cysteine residues). We downloaded a collection of 513 HMM profiles based on CRP models [98], and then created a single HMM database file that was formatted with HMMER3 [99] and used as subject against a HMMER comparison with the two files obtained. Nineteen candidates with more than four cysteine residues were predicted as CRP, and 55 additional sequences with less than four cysteine residues but with significant similarity to particular CRP HMM models were also included (Table S7 in File S1, File S7). We compared these to the supercontigs and scaffolds of the genome sequenced *Fusarium* spp. using BLASTn and TBLASTx, bit-score>50, and BLASTp in NCBI.

### Identification of secondary metabolites

The three *F. avenaceum* strains were grown at 25°C in darkness for 14 days as triple point inoculations on Potato Dextrose Agar (PDA, [100]) and Yeast Extract Sucrose agar (YES, [100]). The metabolites of the strains were extracted using a modified version of the micro-scale extraction procedure for fungal metabolites [101]. Six 5-mm plugs from each plate taken across the colonies were transferred to 2 mL HPLC vials and extracted with 1.2 mL methanol:dichloromethane:ethyl acetate (1∶2∶3 v/v/v) containing 1% (v/v) formic acid. After 1 hr in an ultrasonication bath, extracts were evaporated with nitrogen, the residue dissolved in 150 µL acetonitrile:water (3/2 v/v) and filtered through a standard 0.45-µm PTFE filter.

Barley and oat samples (all in biological triplicates), including none-inoculated samples from climate chamber experiments described below were ground in liquid nitrogen and 50 mg extracted with 1 mL of 50% (vol) acetonitrile in water in a 2 mL Eppendorf tube. Samples were placed in an ultrasonication bath for 30 min, centrifuged at 15,000 g, and the supernatant was transferred to a clean 2 mL vial that was loaded onto the auto sampler prior to analysis. UHPLC-TOFMS analysis of 0.3–2 µL extracts were conducted on an Agilent 1290 UHPLC equipped with a photo diode array detector scanning 200–640 nm, and coupled to an Agilent 6550 qTOF (Santa Clara, CA, USA) equipped with a dual electrospray (ESI) source [102]. Separation was performed at 60°C at a flow rate of 0.35 mL/min on a 2.1 mm ID, 250 mm, 2.7 µm Agilent Poroshell phenyl hexyl column using a water-acetonitrile gradient solvent system, with both water and acetonitrile containing 20 mM formic acid. The gradient started at 10% acetonitrile and was increased to 100% acetonitrile within 15 min, maintained for 4 min, returned to 10% acetonitrile in 1 min. Samples were analyzed in both ESI^+^ and ESI^−^ scanning *m/z* 50 to 1700, and for automated data-dependent MS/MS on all major peaks, collision energies of 10, 20 and 40 eV for each MS/MS experiment were used. An MS/MS exclusion time of 0.04 min was used to get MS/MS spectra of less abounded ions.

Data files were analyzed in Masshunter 6.0 (Agilent Technologies) in three different ways: i) *Aggressive dereplication* [103] using lists of elemental composition and the *Search by Formula* (10 ppm mass accuracy) of all described *Fusarium* metabolites as well as restricted lists of only *F. avenaceum* and closely related species; ii) Searching the acquired MS/MS spectra in an in-house database of approx. 1200 MS/MS spectra of fungal secondary metabolites acquired at 10, 20 and 40 eV [102]; iii) all major UV/Vis and peaks in the base peak ion chromatograms not assigned to compounds (and not present in the media blank samples) were also registered. For absolute verification, authentic reference standards were available from 130 *Fusarium* compounds and additional 100 compounds that have been tentatively identified based on original producing strains using UV/Vis, LogD and MS/HRMS [102]–[104].

### Identification of secondary metabolite genes

Type I iterative polyketide synthase (PKS), type III PKS, non-ribosomal peptide synthase (NRPS), aromatic prenyltranferase (DMATS) and class I & II terpene synthase encoding genes were identified by BLASTp using archetype representatives for the six types of genes [105]. Identification of orthologous genes was further supported by comparison to the genomic DNA, using the shuffle LAGAN algorithm with default settings [106], [107]. Functional protein domains were identified using the NCBI CDD and pfam databases [108]. Domains specific to non-reducing PKSs, e.g. ‘Product template’ (PT) and ‘Starter Acyl-Transferase’ (SAT), were inferred via multiple sequence alignment with the bikaverin PKS (PKS16), which was one of the founding members of the domain group [109]. The nomenclature for PKS and NPS follows that which was introduced by Hansen et al. [63], as indicated by the use of the oPKSx and oNRPSx name, where the prefix ‘o’ signals that it refers to orthology-groups rather than the original overlapping names schemes used previously in each species. Following the idea regarding transparency in the names, introduced by Hansen and co-works, we applied a similar nomenclature scheme to the type III PKSs (oPKSIII_x) and the various enzyme classes involved in terpene biosynthesis: Terpene Synthase class I head-to-tail (oTS-I-HT_x), Terpene Synthase class I head-to-head (oTS-II-HH_x) and Terpene Synthase class II (oTS-II-x).

### Climate chamber infection experiment

Fa05001 was grown on mung bean agar [110] for three weeks at room temperature under a combination of white and black (UVA) light with a 12 h photoperiod. Macroconidia were collected by washing the agar plate with 5 mL sterile distilled water, and diluted with 1.5% carboxymethylcellulose solution to a concentration of 5×10^4^ conidia/mL for inoculation. Conidial concentration was determined using a Bürker hemacytometer.

Barley (*Hordeum vulgare*), cultivar Iron, and oat (*Avena sativa*) cultivar Belinda were grown in a climate chamber under the following conditions: Two weeks at 10°C/8°C 17 h/7 h 70%RH/60%RH, two weeks 15°C/12°C 18 h/6 h 70%RH/60%RH, three weeks 18°C/15°C 18 h/6 h 70%RH/60%RH and three weeks 20°C/15°C 17 h/6 h 70%RH/60%RH. During anthesis, approximately 1 mL of conidial solution was sprayed on each panicle, a bag was placed over the panicle and removed after 4 days. We used 6 plants per pot, 2 panicles per plant and 3 replicate pots per treatment. At sampling, panicles were immediately stored at −80°C.

### Transcriptomics of Fa05001 on barley heads

Panicles from one pot grown in climate chamber experiment were mixed and ground in liquid nitrogen. RNA was extracted from 50 mg subsample from three biological replicates (pots) of untreated control (0 dpi) and *F. avenaceum-*inoculated tissue (14 dpi), using Spectrum plant total RNA kit (Sigma-Aldrich, Steinheim, Germany), with slight modifications. Due to the high amount of starch in barley heads at 14 dpi, the volume of lysis buffer and binding solution were increased from 500 µL to 750 µL per sample, and samples were incubated for 5 min at room temperature and the lysates were filtered 2 times for 10 minutes. On-column DNase digestion (Sigma-Aldrich, Steinheim, Germany) was used.

PolyA purification and fragmentation, cDNA synthesis, library preparation and 1×100 bp single read module (half a lane Hi-seq 2500) sequencing were done by Eurofins MGW. The resulting fastaq files were trimmed (quality score limit: 0.05, maximum number of ambiguities: 2), and RNA-seq was performed with predicted *F. avenaceum* genes using CLC Genomics Workbench 6.05, with stringent settings (minimum similarity fraction: 0.95, minimum length fraction: 0.9, maximum number of hits for a read: 10) to subtract host-specific transcripts. Gene expression was calculated using reads per kilobase per million (RPKM) values. A T-test was used to determine significant expression levels in the biological replicates, comparing *F. avenaceum* inoculated samples against a control. Transcripts found solely in the *F. avenaceum-*inoculated plant were used to limit the amount of false positives coming from the host.

## Supporting Information

File S1 **Supplementary figures and tables.**
(DOCX)

File S2 **BLASTn results of the three** ***F. avenaceum*** **isolates Fa05001, FaLH03 and FaLH27.**
(XLSX)

File S3 **Venn diagram of BLASTn results corresponding to Figure S4.**
(XLSX)

File S4 **Interpro results of** ***F. avenaceum*** **Fa5001,** ***F. graminearum*** **,** ***F. verticillioides, F. oxysporum*** **and** ***F. solani*** **.**
(XLSX)

File S5 **Reciprocal blast of** ***F. avenaceum*** **Fa5001 vs** ***F. graminearum, F. verticillioides, F. oxysporum*** **and** ***F. solani*** **.**
(XLSX)

File S6 **Secretome of** ***F. avenaceum*** **Fa5001.**
(XLSX)

File S7 **Cysteine rich proteins in** ***F. avenaceum*** **Fa5001.**
(TXT)

File S8 **Summary of secondary metabolite genes.**
(XLSX)

File S9 **Transcriptome of** ***F. avenaceum*** **Fa5001 on barley heads.**
(XLSX)
