# Supplementary material for: The Genome of the Generalist Plant Pathogen Fusarium avenaceum Is Enriched with Genes Involved in Redox, Signaling and Secondary Metabolism
Source: PLoS One. 2014 Nov 19;9(11):e112703. doi: 10.1371/journal.pone.0112703 (PMC4237347; doi:10.1371/journal.pone.0112703)
Supplement: File S1 — Supplementary figures and tables. (DOCX) [file pone.0112703.s001.docx]

**Supplementary material for:**

**The genome of the generalist plant pathogenic fungus *Fusarium avenaceum* is enriched with genes involved in redox, signaling and secondary metabolism**

Figure S1. Electrokarytyping of Fa05001 chromosomes

Figure S2. Supercontigs of the three *F. avenaceum* isolates Fa05001, FaLH03 and FaLH27 with the scaffold numbers. The size of the supercontigs is based on FaLH03

Figure S3. Synteny plot of the three *F. avenaceum* isolates Fa05001, FaLH03 and FaLH27

Figure S4. Blast comparison of Fa05001, FALH03 and FALH27

Figure S5. Synteny plot of FaLH27 supercontigs vs *F. verticillioides* and *F. graminearum* chromosomes

Figure S6. Enriched biological processes of Fa05001 proteins with no ortholog (with expect more than 1e-10) in other sequenced Fusaria

Figure S7. Secretome of Fa05001, fisher’s exact test (FDR < 0.05)

Figure S8. Enriched GO categories in Fa05001 transcriptome on barley

Table S1. List of repetitive sequences in Fa05001 and *F. graminearum*

Table S2: Transcription factors. Isolate: Fa05001

Table S3: Functional analysis, GO category Biological Process. Isolate: Fa05001

Table S4: Functional analysis, GO category Molecular Function. Isolate: Fa05001

Table S5: Functional analysis, GO category Cellular Components. Isolate: Fa05001

Table S6. Secretome of Fa05001, fisher’s exact test (FDR < 0.05)

Table S7: Putative small cysteine rich proteins in Fa05001

Table S8: The apicidin-like gene cluster in the three *F. avenaceum* strains Fa05001, FaLH03 and FaLH27

Table S9: Protein similarity between gibberellic acid biosynthetic enzymes from *F. avenaceum* 05001, FaLH03 and FaLH27, *F. fujikuroi* MP-A, *F. proliferatum* KGL0401 and *S. manihoticola*

Table S10: Expression of Fa05001 genes in barley 14 dpi, FDR < 0.05

Table S11. Putative pathogenicity factors found in the transcriptome on barley

Table S12: Carbohydrate-Active enZYmes (CAZy). Isolate: Fa05001

Figure S1. Electrokaryotyping of Fa05001 chromosomes


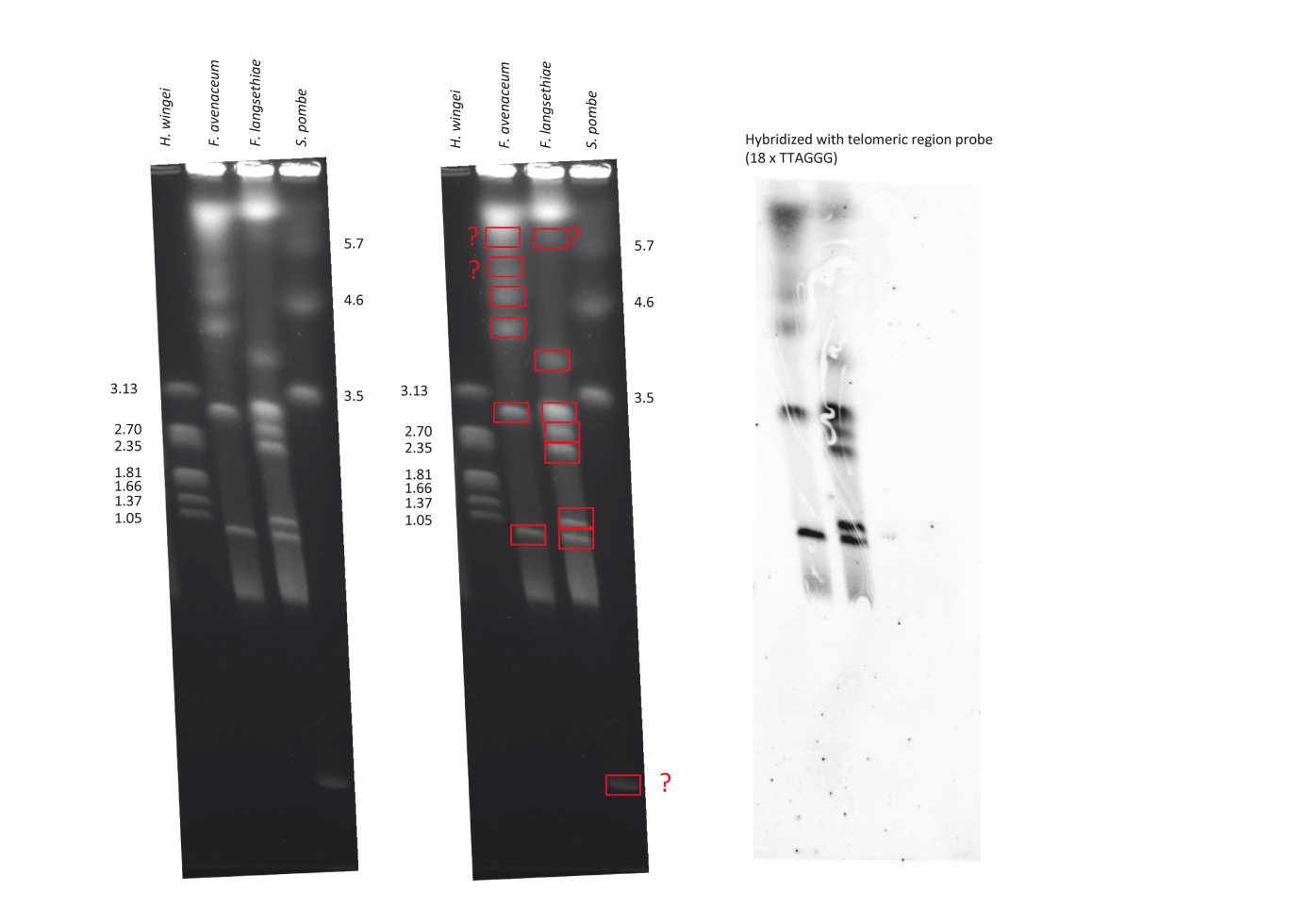


Figure S2. Supercontigs of the three *F. avenaceum* isolates Fa05001, FaLH03 and FaLH27 with the scaffold numbers. The size of the supercontigs is based on FaLH03.


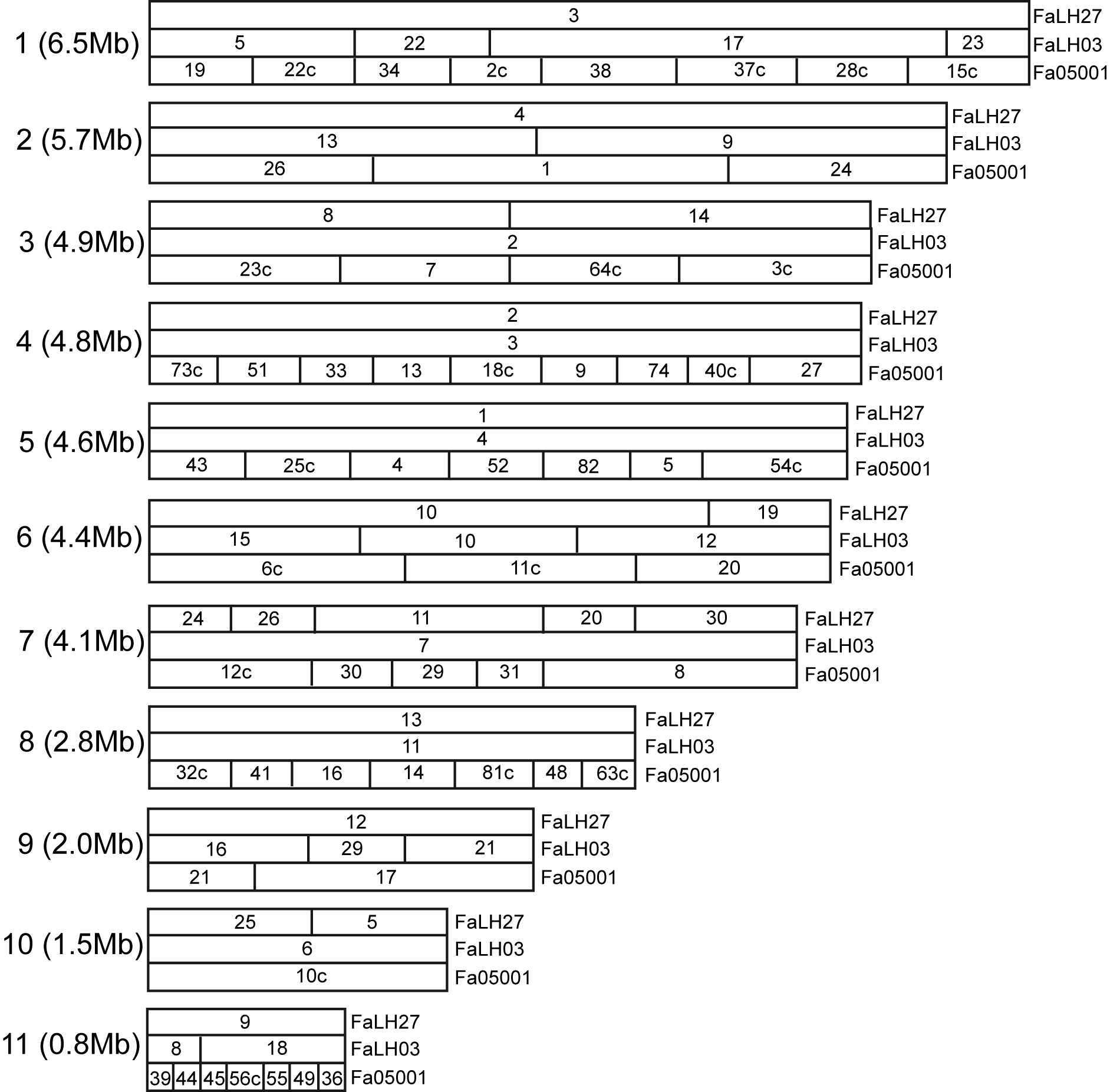


Figure S3. Synteny plot of the three *F. avenaceum* isolates Fa05001, FaLH03 and FaLH27


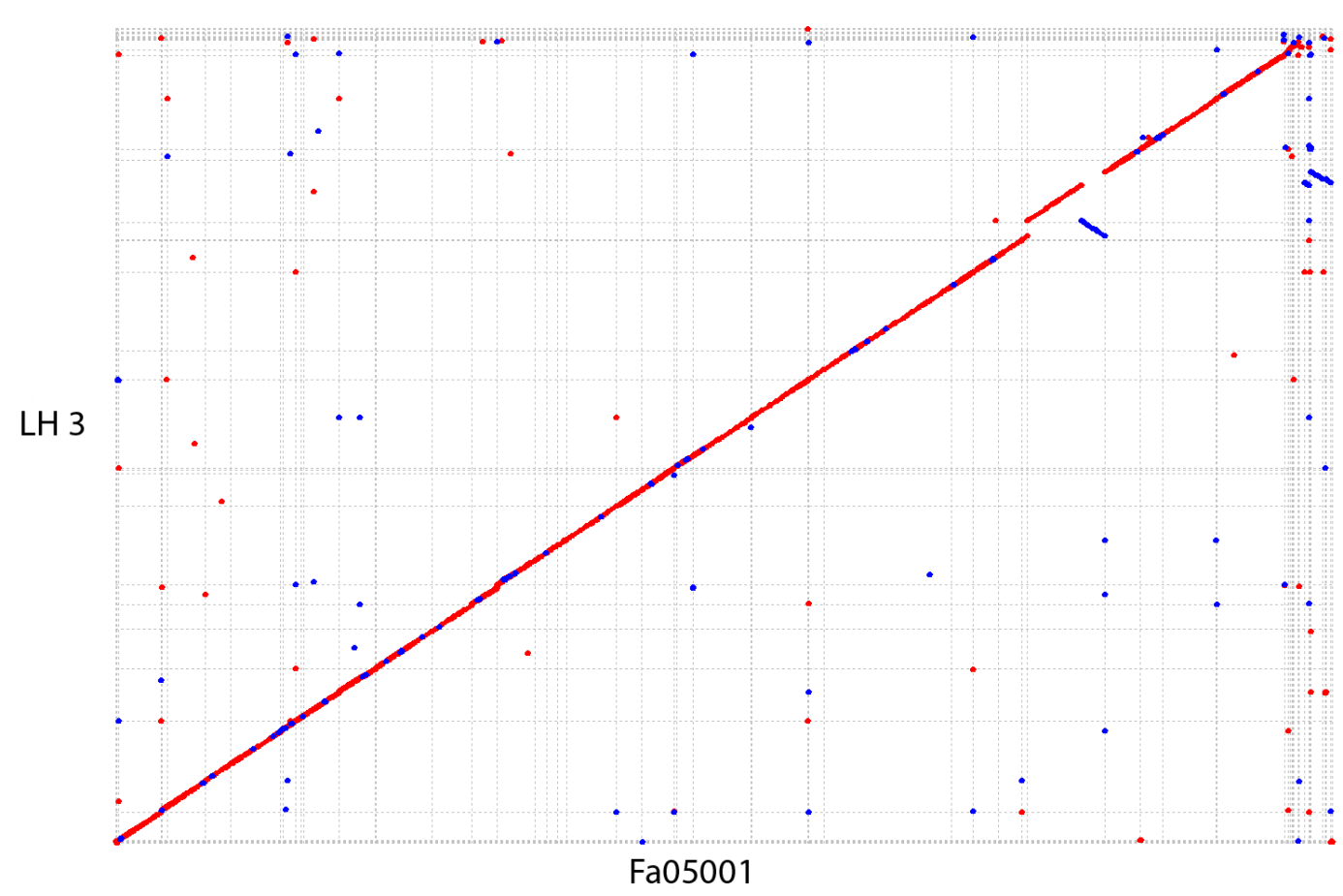


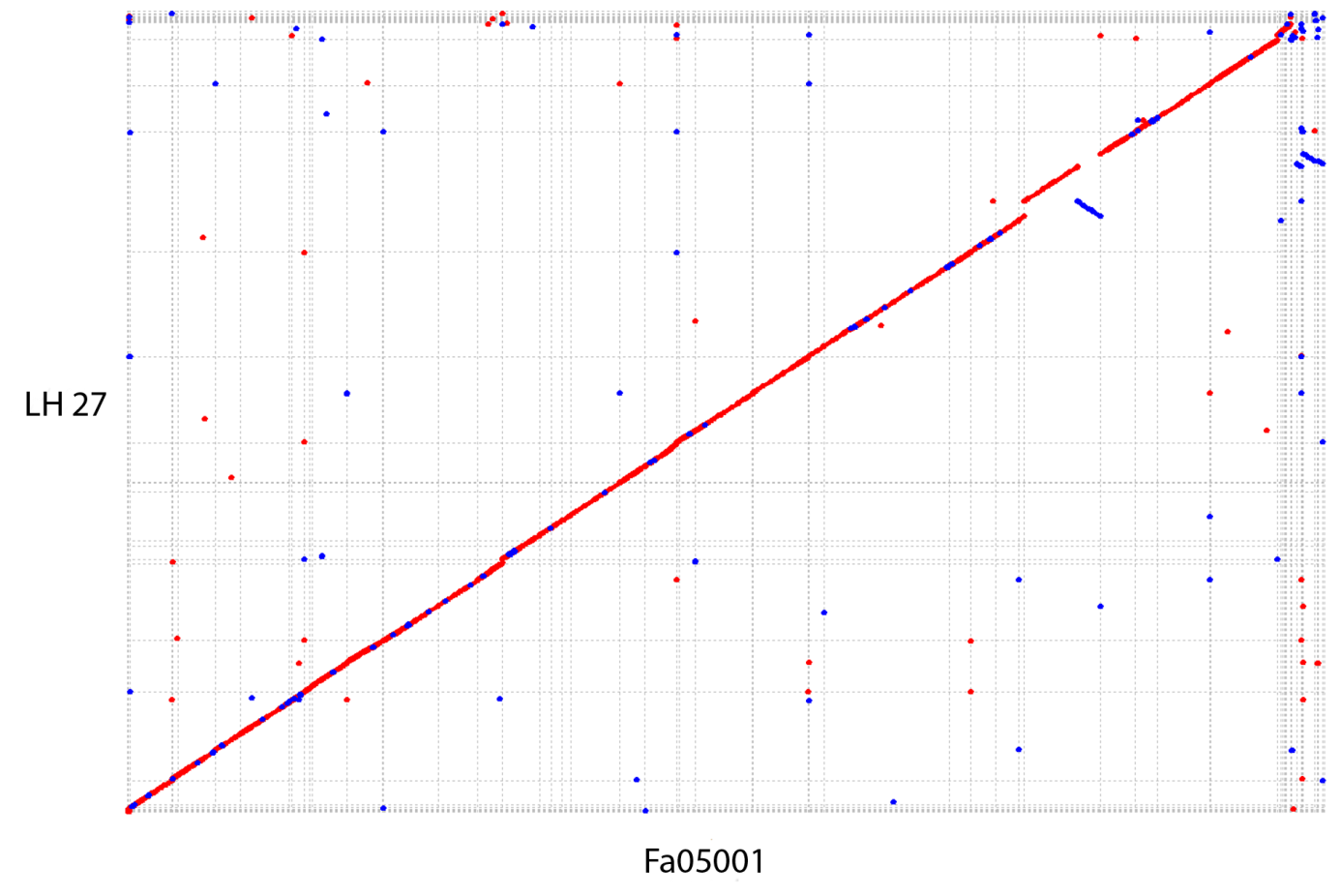


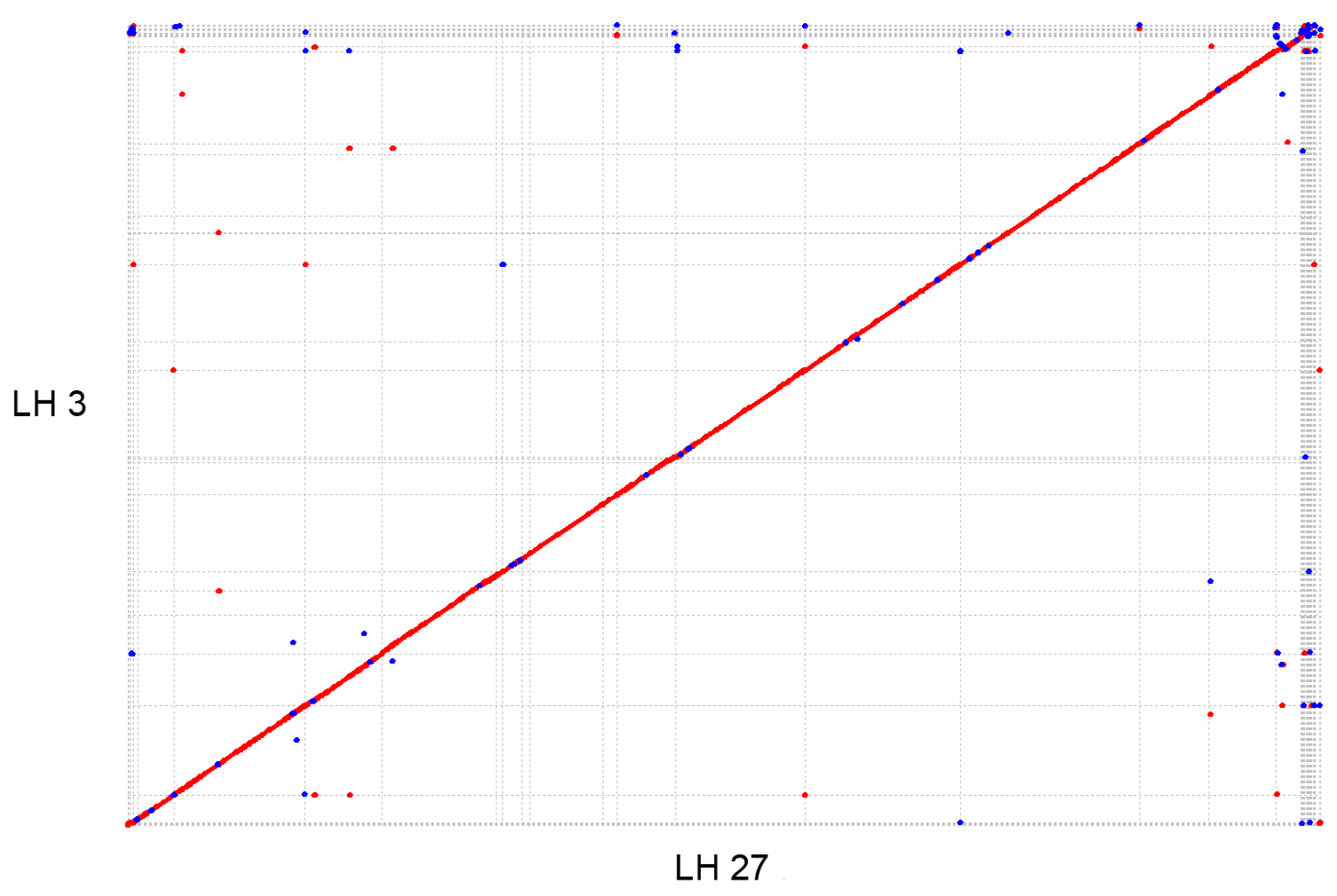


Figure S4. Blast comparison of Fa05001, FALH03 and FALH27. The corresponding lists are found in Supplementary file 2.


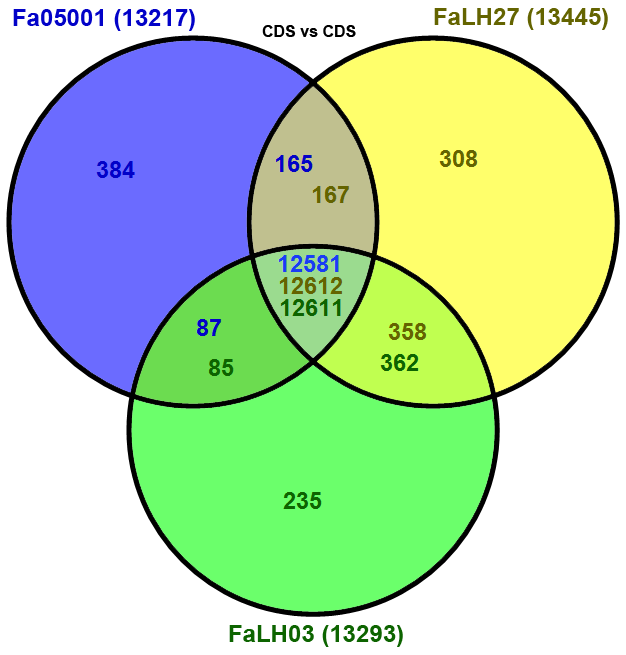

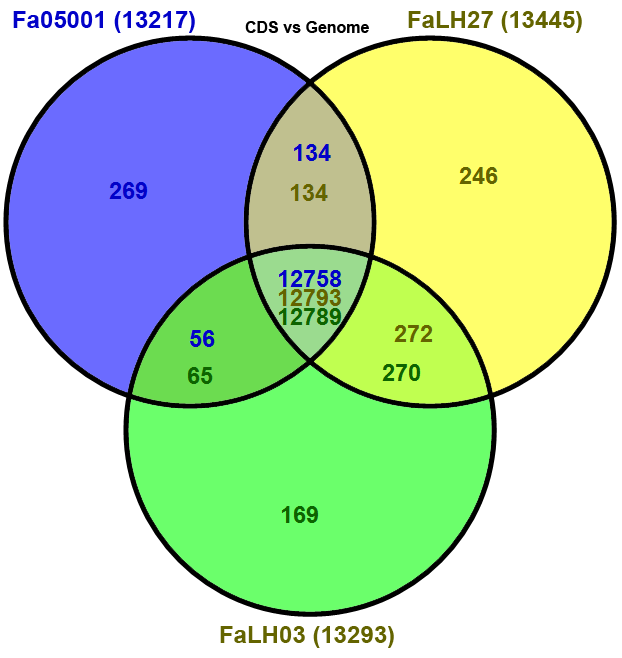


Figure S5. Synteny plot of FaLH27 supercontigs vs *F. verticillioides* and *F. graminearum* chromosomes.
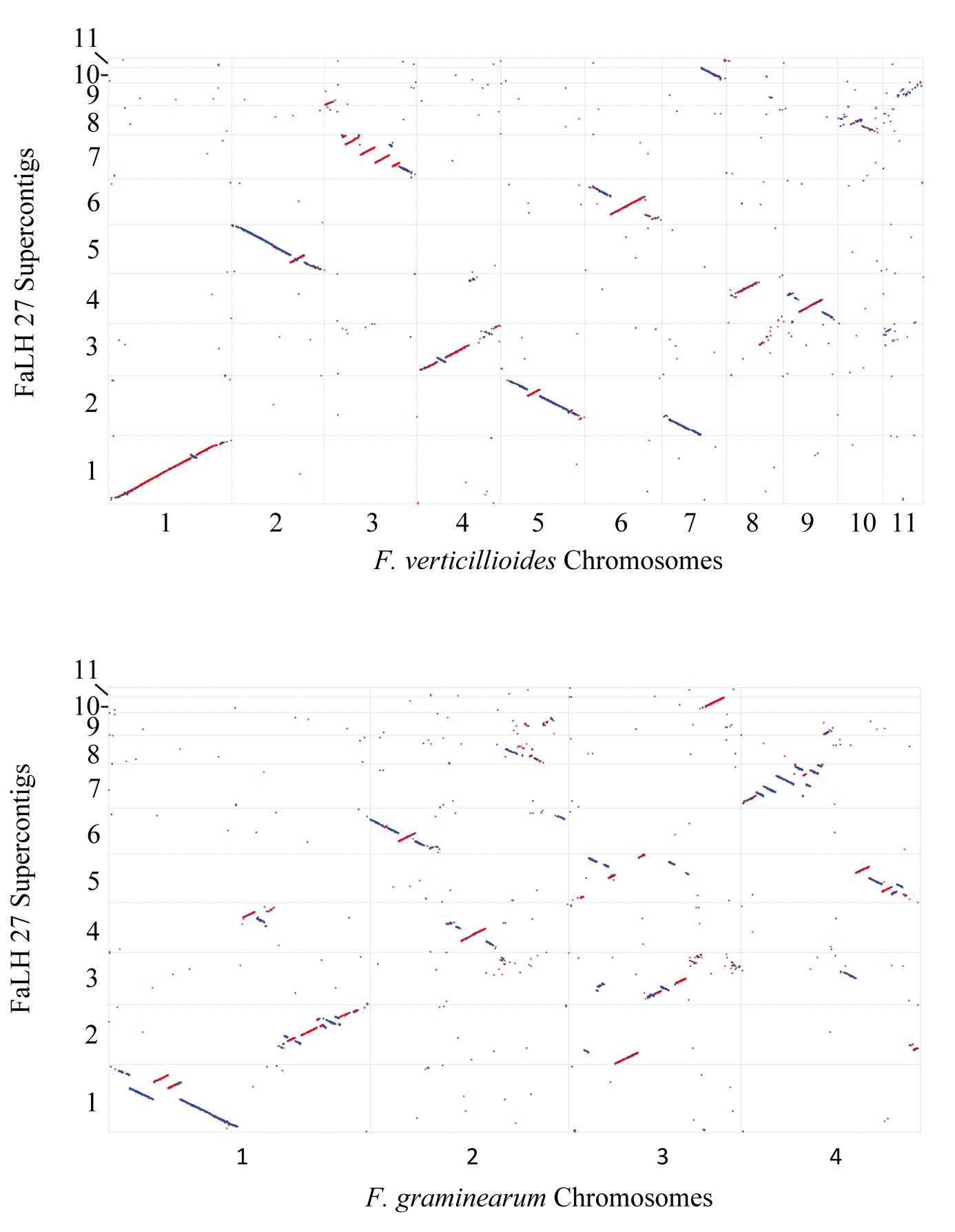


Figure S6. Enriched biological processes of Fa05001 proteins with no ortholog (with expect > 1e-10) in other sequenced Fusaria


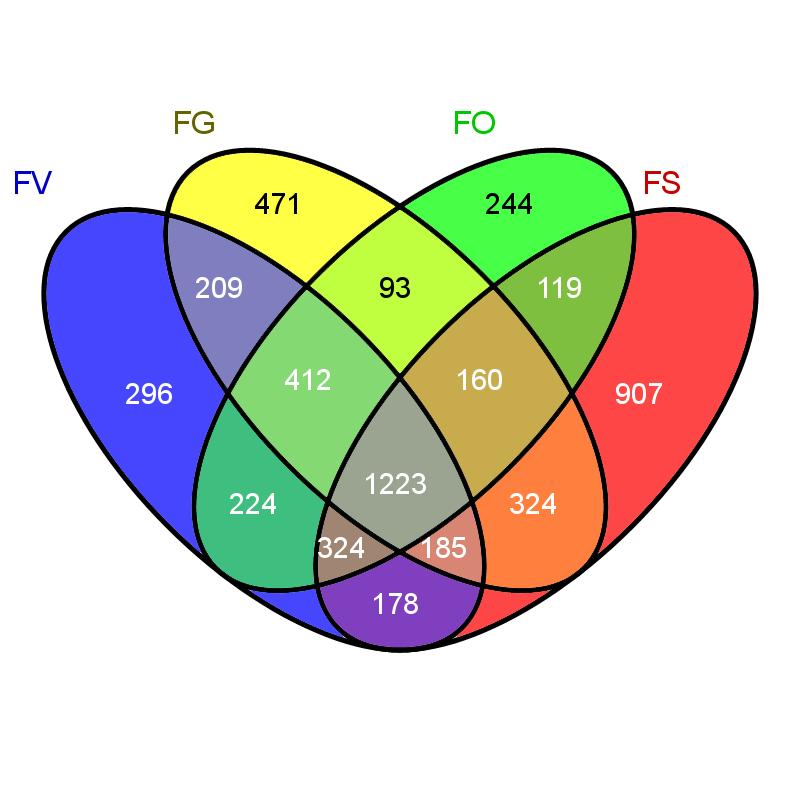

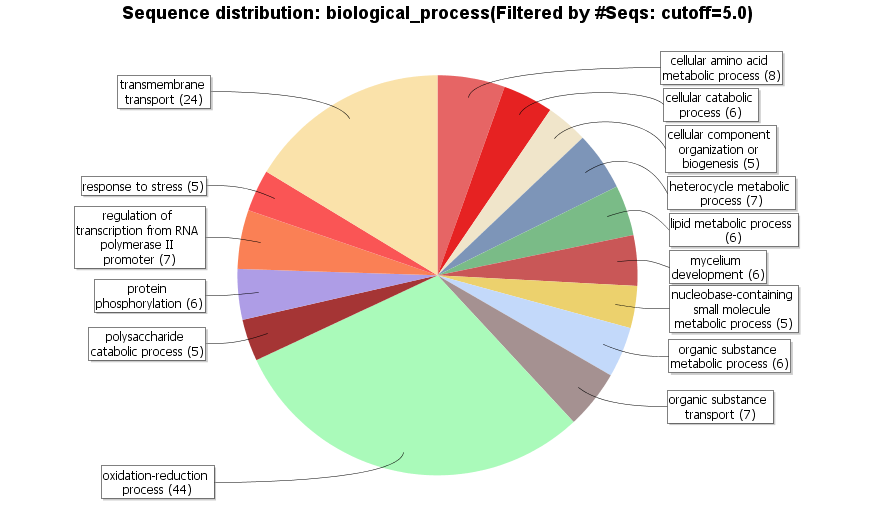


Figure S7. Secretome of Fa05001, fisher’s exact test (FDR < 0.05). Reference is whole genome


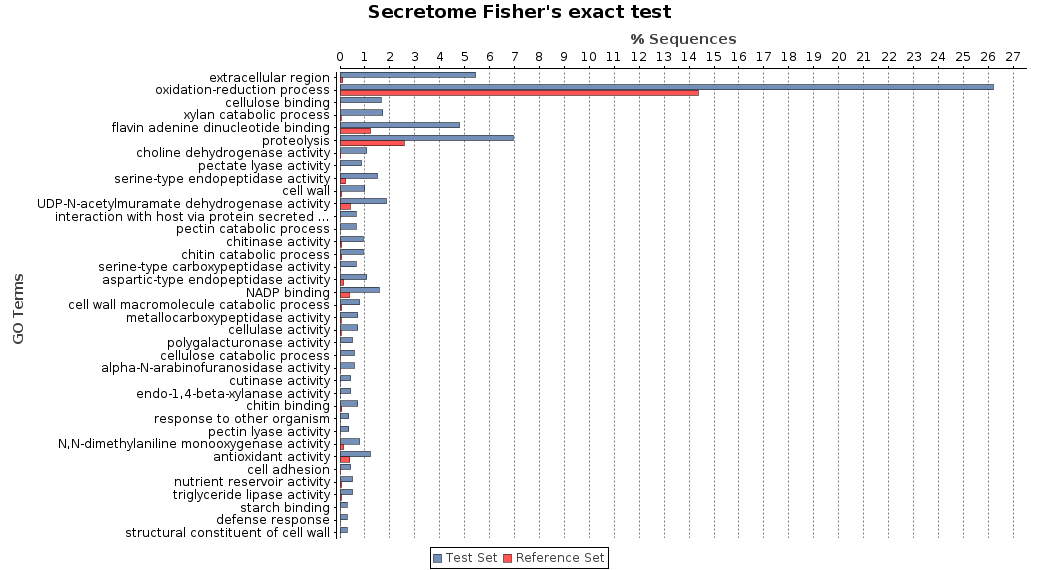


Figure S8. Enriched GO categories (Fishers exact test, P<0.05) in Fa05001 transcriptome on barley, where test set is transcriptome and reference is genome


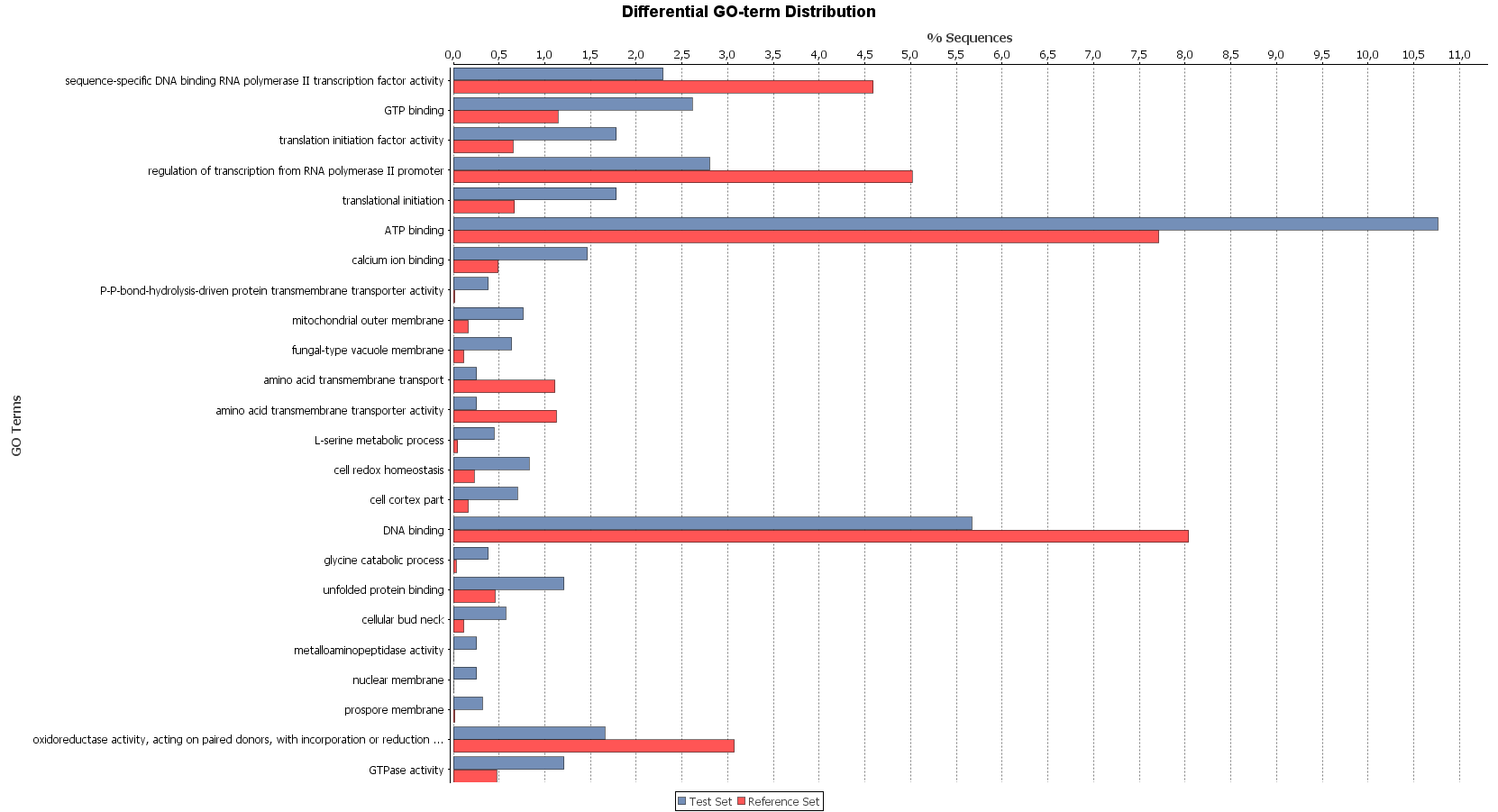


|  | | | ***Table S1: List of repetitive elements in genomes*** | | | | | |
| --- | --- | --- | --- | --- | --- | --- | --- | --- |
|  | | | ***F. avenaceum* Fa05001** | | | ***F. graminearum*** | | |
|  |  |  | Number of  elements | bp  included | Percentage in  genome | Number of  elements | bp  included | Percentage in  genome |
| Retroelements | LINE | Tad1 | 14 | 2718bp | 0.01% | 6 | 400bp | 0.00% |
|  | LTR elements | Copia | 57 | 3953bp | 0.01% | 47 | 4006bp | 0.01% |
|  |  | DIRS | 1 | 64bp | 0.00% | 1 | 67bp | 0.00% |
|  |  | Gypsy | 115 | 11405bp | 0.03% | 91 | 9763bp | 0.03% |
|  |  | TOTAL | 187 | 18140bp | 0.05% | 145 | 14236bp | 0.04% |
| DNA  Transposons | CMC-EnSpm | | 10 | 584bp | 0.00% | 3 | 125bp | 0.00% |
|  | hAT-Restless | | 4 | 663bp | 0.00% | 2 | 237bp | 0.00% |
|  | MULE-MuDR | | 11 | 8168bp | 0.02% | 3 | 553bp | 0.00% |
|  | PIF-Harbinger | | 8 | 622bp | 0.00% | 10 | 825bp | 0.00% |
|  | PiggyBac | | 8 | 1386bp | 0.00% | 1 | 1945bp | 0.00% |
|  | TcMar-Ant1 | | 2 | 183bp | 0.00% | 10 | 3256bp | 0.01% |
|  | TcMar-Fot1 | | 35 | 16876bp | 0.04% | 17 | 12197bp | 0.03% |
|  | TcMar-Pogo | | 9 | 2642bp | 0.01% | 9 | 2584bp | 0.01% |
|  | TOTAL | | 106 | 33552bp | 0.08% | 59 | 22015bp | 0.06% |
| Unclassified | | | 3 | 178bp | 0.00% | 9 | 798bp | 0.00% |
| Total interspersed repeats | | |  | 52043bp | 0.13% |  | 36727bp | 0.10% |
| Small RNA | | | 86 | 15222bp | 0.04% | 119 | 78226bp | 0.21% |
| Simple repeats | | | 6086 | 260626bp | 0.63% | 5532 | 225914bp | 0.62% |
| Low complexity | | | 698 | 34037bp | 0.08% | 645 | 30217bp | 0.08% |
| **TOTAL** | | | **7166** | **413798bp** | **1.00%** | **6509** | **408133bp** | **1.12%** |

| ***Table S2: Transcription factors*** | | | | | | |
| --- | --- | --- | --- | --- | --- | --- |
| Interpro | Type | FA | FG | FO | FV | FS |
| IPR001138 | Fungal transcriptional regulatory protein, N-terminal | 310 | 264 | 303 | 236 | 519 |
| IPR007219 | Fungal specific transcription factor | 301 | 196 | 252 | 199 | 391 |
| IPR007087 | Zinc finger, C2H2-type | 94 | 90 | 105 | 82 | 124 |
| IPR011991 | Winged helix repressor DNA-binding | 61 | 54 | 66 | 52 | 56 |
| IPR012340 | Nucleic acid-binding, OB-fold | 51 | 48 | 47 | 44 | 48 |
| IPR009057 | Homeodomain-like | 49 | 41 | 50 | 41 | 55 |
| IPR004827 | Basic-leucine zipper (bZIP) transcription factor | 30 | 28 | 90 | 25 | 33 |
| IPR001005 | Myb, DNA-binding | 17 | 17 | 22 | 14 | 20 |
| IPR001356 | Homeobox | 15 | 12 | 12 | 9 | 21 |
| IPR001878 | Zinc finger, CCHC-type | 10 | 14 | 27 | 11 | 15 |
| IPR009071 | High mobility group box | 9 | 10 | 12 | 8 | 9 |
| IPR003958 | Transcription factor CBF/NF-Y/archaeal histone | 7 | 7 | 7 | 5 | 5 |
| IPR001594 | Zinc finger, DHHC-type | 6 | 5 | 5 | 3 | 6 |
| IPR000679 | Zinc finger, GATA-type | 5 | 7 | 8 | 6 | 8 |
| IPR003163 | DNA-binding, yeast (APSES) | 5 | 5 | 5 | 4 | 5 |
| IPR006600 | Centromere protein B, DNA-binding region | 5 | 6 | 17 | 3 | 8 |
| IPR001606 | AT-rich interaction region | 4 | 3 | 4 | 3 | 3 |
| IPR001766 | Fork head transcription factor | 4 | 4 | 5 | 4 | 4 |
| IPR001222 | Transcription factor TFIIS | 3 | 2 | 2 | 1 | 4 |
| IPR004181 | Zinc finger, MIZ-type | 3 | 3 | 2 | 2 | 2 |
| IPR006565 | Bromodomain transcription factor | 3 | 3 | 3 | 3 | 3 |
| IPR006642 | Zinc finger, Rad18-type putative | 3 |  |  | 3 |  |
| IPR007396 | Negative transcriptional regulator | 3 | 2 | 2 | 3 | 2 |
| IPR007889 | Helix-turn-helix, Psq | 3 | 1 | 2 | 1 | 1 |
| IPR008967 | p53-like transcription factor, DNA-binding | 3 | 3 | 4 | 3 | 3 |
| IPR002100 | Transcription factor, MADS-box | 2 | 2 | 4 | 2 | 2 |
| IPR003150 | DNA-binding RFX | 2 | 1 | 2 | 2 | 2 |
| IPR010666 | Zinc finger, GRF-type | 2 | 3 | 2 | 1 | 1 |
| IPR010982 | Lambda repressor-like, DNA-binding | 2 | 2 | 5 | 2 | 2 |
| IPR000116 | High mobility group proteins HMG-I and HMG-Y | 1 | 1 | 1 |  | 1 |
| IPR000818 | TEA/ATTS | 1 | 1 | 1 | 1 | 1 |
| IPR000967 | Zinc finger, NF-X1-type | 1 | 1 |  |  | 1 |
| IPR001289 | CCAAT-binding transcription factor, subunit B | 1 | 1 | 1 | 1 | 1 |
| IPR001387 | Helix-turn-helix type 3 | 1 | 1 | 1 | 1 | 1 |
| IPR001510 | Zinc finger, PARP-type | 1 | 1 | 1 | 1 | 1 |
| IPR002059 | Cold-shock protein, DNA-binding | 1 | 1 |  |  |  |
| IPR003120 | Transcription factor, STE-like | 1 |  | 1 | 1 | 1 |
| IPR003656 | Zinc finger, BED-type predicted | 1 | 1 | 1 | 1 | 1 |
| IPR003956 | Histone-like transcription factor CBF/NF-Y/archaeal histone, subunit A | 1 | 1 |  |  | 1 |
| IPR004022 | DDT | 1 | 1 | 1 | 1 | 1 |
| IPR005011 | SART-1 protein | 1 | 1 | 1 | 1 | 1 |
| IPR006856 | Mating-type protein MAT alpha 1 | 1 | 1 | 1 | 1 | 1 |
| IPR007196 | CCR4-Not complex component, Not1 | 1 | 1 |  | 1 | 1 |
| IPR007604 | CP2 transcription factor | 1 | 1 | 1 | 1 | 1 |
| IPR008895 | YL1 nuclear | 1 | 1 | 1 | 1 | 1 |
| IPR009044 | ssDNA-binding transcriptional regulator | 1 | 1 | 1 | 1 | 1 |
| IPR009395 | GCN5-like 1 | 1 | 1 | 1 | 1 | 1 |
| IPR010770 | SGT1 | 1 | 1 | 1 | 1 | 1 |
| IPR000637 | HMG-I/HMG-Y, DNA-binding, conserved site |  | 1 | 1 |  | 1 |

| ***Table S3: Functional analysis, GO category Biological Process*** | | | | | |
| --- | --- | --- | --- | --- | --- |
| GO category | FA | FG | FO | FV | FS |
| transmembrane transport | 623 | 515 | 866 | 671 | 823 |
| proteolysis | 198 | 198 | 285 | 202 | 250 |
| methylation | 101 | 91 | 130 | 89 | 112 |
| vesicle-mediated transport | 41 | 43 | 47 | 44 | 39 |
| intracellular signal transduction | 38 | 37 | 60 | 39 | 34 |
| phosphorelay signal transduction system | 26 | 27 | 38 | 30 | 20 |
| signal transduction by phosphorylation | 22 | 22 | 33 | 26 | 15 |
| cellular response to oxidative stress | 20 | 16 | 18 | 18 | 16 |
| glycolysis | 16 | 16 | 26 | 16 | 14 |
| negative regulation of catalytic activity | 16 | 15 | 17 | 15 | 13 |
| protein catabolic process | 14 | 14 | 14 | 13 | 13 |
| glycerol ether metabolic process | 13 | 12 | 16 | 11 | 11 |
| cell wall macromolecule catabolic process | 12 | 7 | 14 | 15 | 25 |
| pentose-phosphate shunt | 12 | 8 | 18 | 13 | 18 |
| cellular nitrogen compound biosynthetic process | 11 | 7 | 11 | 10 | 14 |
| autophagy | 9 | 7 | 8 | 11 | 8 |
| vesicle docking involved in exocytosis | 8 | 10 | 10 | 8 | 8 |
| ion transport | 8 | 6 | 13 | 7 | 7 |
| apoptotic process | 7 | 5 | 7 | 7 | 9 |
| phospholipid catabolic process | 6 | 9 | 17 | 7 | 7 |
| aromatic compound catabolic process | 6 | 3 | 9 | 8 | 10 |
| superoxide metabolic process | 6 | 6 | 7 | 6 | 6 |
| sterol metabolic process | 6 | 6 | 7 | 6 | 4 |
| purine nucleobase biosynthetic process | 6 | 5 | 6 | 5 | 4 |
| vacuole fusion, non-autophagic | 5 | 4 | 4 | 3 | 5 |
| tetrapyrrole biosynthetic process | 4 | 4 | 6 | 4 | 7 |
| aromatic amino acid family metabolic process | 4 | 5 | 5 | 3 | 6 |
| ascospore wall assembly | 4 | 3 | 5 | 4 | 5 |
| sulfate assimilation | 4 | 4 | 4 | 4 | 4 |
| cell wall modification | 4 | 3 | 5 | 3 | 4 |
| nitrate assimilation | 4 | 3 | 4 | 4 | 3 |
| cellular amino acid biosynthetic process | 4 | 2 | 3 | 5 | 4 |
| glutathione metabolic process | 4 | 3 | 3 | 4 | 3 |
| branched-chain amino acid metabolic process | 4 | 1 | 2 | 1 | 2 |
| cellular amino acid catabolic process | 3 | 2 | 8 | 4 | 6 |
| acyl-CoA metabolic process | 3 | 3 | 4 | 4 | 5 |
| regulation of signal transduction | 3 | 4 | 4 | 3 | 3 |
| peptidyl-diphthamide biosynthetic process from peptidyl-histidine | 3 | 4 | 3 | 2 | 4 |
| intracellular transport | 3 | 4 | 4 | 3 | 2 |
| cellular bud site selection | 3 | 3 | 3 | 3 | 3 |
| cellular protein metabolic process | 3 | 2 | 3 | 2 | 5 |
| phosphate-containing compound metabolic process | 3 | 1 | 3 | 2 | 2 |
| toxin biosynthetic process | 3 | 0 | 0 | 0 | 0 |
| response to DNA damage stimulus | 2 | 5 | 5 | 4 | 5 |
| neurotransmitter transport | 2 | 3 | 4 | 5 | 2 |
| ketone body catabolic process | 2 | 2 | 4 | 5 | 3 |
| cellular ion homeostasis | 2 | 3 | 3 | 3 | 3 |
| cellular response to heat | 2 | 3 | 3 | 2 | 2 |
| urea catabolic process | 2 | 2 | 2 | 2 | 3 |
| oligosaccharide metabolic process | 2 | 2 | 2 | 2 | 3 |
| cell surface receptor signaling pathway | 2 | 3 | 2 | 1 | 3 |
| establishment or maintenance of actin cytoskeleton polarity | 2 | 2 | 2 | 2 | 2 |
| lipid transport | 2 | 2 | 2 | 2 | 2 |
| response to unfolded protein | 2 | 2 | 2 | 2 | 2 |
| regulation of cell size | 2 | 2 | 2 | 1 | 3 |
| cellular response to starvation | 2 | 2 | 2 | 2 | 2 |
| signal transduction involved in filamentous growth | 2 | 2 | 1 | 2 | 2 |
| D-ribose metabolic process | 2 | 1 | 2 | 2 | 2 |
| regulation of cell cycle | 2 | 1 | 2 | 2 | 2 |
| furaldehyde metabolic process | 2 | 2 | 2 | 1 | 2 |
| defense response to bacterium | 2 | 2 | 2 | 1 | 1 |
| defense response to fungus | 2 | 2 | 2 | 1 | 1 |
| vesicle fusion | 2 | 1 | 1 | 1 | 2 |
| ornithine metabolic process | 2 | 2 | 1 | 1 | 1 |
| response to salt stress | 2 | 1 | 1 | 1 | 1 |
| chlorophyll biosynthetic process | 1 | 1 | 4 | 3 | 7 |
| phenol-containing compound metabolic process | 1 | 2 | 1 | 1 | 5 |
| nucleic acid metabolic process | 1 | 2 | 1 | 2 | 4 |
| cyanate metabolic process | 1 | 1 | 4 | 1 | 1 |
| nucleotide-sugar metabolic process | 1 | 1 | 2 | 2 | 2 |
| positive regulation of catalytic activity | 1 | 2 | 1 | 1 | 1 |
| recognition of pollen | 1 | 1 | 2 | 1 | 1 |
| response to light stimulus | 1 | 1 | 2 | 1 | 1 |
| lipid storage | 1 | 1 | 1 | 1 | 1 |
| organic acid phosphorylation | 1 | 1 | 1 | 1 | 1 |
| karyogamy involved in conjugation with cellular fusion | 1 | 1 | 1 | 1 | 1 |
| mitochondria-nucleus signaling pathway | 1 | 1 | 1 | 1 | 1 |
| polyol metabolic process | 1 | 1 | 1 | 1 | 1 |
| cell-matrix adhesion | 1 | 1 | 1 | 1 | 1 |
| mitochondrial fusion | 1 | 1 | 1 | 1 | 1 |
| removal of nonhomologous ends | 1 | 1 | 1 | 1 | 1 |
| actomyosin contractile ring contraction | 1 | 0 | 2 | 1 | 1 |
| peptide catabolic process | 1 | 1 | 1 | 1 | 1 |
| regulation of cell growth | 1 | 1 | 1 | 1 | 1 |
| glycerol-3-phosphate catabolic process | 1 | 1 | 1 | 1 | 1 |
| steroid metabolic process | 1 | 1 | 1 | 1 | 1 |
| microtubule nucleation | 1 | 1 | 1 | 1 | 1 |
| cell cycle arrest | 1 | 1 | 1 | 2 | 0 |
| vesicle transport along actin filament | 1 | 1 | 1 | 1 | 1 |
| hydrogen peroxide metabolic process | 1 | 1 | 1 | 1 | 1 |
| L-arabinose catabolic process to xylulose 5-phosphate | 1 | 1 | 1 | 1 | 1 |
| heme biosynthetic process | 1 | 1 | 1 | 1 | 0 |
| intracellular mRNA localization | 1 | 1 | 1 | 1 | 0 |
| actin filament organization | 1 | 1 | 1 | 1 | 0 |
| cell wall biogenesis | 1 | 1 | 1 | 0 | 1 |
| regulation of primary metabolic process | 1 | 1 | 2 | 0 | 0 |
| response to metal ion | 1 | 1 | 1 | 0 | 1 |
| detoxification of arsenic-containing substance | 1 | 1 | 1 | 0 | 1 |
| regulation of cellular metabolic process | 1 | 0 | 2 | 0 | 0 |
| detoxification of cadmium ion | 1 | 0 | 1 | 0 | 1 |
| regulation of macromolecule metabolic process | 1 | 0 | 2 | 0 | 0 |
| glutamine family amino acid metabolic process | 1 | 1 | 0 | 0 | 0 |
| seed germination | 1 | 0 | 0 | 0 | 0 |
| carotenoid biosynthetic process | 0 | 0 | 3 | 2 | 6 |
| cellular macromolecule biosynthetic process | 0 | 1 | 1 | 0 | 1 |
| alcohol biosynthetic process | 0 | 0 | 3 | 0 | 0 |
| cellular response to drug | 0 | 0 | 0 | 1 | 1 |
| cellular protein localization | 0 | 2 | 0 | 0 | 0 |
| positive regulation of cellular process | 0 | 1 | 0 | 0 | 1 |
| Sertoli cell development | 0 | 0 | 1 | 0 | 1 |
| phytoalexin catabolic process | 0 | 0 | 0 | 0 | 2 |
| capsule organization | 0 | 0 | 0 | 1 | 0 |
| response to ethylene stimulus | 0 | 1 | 0 | 0 | 0 |
| cellular response to organic substance | 0 | 1 | 0 | 0 | 0 |
| regulation of cell communication | 0 | 1 | 0 | 0 | 0 |
| body morphogenesis | 0 | 1 | 0 | 0 | 0 |
| autophagic cell death | 0 | 1 | 0 | 0 | 0 |
| establishment of protein localization | 0 | 1 | 0 | 0 | 0 |
| positive regulation of transport | 0 | 1 | 0 | 0 | 0 |
| response to auxin stimulus | 0 | 1 | 0 | 0 | 0 |
| nervous system development | 0 | 0 | 1 | 0 | 0 |
| organ development | 0 | 0 | 1 | 0 | 0 |
| response to pheromone involved in conjugation with cellular fusion | 0 | 0 | 1 | 0 | 0 |
| single fertilization | 0 | 0 | 1 | 0 | 0 |
| leaf morphogenesis | 0 | 0 | 1 | 0 | 0 |
| cell cycle phase | 0 | 0 | 0 | 0 | 1 |
| nitrogen compound transport | 0 | 0 | 0 | 0 | 1 |
| cell projection organization | 0 | 0 | 0 | 0 | 1 |
| egress of virus within host cell | 0 | 0 | 0 | 0 | 1 |
| organomercury catabolic process | 0 | 0 | 0 | 0 | 1 |

| ***Table S4: Functional analysis, GO category Molecular Function*** | | | | | |
| --- | --- | --- | --- | --- | --- |
| GO category | FA | FG | FO | FV | FS |
| metal ion binding | 244 | 184 | 269 | 217 | 212 |
| flavin adenine dinucleotide binding | 143 | 135 | 195 | 135 | 181 |
| hydrolase activity, hydrolyzing O-glycosyl compounds | 137 | 117 | 179 | 135 | 150 |
| methyltransferase activity | 78 | 72 | 102 | 66 | 95 |
| sequence-specific DNA binding | 68 | 72 | 164 | 73 | 82 |
| kinase activity | 57 | 56 | 60 | 45 | 50 |
| ATPase activity, coupled to transmembrane movement of substances | 46 | 43 | 65 | 55 | 58 |
| carbon-nitrogen ligase activity, with glutamine as amido-N-donor | 44 | 34 | 62 | 45 | 45 |
| oxidoreductase activity, acting on single donors with incorporation of molecular oxygen, incorporation of two atoms of oxygen | 36 | 28 | 45 | 34 | 50 |
| transferase activity, transferring hexosyl groups | 34 | 40 | 43 | 35 | 38 |
| oxidoreductase activity, acting on paired donors, with incorporation or reduction of molecular oxygen, 2-oxoglutarate as one donor, and incorporation of one atom each of oxygen into both donors | 33 | 21 | 26 | 24 | 29 |
| oxidoreductase activity, acting on the aldehyde or oxo group of donors, NAD or NADP as acceptor | 31 | 31 | 56 | 44 | 55 |
| transferase activity, transferring acyl groups other than amino-acyl groups | 29 | 33 | 37 | 42 | 38 |
| oxidoreductase activity, acting on the CH-OH group of donors, NAD or NADP as acceptor | 28 | 23 | 43 | 29 | 41 |
| transaminase activity | 26 | 29 | 33 | 26 | 27 |
| acid-amino acid ligase activity | 25 | 19 | 30 | 27 | 23 |
| actin binding | 25 | 22 | 25 | 22 | 23 |
| cellulose binding | 24 | 19 | 21 | 18 | 23 |
| carboxy-lyase activity | 23 | 21 | 36 | 21 | 25 |
| phosphotransferase activity, alcohol group as acceptor | 22 | 21 | 31 | 20 | 16 |
| hydrolase activity, acting on carbon-nitrogen (but not peptide) bonds, in linear amides | 22 | 14 | 29 | 23 | 20 |
| serine-type peptidase activity | 21 | 21 | 25 | 19 | 27 |
| phosphatidylinositol binding | 21 | 21 | 23 | 24 | 19 |
| 2 iron, 2 sulfur cluster binding | 20 | 18 | 25 | 24 | 23 |
| 4 iron, 4 sulfur cluster binding | 20 | 17 | 24 | 21 | 23 |
| peptidyl-prolyl cis-trans isomerase activity | 20 | 16 | 23 | 20 | 20 |
| damaged DNA binding | 19 | 21 | 22 | 17 | 18 |
| choline dehydrogenase activity | 17 | 16 | 26 | 19 | 29 |
| RNA polymerase II transcription cofactor activity | 17 | 13 | 19 | 19 | 14 |
| nucleotidyltransferase activity | 16 | 13 | 18 | 21 | 16 |
| NAD+ binding | 16 | 17 | 16 | 12 | 11 |
| chitin binding | 15 | 13 | 17 | 12 | 16 |
| acyl-CoA dehydrogenase activity | 14 | 11 | 24 | 12 | 17 |
| sulfuric ester hydrolase activity | 13 | 9 | 24 | 17 | 17 |
| mRNA binding | 11 | 10 | 11 | 10 | 10 |
| carboxylic ester hydrolase activity | 10 | 7 | 14 | 11 | 18 |
| tRNA binding | 10 | 9 | 11 | 9 | 8 |
| rRNA binding | 10 | 9 | 8 | 9 | 8 |
| nuclease activity | 8 | 10 | 9 | 8 | 11 |
| hydrolase activity, acting on carbon-nitrogen (but not peptide) bonds, in linear amidines | 8 | 6 | 11 | 9 | 7 |
| guanyl-nucleotide exchange factor activity | 8 | 9 | 7 | 7 | 10 |
| pseudouridine synthase activity | 8 | 7 | 9 | 8 | 6 |
| peroxiredoxin activity | 8 | 4 | 9 | 7 | 8 |
| cytochrome-c oxidase activity | 7 | 6 | 7 | 6 | 7 |
| glutathione transferase activity | 7 | 4 | 4 | 2 | 2 |
| oxidoreductase activity, acting on paired donors, with incorporation or reduction of molecular oxygen, NAD(P)H as one donor, and incorporation of two atoms of oxygen into one donor | 6 | 3 | 9 | 8 | 10 |
| phosphoric ester hydrolase activity | 5 | 5 | 7 | 6 | 5 |
| 1-aminocyclopropane-1-carboxylate synthase activity | 5 | 3 | 5 | 2 | 9 |
| ARF GTPase activator activity | 5 | 5 | 5 | 4 | 5 |
| ubiquitin protein ligase binding | 5 | 6 | 5 | 3 | 5 |
| protein homodimerization activity | 5 | 5 | 5 | 4 | 4 |
| 7S RNA binding | 5 | 4 | 5 | 3 | 5 |
| biotin carboxylase activity | 4 | 4 | 6 | 6 | 8 |
| phosphotransferase activity, for other substituted phosphate groups | 4 | 4 | 6 | 5 | 4 |
| proline dehydrogenase activity | 4 | 3 | 5 | 4 | 7 |
| hydrolase activity, acting on acid halide bonds, in C-halide compounds | 4 | 4 | 5 | 4 | 3 |
| ubiquitin binding | 4 | 4 | 4 | 3 | 4 |
| G-protein coupled receptor activity | 4 | 1 | 5 | 5 | 4 |
| hydroxymethyl-, formyl- and related transferase activity | 4 | 4 | 4 | 4 | 3 |
| starch binding | 4 | 3 | 4 | 4 | 3 |
| transcription coactivator activity | 4 | 2 | 4 | 4 | 4 |
| tRNA dihydrouridine synthase activity | 4 | 4 | 4 | 3 | 3 |
| prenyltransferase activity | 4 | 4 | 4 | 3 | 1 |
| tryptophan dimethylallyltransferase activity | 4 | 0 | 2 | 1 | 1 |
| aldehyde-lyase activity | 3 | 3 | 6 | 2 | 7 |
| oxo-acid-lyase activity | 3 | 3 | 4 | 4 | 3 |
| aspartyl esterase activity | 3 | 2 | 5 | 3 | 4 |
| 3-deoxy-7-phosphoheptulonate synthase activity | 3 | 3 | 4 | 4 | 3 |
| oxidoreductase activity, acting on paired donors, with oxidation of a pair of donors resulting in the reduction of molecular oxygen to two molecules of water | 3 | 3 | 3 | 3 | 3 |
| small GTPase regulator activity | 3 | 3 | 3 | 3 | 3 |
| adenylosuccinate synthase activity | 3 | 1 | 5 | 2 | 3 |
| racemase and epimerase activity, acting on carbohydrates and derivatives | 3 | 3 | 3 | 4 | 1 |
| calcium-dependent phospholipid binding | 3 | 2 | 3 | 3 | 3 |
| protein phosphatase type 2A regulator activity | 3 | 3 | 3 | 3 | 2 |
| snoRNA binding | 3 | 3 | 2 | 3 | 3 |
| acetolactate synthase activity | 3 | 2 | 3 | 2 | 3 |
| transketolase activity | 3 | 2 | 3 | 2 | 3 |
| snRNA binding | 3 | 2 | 1 | 3 | 2 |
| phosphoribosylamine-glycine ligase activity | 3 | 2 | 3 | 2 | 1 |
| oxidoreductase activity, acting on the CH-CH group of donors, NAD or NADP as acceptor | 2 | 3 | 6 | 3 | 2 |
| neurotransmitter:sodium symporter activity | 2 | 3 | 4 | 5 | 2 |
| succinate dehydrogenase activity | 2 | 3 | 3 | 3 | 4 |
| hydrolase activity, acting on carbon-nitrogen (but not peptide) bonds, in cyclic amides | 2 | 2 | 4 | 3 | 4 |
| DNA photolyase activity | 2 | 2 | 2 | 3 | 2 |
| Ras GTPase activator activity | 2 | 3 | 3 | 1 | 2 |
| transcription corepressor activity | 2 | 2 | 2 | 2 | 2 |
| acid-ammonia (or amide) ligase activity | 2 | 2 | 2 | 2 | 2 |
| hedgehog receptor activity | 2 | 2 | 2 | 2 | 2 |
| thiamine-phosphate diphosphorylase activity | 2 | 2 | 2 | 2 | 2 |
| sedoheptulose-7-phosphate:D-glyceraldehyde-3-phosphate glyceronetransferase activity | 2 | 2 | 2 | 2 | 2 |
| holocytochrome-c synthase activity | 2 | 2 | 2 | 2 | 2 |
| phosphatidic acid binding | 2 | 2 | 2 | 2 | 2 |
| oxidoreductase activity, acting on a sulfur group of donors, disulfide as acceptor | 2 | 2 | 2 | 1 | 2 |
| argininosuccinate synthase activity | 2 | 1 | 2 | 2 | 2 |
| hydrolase activity, hydrolyzing N-glycosyl compounds | 2 | 1 | 2 | 2 | 2 |
| DNA topoisomerase type I activity | 2 | 2 | 2 | 2 | 1 |
| riboflavin synthase activity | 2 | 2 | 2 | 1 | 2 |
| DNA topoisomerase type II (ATP-hydrolyzing) activity | 2 | 2 | 2 | 2 | 1 |
| protein kinase activator activity | 2 | 2 | 2 | 1 | 2 |
| ER retention sequence binding | 2 | 2 | 2 | 2 | 1 |
| oxidoreductase activity, acting on paired donors, with incorporation or reduction of molecular oxygen, reduced flavin or flavoprotein as one donor, and incorporation of one atom of oxygen | 2 | 0 | 2 | 2 | 2 |
| oligopeptide transporter activity | 2 | 2 | 1 | 1 | 2 |
| cysteine synthase activity | 2 | 2 | 2 | 2 | 0 |
| fatty-acyl-CoA binding | 2 | 2 | 2 | 0 | 1 |
| double-stranded RNA binding | 1 | 3 | 2 | 1 | 3 |
| oxidoreductase activity, acting on NAD(P)H, heme protein as acceptor | 1 | 2 | 2 | 2 | 2 |
| histone acetyl-lysine binding | 1 | 0 | 5 | 1 | 2 |
| oxidoreductase activity, acting on the CH-NH2 group of donors, oxygen as acceptor | 1 | 0 | 4 | 0 | 4 |
| cyanate hydratase activity | 1 | 1 | 4 | 1 | 1 |
| racemase and epimerase activity, acting on amino acids and derivatives | 1 | 0 | 3 | 3 | 1 |
| DNA ligase activity | 1 | 1 | 1 | 2 | 2 |
| phosphotransferase activity, carboxyl group as acceptor | 1 | 3 | 1 | 1 | 1 |
| lactoylglutathione lyase activity | 1 | 1 | 1 | 1 | 3 |
| transferase activity, transferring acyl groups, acyl groups converted into alkyl on transfer | 1 | 1 | 3 | 1 | 1 |
| DNA clamp loader activity | 1 | 1 | 1 | 2 | 1 |
| oxidoreductase activity, acting on paired donors, with incorporation or reduction of molecular oxygen, NAD(P)H as one donor, and incorporation of one atom of oxygen | 1 | 1 | 1 | 1 | 2 |
| hydroxymethylbilane synthase activity | 1 | 1 | 1 | 1 | 2 |
| 3-phosphoshikimate 1-carboxyvinyltransferase activity | 1 | 1 | 1 | 1 | 2 |
| oxidoreductase activity, acting on the aldehyde or oxo group of donors, disulfide as acceptor | 1 | 1 | 1 | 1 | 2 |
| CTP synthase activity | 1 | 1 | 1 | 1 | 2 |
| NADPH dehydrogenase activity | 1 | 0 | 1 | 1 | 3 |
| pyruvate carboxylase activity | 1 | 1 | 1 | 2 | 1 |
| N-acetylglucosamine-6-phosphate deacetylase activity | 1 | 1 | 1 | 1 | 2 |
| carbon-oxygen lyase activity, acting on polysaccharides | 1 | 1 | 1 | 1 | 2 |
| all-trans retinal binding | 1 | 1 | 2 | 1 | 1 |
| phospholipid-hydroperoxide glutathione peroxidase activity | 1 | 1 | 1 | 1 | 1 |
| methionine adenosyltransferase activity | 1 | 1 | 1 | 1 | 1 |
| S-(hydroxymethyl)glutathione synthase activity | 1 | 1 | 1 | 1 | 1 |
| dihydropteroate synthase activity | 1 | 1 | 1 | 1 | 1 |
| RNA cap binding | 1 | 1 | 1 | 1 | 1 |
| transferase activity, transferring pentosyl groups | 1 | 1 | 1 | 1 | 1 |
| oxidoreductase activity, acting on a sulfur group of donors, oxygen as acceptor | 1 | 1 | 1 | 1 | 1 |
| G-protein coupled receptor binding | 1 | 1 | 1 | 1 | 1 |
| molybdopterin cofactor binding | 1 | 1 | 1 | 1 | 1 |
| 3,4-dihydroxy-2-butanone-4-phosphate synthase activity | 1 | 1 | 1 | 1 | 1 |
| protein phosphatase inhibitor activity | 1 | 1 | 1 | 1 | 1 |
| deoxyhypusine synthase activity | 1 | 1 | 1 | 1 | 1 |
| pyruvate dehydrogenase activity | 1 | 1 | 1 | 1 | 1 |
| cystathionine gamma-lyase activity | 1 | 1 | 1 | 1 | 1 |
| cystathionine beta-lyase activity | 1 | 1 | 1 | 1 | 1 |
| DNA-(apurinic or apyrimidinic site) lyase activity | 1 | 1 | 1 | 1 | 1 |
| carboxyl- or carbamoyltransferase activity | 1 | 1 | 1 | 1 | 1 |
| acetyl-CoA transporter activity | 1 | 1 | 1 | 1 | 1 |
| sterol transporter activity | 1 | 1 | 1 | 1 | 1 |
| AU-rich element binding | 1 | 1 | 1 | 1 | 1 |
| formate-tetrahydrofolate ligase activity | 1 | 1 | 1 | 1 | 1 |
| formaldehyde transketolase activity | 1 | 1 | 1 | 1 | 1 |
| trans-hexaprenyltranstransferase activity | 1 | 1 | 1 | 1 | 1 |
| spermidine synthase activity | 1 | 1 | 1 | 1 | 1 |
| squalene monooxygenase activity | 1 | 1 | 1 | 1 | 1 |
| G-protein beta/gamma-subunit complex binding | 1 | 1 | 1 | 1 | 1 |
| glycolipid transporter activity | 1 | 1 | 1 | 1 | 1 |
| channel activity | 1 | 1 | 1 | 1 | 1 |
| inositol-3-phosphate synthase activity | 1 | 1 | 1 | 1 | 1 |
| phytoene dehydrogenase activity | 1 | 1 | 1 | 1 | 1 |
| G-protein coupled photoreceptor activity | 1 | 1 | 1 | 1 | 1 |
| peptidase activity, acting on L-amino acid peptides | 1 | 1 | 2 | 0 | 1 |
| cAMP-dependent protein kinase regulator activity | 1 | 1 | 1 | 1 | 0 |
| protein kinase inhibitor activity | 1 | 0 | 1 | 1 | 1 |
| UDP-galactopyranose mutase activity | 1 | 0 | 1 | 1 | 1 |
| sulfotransferase activity | 1 | 1 | 1 | 1 | 0 |
| endoplasmic reticulum signal peptide binding | 1 | 0 | 1 | 1 | 1 |
| cAMP binding | 1 | 1 | 1 | 1 | 0 |
| GTPase binding | 1 | 0 | 1 | 1 | 1 |
| 5-(carboxyamino)imidazole ribonucleotide mutase activity | 1 | 0 | 1 | 1 | 1 |
| inositol oxygenase activity | 1 | 1 | 1 | 1 | 0 |
| heme oxygenase (decyclizing) activity | 1 | 0 | 1 | 1 | 1 |
| DNA binding, bending | 1 | 1 | 1 | 0 | 1 |
| cyclin-dependent protein serine/threonine kinase regulator activity | 1 | 1 | 1 | 0 | 1 |
| cytoskeletal adaptor activity | 1 | 1 | 1 | 0 | 1 |
| SH3 domain binding | 1 | 1 | 1 | 0 | 1 |
| thiolester hydrolase activity | 1 | 1 | 0 | 1 | 0 |
| cellobiose dehydrogenase (acceptor) activity | 1 | 0 | 1 | 1 | 0 |
| intramolecular transferase activity, phosphotransferases | 1 | 1 | 0 | 1 | 0 |
| chorismate mutase activity | 1 | 1 | 0 | 0 | 1 |
| sterol binding | 1 | 1 | 1 | 0 | 0 |
| pre-mRNA binding | 1 | 0 | 0 | 1 | 0 |
| FK506 binding | 1 | 1 | 0 | 0 | 0 |
| deoxyhypusine monooxygenase activity | 0 | 1 | 1 | 1 | 1 |
| 3-carboxy-cis,cis-muconate cycloisomerase activity | 0 | 0 | 2 | 1 | 1 |
| hydro-lyase activity | 0 | 0 | 0 | 1 | 1 |
| protease binding | 0 | 1 | 0 | 0 | 1 |
| RNA polymerase II distal enhancer sequence-specific DNA binding transcription factor activity | 0 | 1 | 1 | 0 | 0 |
| nitrate reductase activity | 0 | 0 | 0 | 1 | 0 |
| chalcone isomerase activity | 0 | 1 | 0 | 0 | 0 |
| Hsp90 protein binding | 0 | 1 | 0 | 0 | 0 |
| urea carboxylase activity | 0 | 1 | 0 | 0 | 0 |
| phosphotransferase activity, phosphate group as acceptor | 0 | 0 | 1 | 0 | 0 |
| ammonia-lyase activity | 0 | 0 | 1 | 0 | 0 |
| ether hydrolase activity | 0 | 0 | 0 | 0 | 1 |
| guanyl nucleotide binding | 0 | 0 | 0 | 0 | 1 |
| hydrolase activity, acting on carbon-nitrogen (but not peptide) bonds, in cyclic amidines | 0 | 0 | 0 | 0 | 1 |
| phosphonopyruvate hydrolase activity | 0 | 0 | 0 | 0 | 1 |
| O-acetylhomoserine aminocarboxypropyltransferase activity | 0 | 0 | 0 | 0 | 1 |

| ***Table S5: Functional analysis, GO category Cellular Components*** | | | | | |
| --- | --- | --- | --- | --- | --- |
| GO category | FA | FG | FO | FV | FS |
| cytoplasm | 303 | 292 | 361 | 295 | 310 |
| endoplasmic reticulum membrane | 34 | 38 | 39 | 35 | 42 |
| ribonucleoprotein complex | 29 | 28 | 28 | 29 | 23 |
| proteasome complex | 20 | 23 | 22 | 21 | 16 |
| intrinsic to endoplasmic reticulum membrane | 19 | 17 | 17 | 18 | 21 |
| large ribosomal subunit | 11 | 10 | 14 | 14 | 8 |
| soluble fraction | 12 | 12 | 11 | 10 | 11 |
| cell wall | 8 | 9 | 11 | 9 | 16 |
| small ribosomal subunit | 10 | 10 | 11 | 11 | 9 |
| membrane coat | 8 | 8 | 9 | 10 | 6 |
| mating projection tip | 9 | 7 | 8 | 8 | 8 |
| proteasome core complex | 8 | 7 | 8 | 6 | 8 |
| cell outer membrane | 7 | 8 | 8 | 5 | 7 |
| proteasome core complex, alpha-subunit complex | 7 | 7 | 7 | 7 | 7 |
| protein kinase CK2 complex | 7 | 5 | 9 | 5 | 5 |
| respiratory chain | 6 | 7 | 5 | 5 | 6 |
| cytoplasmic part | 8 | 6 | 3 | 5 | 5 |
| prospore membrane | 6 | 5 | 5 | 5 | 6 |
| ubiquitin ligase complex | 5 | 5 | 5 | 5 | 3 |
| mitochondrial intermembrane space protein transporter complex | 5 | 4 | 5 | 4 | 5 |
| mating projection base | 5 | 4 | 4 | 4 | 4 |
| transcription factor TFIID complex | 4 | 3 | 4 | 4 | 3 |
| nuclear envelope | 3 | 3 | 3 | 3 | 5 |
| clathrin coat of coated pit | 3 | 3 | 4 | 3 | 3 |
| Elongator holoenzyme complex | 3 | 3 | 3 | 3 | 3 |
| signal peptidase complex | 3 | 3 | 3 | 3 | 3 |
| extrinsic to membrane | 3 | 3 | 3 | 2 | 3 |
| protein phosphatase type 2A complex | 3 | 3 | 3 | 3 | 2 |
| cell cortex | 3 | 1 | 4 | 3 | 3 |
| intracellular organelle | 3 | 2 | 0 | 2 | 4 |
| transcription factor TFIIA complex | 2 | 2 | 3 | 2 | 2 |
| incipient cellular bud site | 2 | 2 | 2 | 2 | 2 |
| oxoglutarate dehydrogenase complex | 3 | 2 | 2 | 2 | 1 |
| calcineurin complex | 1 | 2 | 2 | 2 | 2 |
| mating projection | 2 | 1 | 2 | 2 | 2 |
| outer membrane | 2 | 3 | 1 | 2 | 1 |
| intrinsic to vacuolar membrane | 2 | 2 | 2 | 1 | 2 |
| Vps55/Vps68 complex | 2 | 2 | 2 | 1 | 2 |
| holo TFIIH complex | 2 | 2 | 2 | 1 | 2 |
| proton-transporting ATP synthase complex, coupling factor F(o) | 1 | 2 | 2 | 2 | 1 |
| SNARE complex | 2 | 1 | 1 | 1 | 2 |
| proteasome regulatory particle | 1 | 1 | 1 | 1 | 1 |
| sulfite reductase complex (NADPH) | 1 | 1 | 1 | 1 | 1 |
| transcription factor TFIIE complex | 1 | 1 | 1 | 1 | 1 |
| ubiquitin conjugating enzyme complex | 1 | 1 | 1 | 1 | 1 |
| cellular bud neck contractile ring | 1 | 0 | 2 | 1 | 1 |
| TORC1 complex | 1 | 1 | 1 | 1 | 1 |
| transcription factor TFIIF complex | 1 | 1 | 1 | 1 | 1 |
| RNA polymerase complex | 1 | 1 | 1 | 1 | 1 |
| EKC/KEOPS complex | 1 | 1 | 1 | 1 | 1 |
| retromer complex, outer shell | 1 | 1 | 1 | 1 | 1 |
| PCNA complex | 1 | 1 | 1 | 1 | 1 |
| TORC2 complex | 1 | 1 | 1 | 1 | 1 |
| UDP-N-acetylglucosamine transferase complex | 1 | 1 | 1 | 1 | 1 |
| Cdc48p-Npl4p-Ufd1p AAA ATPase complex | 1 | 1 | 1 | 1 | 1 |
| ESCRT III complex | 1 | 1 | 1 | 1 | 1 |
| cell wall-bounded periplasmic space | 1 | 1 | 1 | 1 | 1 |
| nuclear cap binding complex | 1 | 1 | 1 | 1 | 1 |
| filamentous actin | 1 | 1 | 1 | 1 | 1 |
| succinate dehydrogenase complex | 0 | 1 | 1 | 1 | 1 |
| core TFIIH complex | 1 | 1 | 1 | 1 | 0 |
| tight junction | 0 | 1 | 1 | 1 | 1 |
| oligosaccharyltransferase complex | 1 | 1 | 1 | 1 | 0 |
| cAMP-dependent protein kinase complex | 1 | 1 | 1 | 1 | 0 |
| cyclin-dependent protein kinase holoenzyme complex | 1 | 1 | 1 | 0 | 1 |
| proton-transporting ATP synthase complex, catalytic core F(1) | 0 | 1 | 1 | 1 | 0 |
| plasma membrane part | 1 | 2 | 0 | 0 | 0 |
| mismatch repair complex | 0 | 0 | 1 | 1 | 0 |
| proton-transporting two-sector ATPase complex | 0 | 0 | 1 | 0 | 0 |
| intrinsic to membrane | 1 | 0 | 0 | 0 | 0 |
| outer membrane-bounded periplasmic space | 1 | 0 | 0 | 0 | 0 |
| proton-transporting two-sector ATPase complex, catalytic domain | 0 | 0 | 0 | 0 | 1 |

| ***Table S6. Secretome of Fa05001, fisher’s exact test (FDR < 0.05)*** | | | | |
| --- | --- | --- | --- | --- |
| GO-ID | Term | Category | FDR | P-Value |
| GO:0005576 | extracellular region | C | 9.3E-46 | 1.02E-48 |
| GO:0055114 | oxidation-reduction process | P | 3.4E-22 | 6.8E-25 |
| GO:0030248 | cellulose binding | F | 3.9E-14 | 1.5E-16 |
| GO:0045493 | xylan catabolic process | P | 4.3E-13 | 2.3E-15 |
| GO:0050660 | flavin adenine dinucleotide binding | F | 5.4E-13 | 3.0E-15 |
| GO:0006508 | proteolysis | P | 1.1E-11 | 6.9E-14 |
| GO:0008812 | choline dehydrogenase activity | F | 8.9E-8 | 6.8E-10 |
| GO:0030570 | pectate lyase activity | F | 1.5E-6 | 1.3E-8 |
| GO:0004252 | serine-type endopeptidase activity | F | 4.7E-6 | 4.1E-8 |
| GO:0005618 | cell wall | C | 6.5E-6 | 5.9E-8 |
| GO:0008762 | UDP-N-acetylmuramate dehydrogenase activity | F | 1.7E-5 | 1.6E-7 |
| GO:0052051 | interaction with host via protein secreted by type II secretion system | P | 1.8E-5 | 2.1E-7 |
| GO:0045490 | pectin catabolic process | P | 1.8E-5 | 2.1E-7 |
| GO:0004568 | chitinase activity | F | 2.0E-5 | 2.5E-7 |
| GO:0006032 | chitin catabolic process | P | 2.0E-5 | 2.5E-7 |
| GO:0004185 | serine-type carboxypeptidase activity | F | 1.3E-4 | 1.7E-6 |
| GO:0004190 | aspartic-type endopeptidase activity | F | 1.3E-4 | 1.8E-6 |
| GO:0050661 | NADP binding | F | 2.6E-4 | 3.8E-6 |
| GO:0016998 | cell wall macromolecule catabolic process | P | 3.1E-4 | 4.5E-6 |
| GO:0004181 | metallocarboxypeptidase activity | F | 4.1E-4 | 6.3E-6 |
| GO:0008810 | cellulase activity | F | 4.1E-4 | 6.3E-6 |
| GO:0004650 | polygalacturonase activity | F | 4.1E-4 | 6.4E-6 |
| GO:0030245 | cellulose catabolic process | P | 5.3E-4 | 8.8E-6 |
| GO:0046556 | alpha-N-arabinofuranosidase activity | F | 5.3E-4 | 8.8E-6 |
| GO:0050525 | cutinase activity | F | 0.002 | 3.5E-5 |
| GO:0031176 | endo-1,4-beta-xylanase activity | F | 0.002 | 3.5E-5 |
| GO:0008061 | chitin binding | F | 0.002 | 4.6E-5 |
| GO:0051707 | response to other organism | P | 0.009 | 1.9E-4 |
| GO:0047490 | pectin lyase activity | F | 0.009 | 1.9E-4 |
| GO:0004499 | N,N-dimethylaniline monooxygenase activity | F | 0.011 | 2.2E-4 |
| GO:0016209 | antioxidant activity | F | 0.027 | 5.9E-4 |
| GO:0007155 | cell adhesion | P | 0.032 | 7.1E-4 |
| GO:0045735 | nutrient reservoir activity | F | 0.047 | 0.001 |
| GO:0004806 | triglyceride lipase activity | F | 0.047 | 0.001 |
| GO:2001070 | starch binding | F | 0.047 | 0.001 |
| GO:0006952 | defense response | P | 0.047 | 0.001 |
| GO:0005199 | structural constituent of cell wall | F | 0.047 | 0.001 |

| ***Table S7: Putative small cysteine rich proteins in Fa05001*** | | | |
| --- | --- | --- | --- |
| **Scaffold** | **Gene name** | **Sequence (over four cysteines), significant hits to the HMM model DB** | ***Fusarium* orthologs**  **Bit-score>50** |
| Scaffold 1 | CRP0850 | MFTPNLLMRGPFFCQCIYTHPHKLSYRCPASAYC |  |
| Scaffold 2 | CRP2980 | MYCWDEGTRTEAPACMISCSCVT |  |
| Scaffold 5 | CRP0720 | MWLGKLMGSKLLLPFGLCRDLYCCCFYGRLLG |  |
| Scaffold 5 | CRP5760 | MTKDVASQDGTCGDKVNLTCAGGPFNGQCCSAAGFCGTSTHCGSGW | FG, FO |
| Scaffold 6 | CRP4920 | MGSSRSFGREPCCSGLSSSLLSSSTPSSAQCCQNLRD | FG, FO, FV, FS |
| Scaffold 6 | CRP4630 | MSQCECGCSLFHGGQINSLGLSIDQYQPMEEPVRNKSSWLLEQIPGTAVC |  |
| Scaffold 9 | CRP1140 | MRKQSMSTSNRLDFECSQDADCPRQCFQISTRCMQLSRK |  |
| Scaffold 10 | CRP1006 | MGGGKIMKQNTTITCSRRCKQRRYTYMGRCICK |  |
| Scaffold 14 | CRP1910 | MQIYALPIRARILPEPRLPRSGRSKYFHQCIHCVPCVVIMNDGWCCFRH |  |
| Scaffold 15 | CRP0560 | MLVVGKGGDDECSSACCRKRLWGKGICKQSVHIQPSPST |  |
| Scaffold 21 | CRP0630 | MVLPIPISRSSGPCEEATAVWCNPGPAILIYRDVVSDCPPDSCNFEKV |  |
| Scaffold 21 | CRP1190 | MFQLQPRHCRHSRCVYQSFYPNCLLAADDC | FG, FO, FV, FS |
| Scaffold 22 | CRP1019 | MPIVLCPWCKSKFRVPCRGVVRKRPSLRADCILVTSSMLGI |  |
| Scaffold 26 | CRP0340 | MAASSFLTCMCICIGWDGVYNHGRCAKDRPCVYYMG |  |
| Scaffold 29 | CRP2610 | MRIVRQPSATDKCLQDCCPGSCWYSRHKVQYAQLA | FO |
| Scaffold 33 | CRP4260 | MLGCHFPRPEHTCCQGSRGSSSYRCVQ |  |
| Scaffold 36 | CRP5810 | MCGEYSEDGDMPCGMNLCCSASGWCGVSNFILASFFPHFSNTYVPYYLLF | FG, FO |
| Scaffold 44 | CRP2880 | MKPHLCRPEPLLEYHLGPLMTLSAGNQCAGSCTKGCGIQARG |  |
| Scaffold 44 | CRP0860 | MVSTPRCDCVPILVSSSRHQCYC |  |
|  |  |  |  |
| **Scaffold** | **Gene name** | **Sequence (under four cysteines), significant hits to the HMM model DB** |  |
| Scaffold 1 | CRP3150 | MGPNCHKRDGEEDCHSLRKYCIV |  |
| Scaffold 1 | CRP5545 | MENRNRSRVWSKNTNHIIKASYMLCPSSRSQI |  |
| Scaffold 1 | CRP1020 | MLRGSLFGVNCPWCKKGRSLDAVSRIHIY |  |
| Scaffold 2 | CRP5102 | MKRYRLAQYPDQYRCQ |  |
| Scaffold 2 | CRP2230 | MRCRNSVCDSMTDLVESRNSLSRDVA | FG, FO, FV |
| Scaffold 2 | CRP4060 | MATQSDVFNSLKPRVSPMASGQGQPAQAVFLNA |  |
| Scaffold 2 | CRP4440 | MPPTALPWRPVEEARRPALLPSCAAAAPWEYIISHLYCSSA | FG, FO, FV, FS |
| Scaffold 2 | CRP2360 | MYAAFCMWFCLSRSLM |  |
| Scaffold 3 | CRP3860 | MVSPKSIDVAVASSLPSKCSSI |  |
| Scaffold 3 | CRP2030 | MSSLRTPSAATCGGSCPISSLPYTIRAHLSLEPRASCS | FG, FO, FV, FS |
| Scaffold 4 | CRP4540 | MRIQWLGLSSVCGVQTPMVS |  |
| Scaffold 4 | CRP5220 | MKSVHEQTQCSCVKLPPVLSRCWQGHVEVIVVEEYERGAVY | FG, FO |
| Scaffold 5 | CRP0290 | MESEGFMDGPWCDHPR | FS |
| Scaffold 5 | CRP5300 | MLHDTCGSVSQGCEYSHGPWRTQSRQCDTAHTSHNGNEVLHQSEKDLESH | FG, FO, FV, FS |
| Scaffold 5 | CRP2220 | MTRNTVFESGSSSLDYVQLGCQSSVCYSRAVETKISVLLSR |  |
| Scaffold 5 | CRP4220 | MRRCLCSHKGSVWLRSSVERSTENGTP | FG, FO, FV, FS |
| Scaffold 5 | CRP5077 | MPQTFPGCQTQSTNQGSSRIRQICSNYVSEGQTNTTHR |  |
| Scaffold 5 | CRP5495 | MNLFLHGKCNFRKTLECYAVLDVILETCK |  |
| Scaffold 5 | CRP4625 | MRRPHFVFLTFLSSHSSDPDSACCSRYDQPISFEDHP | FO, FV, FS |
| Scaffold 7 | CRP2855 | MRRLTTRSDKLLQCPSHCVIPFH | FG |
| Scaffold 7 | CRP4310 | MDDALNFSSHILNVPAICAYLGSP | FG, FO, FV, FS |
| Scaffold 8 | CRP3620 | MNLWYMNLIVLSSFFSCTRPNRHPWTAPCPA |  |
| Scaffold 8 | CRP1040 | MLELQEIMIRYRENQNCRCAHGE |  |
| Scaffold 9 | CRP1270 | MKFCCPKGLCEHTRHVYVMFAEQG |  |
| Scaffold 10 | CRP0730 | MQRPPTRVGWAMKTCLSNEACERSCEVSHINRNHKSNV | FG, FO, FV |
| Scaffold 11 | CRP5510 | MRIFYCFVYILPDRYCWKTIEEV |  |
| Scaffold 11 | CRP3480 | MGGFYMQYLESKSITTYIFHYCQVIVSATPAIA | FV |
| Scaffold 11 | CRP5480 | MMLTLTKHEATCICCSV |  |
| Scaffold 11 | CRP4630 | MWVVKKKPPCLCSLCVVGGGNFG | FV |
| Scaffold 11 | CRP0731 | MHGHVQSYVSKNSSASCATGPKSSHRRVNA |  |
| Scaffold 11 | CRP0600 | MCYIAYAAVGRESACAGAASKCRTWDER |  |
| Scaffold 13 | CRP3865 | MLEAFQLIDIHNHILKKCEVSCRYKTSPSLSCLILLRQRVIRRLL |  |
| Scaffold 16 | CRP4948 | MFVLEVSRLKDWCCHQLHLSGSPECRVLPPGKQVREGRDPV |  |
| Scaffold 17 | CRP5545 | MQYVRFICMSSIAYGYCPESYTETIMSWW |  |
| Scaffold 17 | CRP0700 | MTVEVTAGVAREVEMAVDKCKDFVVGRSYCNNAAQPTAAVAWCQTDKGSL |  |
| Scaffold 17 | CRP0550 | MAFQELYPDPICMDVNCRWMCMIGWVPGS |  |
| Scaffold 18 | CRP4948 | MYRYVGFMLSTCHFYVAHRGCKACSTMKPTRYIRMIANLS |  |
| Scaffold 18 | CRP4921 | MFMSIRYISSSQPSTTCCSRLIIHSTLTCLRARPVGMRGV |  |
| Scaffold 18 | CRP1018 | MTTLFVMINEEQTLCPWCKTDIHETKGRAKKPWTFSVHLLYKGFDTTP | FG, FO, FV, FS |
| Scaffold 18 | CRP5050 | MSRRKTTLPFPKSWTKRSESEVKTVPPDSDTCCAQYASASPGIRCTSFA | FG, FO, FV, FS |
| Scaffold 19 | CRP1320 | MCNGDYHDTCIRRLYLCPIGGRADE | FG |
| Scaffold 19 | CRP1008 | MQKVSPCFQCRTASPTFVKPEPSTT | FG, FS |
| Scaffold 21 | CRP6155 | MGKRIVQRQKPNAMQSPPNKMQLGKRTYSFARSLEMPVPLVCLGLPLRRF | FG, FO, FV |
| Scaffold 21 | CRP4730 | MLELALKPFCLCAVMMQGIGKYVTKGAF |  |
| Scaffold 22 | CRP4520 | MSRSKLPQLLEPLRLLHDPSQCPLHLPPNVLLLAASLPSSSSVTLV | FO |
| Scaffold 22 | CRP1150 | MFKCFTSSGCPSLPCIEILK |  |
| Scaffold 24 | CRP1790 | MSIAWSIRRWSRNCRTTVGQCAKVPSQTTESYFL |  |
| Scaffold 25 | CRP4690 | MGACCNSLATLTSNGTRCPLHSQHQAFMYNSHASLIFVPYRHHGVTI |  |
| Scaffold 26 | CRP3950 | MSGQYAVDKGHLQPCIAYKAGWCPRQASGCGDGLRSRRRGPGWLI | FG, FO, FV, FS |
| Scaffold 27 | CRP1650 | MQRNISDLQSEKKALNVIAVGCICIPIVTSPGGECIHSSVDSHQK |  |
| Scaffold 27 | CRP0040 | MCIIHGTCSNLRLLNCVS |  |
| Scaffold 28 | CRP4040 | MRPTLALRRSRLDPILPQCRCNGLLVAWKVPVGNSHRIAEPCKPL | FG, FO, FV, FS |
| Scaffold 29 | CRP0700 | MAESGNCTWQNPLWECLSDFCSFGQ |  |
| Scaffold 29 | CRP2345 | MHSASRWSRVAVRYCKVCGCSLQQLLKAMLW |  |
| Scaffold 31 | CRP4380 | MTVLTPGNDCLCNAMRAMRSQL |  |

| ***Table S8: The apicidin-like gene cluster^1^ in the three F. avenaceum strains Fa05001, FaLH03 and FaLH27*** | | | | | | | | | | | | |
| --- | --- | --- | --- | --- | --- | --- | --- | --- | --- | --- | --- | --- |
| **Apicidin/HC-toxin**  **cluster names** | **Predicted function (CDD + blastP)** | **Verified**  **Function^2^** | **Gene in *F. avenaceum*** | | | **aa I% Fi vs**  **Fa05001** | **Found in cluster** | | | | | |
|  |  |  | **Fa05001** | **FaLH03** | **FaLH27** |  | **Fi** | **Ff** | **Fa** | **Cc** | **Ptr** | **St** |
| *Aps1 / HTS1* | Non-ribosomal peptide synthetase | Yes | *FAVG1_08578* | *FAVG2_02884* | *FAVG3_02923* | 45 | + | + | + | + | + | + |
| *Aps2 / ToxE* | Transcription factor (bzip) | Yes | *FAVG1_08585* | *FAVG2_02891* | *FAVG3_02930* | 46 | + | + | + | + | + | + |
| *Aps3* | Pyrroline reductase | Yes | *FAVG1_09639* | *FAVG2_01813* | *FAVG3_01848* | 44 | + | + | N | N | N | N |
| *Aps4 / ToxF* | Aminotransferase (PLPDE_IV) | No | *FAVG1_08580* | *FAVG2_02886* | *FAVG3_02925* | 55 | + | + | + | + | + | + |
| *Aps5 / FAS a* | Fatty acid synthase a subunit | Yes | *FAVG1_08584* | *FAVG2_02890* | *FAVG3_02929* | 55 | + | + | + | N | + | + |
| *Aps6* | O-methyltransferase | Yes | *FAVG1_12700* | *FAVG2_02002* | *FAVG3_02039* | 31 | + | + | N | N | N | N |
| *Aps7* | Cytochrome P450 monooxygenase | Yes | *FAVG1_08574* | *FAVG2_02880* | *FAVG3_02919* | 73 | + | + | + | N | + | + |
| *Aps8* | Cytochrome P450 monooxygenase | Yes | *FAVG1_08576* | *FAVG2_02882* | *FAVG3_02921* | 65 | + | + | + | N | + | + |
| *Aps9* | Oxidase | Yes | *FAVG1_08582* | *FAVG2_02888* | *FAVG3_02927* | 54 | + | + | + | N | + | + |
| *Aps10* | Reductase (norsolorinic acid reductase like) | No | *FAVG1_08575* | *FAVG2_02881* | *FAVG3_02920* | 73 | + | N | + | N | + | + |
| *Aps11 / ToxA* | Major facilitator Superfamily (efflux pump) | Unknown | *FAVG1_08577* | *FAVG2_02883* | *FAVG3_02922* | 50 | + | + | + | + | + | + |
| *Aps12* | Cytochrome b5-like Heme/Steroid binding domain + Ferredoxin reductase domain | Unknown | *FAVG1_02139* | *FAVG2_10285* | *FAVG3_10367* | 29 | + | + | N | N | N | N |
| *Aps13* | Protein unknown func. (DUF829) | Unknown | *FAVG1_08579* | *FAVG2_02885* | *FAVG3_02924* | Na | N | N | + | N | N | N |
| *Aps14* | Fatty acid synthase b subunit | Unknown | *FAVG1_08581* | *FAVG2_02887* | *FAVG3_02926* | Na | N | N | + | N | N | N |
| *Aps15* | Cysteine synthase like | Unknown | *FAVG1_08583* | *FAVG2_02889* | *FAVG3_02928* | Na | N | N | + | N | N | N |

^1^The apicidin-like gene cluster in the three *F. avenaceum strains* compared to the characterized apicidin gene cluster from *F. incamatum* (Fi) and the HC-toxin gene

clusters from *Cochliobolus carbonum* (Cc), *Pyrenophora tritici-repentis* (Ptr) and *Setosphaeria turcica* (St). ^2^Based on the result from Jin *et al.* [1].

| **Table S9:** Protein similarity between gibberellic acid biosynthetic enzymes from *F. avenaceum* 05001, FaLH03 and FaLH27, *F. fujikuroi* MP-A, *F. proliferatum* KGL0401 and *S. manihoticola* | | | | | | | | | |
| --- | --- | --- | --- | --- | --- | --- | --- | --- | --- |
|  | ***F. avenaceum*** | ***F. avenaceum*** | ***F. avenaceum*** | ***F. fujikuroi*** |  | ***F. proliferatum*** | | ***S. manihoticola*** | |
| **Function (name)** | **Fa05001** | **FaLH03** | **FaLH27** | **Gene no.** | **Identity** | **Gene no.** | **Identity** | **Gene no.** | **Identity** |
| *ent-*kaurene (*P450-4*) | *FAVG1_10697* | *FAVG2_04186* | *FAVG3_04219* | *FFUJ_14332* | 72.2% | *EF119832* | 73.1% | *AM886289* | 46.5% |
| GA_14_-synthase (*P450-1*) | *FAVG1_10698* | *FAVG2_04187* | *FAVG3_04220* | *FFUJ_14333* | 68.3% | *DQ313173* | 74.0% | *AM886288* | 54.3% |
| C20-oxidase (*P450-2*) | *FAVG1_10699* | *FAVG2_04188* | *FAVG3_04221* | *FFUJ_14334* | 69.9% | *DQ313174* | 77.5% | *AM886290* | 51.1% |
| GGDP-synthase (*gss2*) | *FAVG1_10700* | *FAVG2_04189* | *FAVG3_04222* | *FFUJ_14335* | 54.0% | *DQ313175* | 53.4% | *AM886291* | 29.6% |
| CPS/ent-kaurene synthase (*cps/ks*) | *FAVG1_10701* | *FAVG2_04190* | *FAVG3_04223* | *FFUJ_14336* | 65.0% | *DQ313176* | 64.2% | *AM886292* | 45.0% |
| GA_4_ desaturase (*des*) | *FAVG1_10702* | *FAVG2_04191* | *FAVG3_04224* | *FFUJ_14331* | 51.2% | *EF119831* | 51.2% | nd | nd |
| C13-oxidase (*P450-3*) | nd | nd | nd | *FFUJ_14337* | nd | *DQ313177* | nd | nd | nd |
| **Main end-product** |  | Unknown |  | GA 1 and GA3 | | GA3 |  | GA4 |  |

| ***Table S10****. Expression of Fa05001 genes in barley 14 dpi, FDR < 0.05.* | | | | | |
| --- | --- | --- | --- | --- | --- |
| Gene | P value | FDR P-value correction | Annotation | Mean RPKM | Putative function^1^ |
| *FAVG1_00290* | 2.40E-10 | 3.20E-06 | GATase1_Hsp31_like | 571 | Oxidative stress |
| *FAVG1_11042* | 5.80E-10 | 3.90E-06 | Myosin-5 | 27 | Signal Transduction |
| *FAVG1_00614* | 5.90E-09 | 2.00E-05 | NADH:ubiquinone oxidoreductase subunit | 632 | Oxidative stress / Signal transduction |
| *FAVG1_01762* | 6.10E-09 | 2.00E-05 | ATP synthase subunit 5 | 1040 | Energy |
| *FAVG1_03988* | 2.10E-08 | 5.60E-05 | Heat shock 70 kda mitochondrial precursor | 356 | Response to stress |
| *FAVG1_03017* | 3.60E-08 | 7.90E-05 | Aconitate hydratase | 420 | Oxidative stress |
| *FAVG1_00271* | 1.50E-07 | 2.40E-04 | Hypothetical protein | 124 | Unknown |
| *FAVG1_12917* | 1.50E-07 | 2.40E-04 | Mitochondrial carrier protein YHM1 | 70 | Iron distribution |
| *FAVG1_03007* | 1.70E-07 | 2.40E-04 | Cell wall glucanase | 47 | Hydrolytic enzyme |
| *FAVG1_09390* | 2.60E-07 | 3.40E-04 | Glycine-rich rna binding protein | 302 | Response to stress |
| *FAVG1_11696* | 2.90E-07 | 3.50E-04 | Glutathione transferase | 66 | Oxidative stress |
| *FAVG1_02309* | 1.00E-06 | 1.20E-03 | Aldehyde reductase | 125 | Response to stress |
| *FAVG1_12609* | 1.10E-06 | 1.20E-03 | Galactose oxidase | 60 | Oxidoreductase |
| *FAVG1_10070* | 1.40E-06 | 1.20E-03 | Glutathione-independent formaldehyde dehydrogenase | 135 | Response to stress |
| *FAVG1_05522* | 1.40E-06 | 1.20E-03 | Glucan synthesis regulatory protein | 200 | Response to stress |
| *FAVG1_08017* | 1.50E-06 | 1.20E-03 | Coiled-coil domain-containing | 450 | Oxidative stress |
| *FAVG1_01464* | 1.50E-06 | 1.20E-03 | I-poly-cis-decaprenylcistransferase | 165 | Transferase |
| *FAVG1_12635* | 2.00E-06 | 1.50E-03 | Related to fibroin-3 | 398 | Structural protein |
| *FAVG1_01159* | 3.00E-06 | 1.90E-03 | Ras-2 GTPase | 48 | Pathogenicity factor FG / Signal transduction |
| *FAVG1_10120* | 3.00E-06 | 1.90E-03 | NADP-dependent L-serine/L-allo-threonine dehydrogenase YdfG | 42 | Oxidoreductase |
| *FAVG1_05271* | 3.00E-06 | 1.90E-03 | Domain-containing protein | 85 | Unknown |
| *FAVG1_12805* | 3.10E-06 | 1.90E-03 | C2h2 type zinc finger domain-containing protein | 86 | Regulator |
| *FAVG1_01832* | 4.50E-06 | 2.30E-03 | Endoplasmic reticulum-Golgi intermediate compartment protein3 | 129 | Transport |
| *FAVG1_04245* | 5.60E-06 | 2.30E-03 | Polyadenylate-binding protein | 475 | mRNA metabolism |
| *FAVG1_04808* | 5.80E-06 | 2.30E-03 | Extracellular dioxygenase | 116 | Oxidoreductase |
| *FAVG1_08154* | 6.40E-06 | 2.30E-03 | PAPA-1-like conserved region family protein | 79 | PAP-1 binding domain |
| *FAVG1_12373* | 6.70E-06 | 2.30E-03 | DNase1 protein | 413 | Endonuclease |
| *FAVG1_00245* | 6.70E-06 | 2.30E-03 | Vesicle-associated membrane protein | 46 | Vescicle fusion |
| *FAVG1_07281* | 6.70E-06 | 2.30E-03 | Zinc finger transcription factor ace1 | 17 | Regulator of cellobiohydrolase |
| *FAVG1_09092* | 6.70E-06 | 2.30E-03 | GATA-binding transcription factor | 65 | Regulator |
| *FAVG1_11769* | 6.70E-06 | 2.30E-03 | Ring-6 like protein | 36 | Multifunctional protein |
| *FAVG1_02888* | 6.70E-06 | 2.30E-03 | Histidine kinase m232p | 10 | Signal transcduction |
| *FAVG1_03635* | 6.70E-06 | 2.30E-03 | Protein kinase | 14 | Signal transcduction |
| *FAVG1_03965* | 6.70E-06 | 2.30E-03 | Acetyltransferase | 58 | DNA repair |
| *FAVG1_05942* | 6.70E-06 | 2.30E-03 | NRS13 (AMP-Reductase-TE) | 9 | NRPS (secondary metabolism) |
| *FAVG1_06700* | 7.20E-06 | 2.30E-03 | Cytochrome p450 | 18 | Oxidase |
| *FAVG1_08019* | 7.20E-06 | 2.30E-03 | Ran1-like protein kinase | 41 | Signal transcduction |
| *FAVG1_11964* | 7.20E-06 | 2.30E-03 | Nuclear and cytoplasmic polyadenylated RNA-binding protein PUB1 | 219 | mRNA-binding |
| *FAVG1_05239* | 7.20E-06 | 2.30E-03 | Hypothetical protein | 31 | Unknown |
| *FAVG1_05465* | 7.20E-06 | 2.30E-03 | Cellular morphogenesis protein | 33 | Regulator |
| *FAVG1_06002* | 7.20E-06 | 2.30E-03 | Aldose 1-epimerase | 51 | Carbohydrate metabolism |
| *FAVG1_06376* | 7.20E-06 | 2.30E-03 | Ring finger domain | 55 | Ligase |
| *FAVG1_03436* | 7.70E-06 | 2.40E-03 | Scp-like extracellular | 585 | Extracellular |
| *FAVG1_05235* | 8.00E-06 | 2.40E-03 | Uncharacterized conserved protein UCP014753 | 34 | Unknown |
| *FAVG1_09082* | 8.10E-06 | 2.40E-03 | Glycoside hydrolase Family 2 protein | 76 | Hydrolase |
| *FAVG1_06491* | 9.20E-06 | 2.60E-03 | Glycoside hydrolase Family 16 | 395 | Hydrolase |
| *FAVG1_01532* | 9.60E-06 | 2.70E-03 | Mitochondrial import inner membrane translocase subunit Tim-54 | 74 | Translocase |
| *FAVG1_00527* | 9.70E-06 | 2.70E-03 | Ron sulfur cluster assembly protein | 359 | Response to stress |
| *FAVG1_12417* | 1.20E-05 | 3.20E-03 | NAD dependent epimerase dehydratase family protein | 120 | Metabolism |
| *FAVG1_10761* | 1.30E-05 | 3.40E-03 | Extracellular serine-rich protein | 140 | Response to stress |
| *FAVG1_07505* | 1.40E-05 | 3.60E-03 | Cystathionine beta-synthase | 159 | Hydrolase |
| *FAVG1_04879* | 1.40E-05 | 3.60E-03 | UBX domain-containing protein | 128 | Response to stress |
| *FAVG1_00700* | 1.50E-05 | 3.60E-03 | Cation-transporting atpase 4 | 44 | Hydrolase |
| *FAVG1_03890* | 1.50E-05 | 3.60E-03 | T-complex protein 1 subunit alpha | 161 | Response to stress |
| *FAVG1_00003* | 1.70E-05 | 4.10E-03 | Guanine nucleotide-binding protein alpha subunit | 100 | Response to stress/Signal transduction |
| *FAVG1_05871* | 1.70E-05 | 4.10E-03 | Nucleosome assembly protein | 527 | Response to stress |
| *FAVG1_06537* | 1.90E-05 | 4.10E-03 | Ribosomal protein ymr-31 | 155 | Ribosome |
| *FAVG1_01613* | 1.90E-05 | 4.10E-03 | Solute carrier Family 35 member c2 | 34 | Oxidative stress |
| *FAVG1_12028* | 1.90E-05 | 4.10E-03 | PDT domain-containing protein | 70 | Biosynthesis |
| *FAVG1_03515* | 1.90E-05 | 4.10E-03 | AFG1-like protein | 39 | Oxidative stress |
| *FAVG1_04499* | 2.00E-05 | 4.40E-03 | Hypothetical protein FG08339.1 | 248 | Unknown |
| *FAVG1_02619* | 2.10E-05 | 4.40E-03 | carboxypeptidase 2 | 78 | Hydrolase |
| *FAVG1_07152* | 2.10E-05 | 4.40E-03 | Calcium proton exchanger | 51 | Transport |
| *FAVG1_01444* | 2.10E-05 | 4.40E-03 | Hypothetical protein FOXB_00208 | 63 | Unknown |
| *FAVG1_07438* | 2.20E-05 | 4.50E-03 | Beta-Tubulin 2 | 436 | Microtubules |
| *FAVG1_06286* | 2.30E-05 | 4.50E-03 | ATP-dependent permease MDL2 | 60 | Transport |
| *FAVG1_07871* | 2.30E-05 | 4.50E-03 | Flavin-containing monooxygenase | 275 | Oxidative stress |
| *FAVG1_05429* | 2.30E-05 | 4.50E-03 | RNP domain-containing protein | 363 | Regulation |
| *FAVG1_01144* | 2.50E-05 | 4.80E-03 | APSES transcription factor StuA | 81 | Pathogenicity factor [2] |
| *FAVG1_03946* | 2.70E-05 | 4.90E-03 | CoxI translation protein cya5 | 27 | Protein synthesis |
| *FAVG1_04047* | 2.70E-05 | 4.90E-03 | Phosphatidylethanolamine N-methyltransferase | 37 | Metabolism |
| *FAVG1_05085* | 2.80E-05 | 4.90E-03 | Periplasmic binding ii | 77 | Nitrogen starvation |
| *FAVG1_05673* | 2.80E-05 | 4.90E-03 | Vesicular integral-membrane protein VIP36 | 68 | Transport |
| *FAVG1_01453* | 2.80E-05 | 4.90E-03 | Shwachman-Bodian-Diamond syndrome protein | 2294 | RNA biosynthesis |
| *FAVG1_05634* | 2.80E-05 | 4.90E-03 | Transmembrane protein | 74 | Transport |
| *FAVG1_01656* | 2.90E-05 | 4.90E-03 | DNA-directed RNA polymerase ii largest subunit | 63 | Transcription |
| *FAVG1_00305* | 2.90E-05 | 4.90E-03 | Signal recognition particle | 356 | Transport |
| *FAVG1_01721* | 2.90E-05 | 4.90E-03 | Ribosomal recycling Factor | 109 | RNA biosynthesis |
| *FAVG1_08957* | 3.00E-05 | 4.90E-03 | Translation initiation Factor eIF4e | 233 | Response to stress |
| *FAVG1_03388* | 3.00E-05 | 4.90E-03 | Stromal membrane-associated protein | 89 | Oxidative stress |
| *FAVG1_03618* | 3.00E-05 | 4.90E-03 | Proteasome component y13 | 199 | Proteolysis |
| *FAVG1_02417* | 3.00E-05 | 4.90E-03 | Bet v1-like protein | 994 | Unknown |

^1^Mostly based on FSRD: fungal stress response database [3].

| ***Table S11. Putative pathogenicity factors found in the transcriptome on barley*** | | |
| --- | --- | --- |
| **Signal transduction** | | **References** |
| *FAVG1_01159* | *RAS2* GTPase | [4] |
| *FAVG1_03504* | *HOG1*, a MAPK involved in osmotic stress | [5] |
| *FAVG1_05419* | *MAP1*, a MAPK involved in mating and filamentation | [6] |
| *FAVG1_01307* | *MGV1*, a cell integrity MAPK | [6] |
| *FAVG1_00904* | *PKAR* , a protein kinase A | [6] |
| *FAVG1_00895* | *SNF1*, a sucrose non-fermenting protein | [7] |
| *FAVG1_12030* | *STE11*, MAPKKK, hypersensitiv to MsDEF1 | [6] |
| *FAVG1_00194* | *TEP1*, Tensin-like phosphatase 1 | [8] |
| *FAVG1_01132* | *ATF1,* Activating Transcription Factor | [9] |
| **Metabolism** | |  |
| *FAVG1_00587* | *ADE5*, Phosphoribosylamine-glycine ligase | [6] |
| *FAVG1_04963* | *GCS1*, Glycosylceramide synthase (sphingolipid biosynthesis) | [6] |
| *FAVG1_03066* | *HMR1*, 3-hydroxy-3-methylglutaryl-coenzyme A reductase | [6] |
| *FAVG1_07766* | *MT2*, Sphingolipid C-9-methyltransferase | [10] |
| *FAVG1_00893* | *NTH1*, Natural trehalase | [6] |
| *FAVG1_03623* | *SID1*, Siderophore biosynthetic gene | [6] |
| **Energy** | |  |
| *FAVG1_05042* | *ACL1*, ATP citrate lyase | [11] |
| **Interaction with the environment** | |  |
| *FAVG1_06380* | Similar to HET-C2 glycolipid transferase | [12] |
| **Cellular transport, transport facilities and transport routes** | |  |
| *FAVG1_00922* | *SYN2*, Snare protein (transport docking and vesicle fusion) | [13] |
| **Transcription and DNA modification** | |  |
| *FAVG1_01144* | *STUA*, APSES transcription factor | [2] |
| *FAVG1_12497* | *TOP1*, Topoisomerase 1 | [14] |
| **Cellular rescue, defence and virulence** | |  |
| *FAVG1_09966* | *PTC1*, Type 2C protein phosphatase | [15] |

| ***Table S12: Carbohydrate-Active enZYmes (CAZy)*** | | | | | |
| --- | --- | --- | --- | --- | --- |
| CAZy | FA | FG | FO | FV | FS |
| GT4 | 990 | 986 | 1304 | 1080 | 1199 |
| GT2 | 974 | 958 | 1272 | 1016 | 1143 |
| GH13 | 576 | 653 | 781 | 631 | 701 |
| GH18 | 272 | 254 | 374 | 278 | 323 |
| GH23 | 264 | 266 | 317 | 258 | 277 |
| GT1 | 237 | 241 | 308 | 238 | 250 |
| GH3 | 234 | 267 | 355 | 253 | 336 |
| GT51 | 222 | 228 | 269 | 235 | 248 |
| GH2 | 196 | 190 | 262 | 189 | 209 |
| GH28 | 194 | 155 | 217 | 167 | 274 |
| GT0 | 161 | 148 | 186 | 154 | 176 |
| GH16 | 137 | 147 | 155 | 126 | 134 |
| GH5 | 134 | 140 | 183 | 124 | 174 |
| CE10 | 130 | 110 | 193 | 158 | 190 |
| CE4 | 127 | 131 | 139 | 115 | 137 |
| CE0 | 122 | 91 | 144 | 116 | 146 |
| GH43 | 120 | 142 | 194 | 151 | 180 |
| GT55 | 115 | 82 | 105 | 79 | 103 |
| AA3 | 114 | 102 | 160 | 119 | 147 |
| GH20 | 113 | 94 | 143 | 129 | 114 |
| GT5 | 110 | 118 | 135 | 86 | 143 |
| GH0 | 108 | 93 | 155 | 113 | 140 |
| GH1 | 108 | 99 | 175 | 115 | 105 |
| GH92 | 103 | 84 | 131 | 109 | 122 |
| GT35 | 99 | 106 | 118 | 94 | 116 |
| GH31 | 98 | 100 | 121 | 118 | 143 |
| GT48 | 98 | 111 | 142 | 115 | 135 |
| CBM50 | 96 | 99 | 109 | 92 | 117 |
| CE1 | 93 | 78 | 105 | 88 | 87 |
| GH79 | 93 | 90 | 137 | 116 | 161 |
| GH36 | 88 | 97 | 111 | 91 | 94 |
| CBM13 | 85 | 83 | 98 | 95 | 99 |
| GH10 | 81 | 62 | 89 | 80 | 93 |
| GT41 | 79 | 95 | 124 | 83 | 102 |
| GT9 | 72 | 57 | 100 | 87 | 86 |
| GH73 | 68 | 57 | 89 | 68 | 76 |
| GT34 | 68 | 71 | 86 | 70 | 92 |
| AA9 | 64 | 51 | 61 | 46 | 69 |
| GH15 | 63 | 56 | 76 | 64 | 74 |
| GH47 | 63 | 72 | 78 | 74 | 79 |
| GT20 | 61 | 60 | 89 | 80 | 69 |
| GH32 | 61 | 86 | 135 | 80 | 75 |
| CBM20 | 59 | 52 | 62 | 48 | 60 |
| GH76 | 58 | 41 | 79 | 54 | 77 |
| GH57 | 57 | 58 | 79 | 63 | 72 |
| GT8 | 55 | 49 | 73 | 51 | 58 |
| GH78 | 55 | 50 | 72 | 60 | 72 |
| GH77 | 55 | 60 | 94 | 53 | 57 |
| GH33 | 54 | 42 | 59 | 58 | 47 |
| GH65 | 54 | 51 | 69 | 53 | 60 |
| GH17 | 53 | 54 | 79 | 56 | 70 |
| GH38 | 52 | 53 | 110 | 71 | 68 |
| GT84 | 48 | 64 | 80 | 66 | 68 |
| GT28 | 45 | 44 | 67 | 51 | 55 |
| CE9 | 45 | 53 | 72 | 53 | 68 |
| AA7 | 44 | 33 | 61 | 43 | 53 |
| CE11 | 44 | 50 | 54 | 46 | 55 |
| GH9 | 43 | 50 | 67 | 52 | 67 |
| GH35 | 42 | 34 | 64 | 43 | 54 |
| CBM32 | 39 | 27 | 47 | 41 | 35 |
| PL1 | 39 | 46 | 54 | 41 | 53 |
| AA1 | 39 | 51 | 63 | 49 | 53 |
| GH51 | 37 | 28 | 67 | 39 | 49 |
| GT83 | 37 | 36 | 48 | 42 | 41 |
| GT22 | 37 | 37 | 42 | 26 | 42 |
| GH4 | 36 | 37 | 46 | 43 | 40 |
| GH29 | 36 | 40 | 57 | 38 | 32 |
| GH25 | 35 | 39 | 36 | 33 | 26 |
| CBM2 | 33 | 24 | 50 | 34 | 37 |
| GT31 | 33 | 34 | 46 | 37 | 34 |
| GT30 | 32 | 27 | 40 | 37 | 41 |
| GH105 | 31 | 21 | 38 | 25 | 33 |
| CE8 | 31 | 25 | 40 | 35 | 41 |
| GH19 | 30 | 21 | 29 | 29 | 20 |
| GH55 | 30 | 25 | 38 | 29 | 32 |
| GH37 | 30 | 29 | 38 | 31 | 44 |
| GT15 | 29 | 26 | 27 | 27 | 32 |
| GH103 | 29 | 29 | 32 | 21 | 37 |
| GH95 | 29 | 36 | 50 | 36 | 30 |
| GT77 | 28 | 30 | 34 | 32 | 30 |
| GT66 | 28 | 34 | 39 | 30 | 38 |
| CBM12 | 27 | 20 | 24 | 21 | 25 |
| CBM57 | 27 | 27 | 45 | 27 | 23 |
| GT39 | 27 | 32 | 37 | 30 | 38 |
| CBM48 | 26 | 17 | 24 | 21 | 28 |
| GH42 | 26 | 22 | 34 | 25 | 36 |
| GT32 | 26 | 42 | 41 | 38 | 39 |
| GH71 | 25 | 21 | 33 | 24 | 36 |
| GH94 | 24 | 31 | 29 | 20 | 21 |
| GH81 | 23 | 15 | 20 | 21 | 15 |
| CBM5 | 23 | 17 | 19 | 18 | 24 |
| GT25 | 23 | 18 | 27 | 20 | 26 |
| GH27 | 23 | 28 | 43 | 26 | 26 |
| GH26 | 22 | 16 | 30 | 25 | 25 |
| GH93 | 22 | 16 | 29 | 19 | 19 |
| GH63 | 22 | 17 | 27 | 19 | 18 |
| GH115 | 22 | 19 | 25 | 27 | 11 |
| CE12 | 22 | 21 | 42 | 34 | 40 |
| GH39 | 22 | 22 | 29 | 25 | 24 |
| GH30 | 22 | 25 | 53 | 25 | 32 |
| AA2 | 21 | 21 | 23 | 14 | 15 |
| GT19 | 21 | 23 | 22 | 20 | 26 |
| CBM6 | 21 | 28 | 33 | 31 | 26 |
| GT44 | 20 | 15 | 31 | 13 | 14 |
| GH72 | 20 | 19 | 30 | 20 | 20 |
| GT26 | 20 | 22 | 26 | 26 | 19 |
| CBM14 | 20 | 23 | 33 | 25 | 30 |
| GT90 | 20 | 29 | 29 | 32 | 26 |
| GH102 | 19 | 19 | 24 | 24 | 16 |
| GT14 | 18 | 9 | 19 | 14 | 19 |
| GH53 | 18 | 19 | 16 | 18 | 18 |
| PL4 | 18 | 21 | 23 | 14 | 22 |
| GH84 | 17 | 15 | 19 | 18 | 23 |
| GH34 | 17 | 15 | 17 | 16 | 10 |
| AA10 | 17 | 15 | 13 | 15 | 14 |
| PL8 | 17 | 16 | 36 | 17 | 17 |
| GT7 | 17 | 17 | 14 | 17 | 16 |
| GH130 | 16 | 13 | 16 | 16 | 16 |
| CE5 | 16 | 14 | 18 | 19 | 14 |
| CE7 | 15 | 4 | 26 | 19 | 17 |
| GT62 | 15 | 8 | 16 | 10 | 13 |
| CE3 | 15 | 14 | 21 | 24 | 22 |
| GT71 | 15 | 15 | 23 | 17 | 21 |
| GH74 | 15 | 16 | 22 | 12 | 8 |
| GT53 | 14 | 9 | 11 | 8 | 13 |
| GH88 | 14 | 12 | 15 | 12 | 14 |
| GH70 | 14 | 14 | 30 | 24 | 21 |
| CBM18 | 14 | 15 | 14 | 12 | 12 |
| CBM51 | 14 | 16 | 15 | 12 | 13 |
| GH8 | 13 | 9 | 17 | 15 | 7 |
| GT29 | 13 | 11 | 23 | 15 | 17 |
| GH127 | 13 | 14 | 17 | 18 | 29 |
| PL9 | 13 | 14 | 24 | 18 | 16 |
| AA5 | 13 | 14 | 10 | 12 | 15 |
| CE14 | 13 | 25 | 26 | 23 | 21 |
| GH24 | 12 | 12 | 38 | 11 | 21 |
| GT10 | 12 | 12 | 15 | 12 | 10 |
| PL3 | 12 | 12 | 14 | 13 | 16 |
| CBM1 | 12 | 18 | 17 | 15 | 13 |
| GT57 | 11 | 4 | 8 | 5 | 6 |
| GH67 | 11 | 5 | 19 | 9 | 11 |
| GH109 | 11 | 9 | 14 | 17 | 21 |
| PL0 | 11 | 16 | 27 | 16 | 15 |
| GT27 | 11 | 16 | 12 | 8 | 11 |
| GH106 | 11 | 17 | 25 | 14 | 15 |
| CBM3 | 10 | 2 | 4 | 4 | 9 |
| GH12 | 10 | 10 | 11 | 9 | 19 |
| AA8 | 10 | 13 | 10 | 9 | 16 |
| GT24 | 10 | 14 | 13 | 18 | 19 |
| GT3 | 10 | 15 | 13 | 22 | 13 |
| GT47 | 10 | 18 | 29 | 10 | 13 |
| GH7 | 10 | 18 | 19 | 17 | 31 |
| CBM47 | 9 | 3 | 4 | 2 | 4 |
| GH75 | 9 | 6 | 9 | 10 | 11 |
| GH116 | 9 | 7 | 17 | 13 | 8 |
| GT87 | 9 | 13 | 17 | 10 | 11 |
| GT61 | 8 | 4 | 11 | 6 | 11 |
| PL12 | 8 | 4 | 10 | 5 | 9 |
| GT64 | 8 | 6 | 7 | 5 | 4 |
| GT58 | 8 | 6 | 8 | 8 | 5 |
| CBM9 | 8 | 7 | 14 | 8 | 6 |
| GH87 | 8 | 7 | 6 | 10 | 7 |
| GH11 | 8 | 8 | 15 | 10 | 12 |
| GT81 | 8 | 9 | 9 | 14 | 9 |
| GH85 | 8 | 10 | 20 | 10 | 10 |
| PL10 | 8 | 11 | 6 | 3 | 8 |
| GH97 | 8 | 12 | 17 | 20 | 20 |
| GH125 | 8 | 15 | 14 | 13 | 12 |
| GH114 | 7 | 4 | 6 | 5 | 7 |
| GH64 | 7 | 5 | 10 | 10 | 11 |
| GH48 | 7 | 5 | 9 | 3 | 6 |
| GH50 | 7 | 5 | 8 | 7 | 4 |
| AA4 | 7 | 6 | 9 | 10 | 15 |
| GH99 | 7 | 6 | 4 | 7 | 4 |
| CBM21 | 7 | 7 | 18 | 6 | 13 |
| GH62 | 7 | 7 | 4 | 4 | 7 |
| CE16 | 7 | 7 | 5 | 7 | 6 |
| GT11 | 7 | 8 | 12 | 6 | 7 |
| GH128 | 7 | 9 | 10 | 7 | 9 |
| GH6 | 7 | 12 | 9 | 14 | 14 |
| GH66 | 6 | 1 | 5 | 5 | 6 |
| GH108 | 6 | 2 | 5 | 2 | 5 |
| GT60 | 6 | 6 | 10 | 4 | 5 |
| GT43 | 6 | 6 | 7 | 9 | 7 |
| GH14 | 6 | 7 | 8 | 8 | 4 |
| GT21 | 6 | 9 | 9 | 11 | 16 |
| GT56 | 5 | 1 | 3 | 2 | 3 |
| PL22 | 5 | 2 | 6 | 2 | 6 |
| GH104 | 5 | 3 | 2 | 6 | 4 |
| GT13 | 5 | 3 | 6 | 3 |  |
| GT69 | 5 | 4 | 5 | 8 | 9 |
| CE15 | 5 | 5 | 6 | 6 | 10 |
| AA6 | 5 | 5 | 2 | 7 | 6 |
| CBM63 | 5 | 5 | 7 | 5 | 5 |
| GT50 | 5 | 8 | 8 | 6 | 8 |
| GH132 | 5 | 9 | 11 | 13 | 12 |
| PL11 | 5 | 11 | 11 | 14 | 20 |
| GH56 | 5 | 11 | 2 | 4 | 2 |
| GT65 | 4 |  | 2 | 1 | 3 |
| GH46 | 4 | 1 | 3 | 6 | 1 |
| GH98 | 4 | 1 |  | 2 | 1 |
| GT54 | 4 | 1 | 2 | 2 | 2 |
| GT92 | 4 | 2 | 9 | 7 | 4 |
| PL6 | 4 | 2 | 2 | 5 | 4 |
| GH120 | 4 | 3 | 1 | 3 | 9 |
| GT23 | 4 | 3 | 4 | 2 | 5 |
| GT76 | 4 | 4 | 3 | 4 | 3 |
| GH112 | 4 | 5 | 6 | 1 | 1 |
| PL7 | 4 | 5 | 8 | 2 | 5 |
| CBM0 | 4 | 6 | 11 | 8 | 7 |
| CE6 | 4 | 6 | 9 | 6 | 10 |
| GT33 | 4 | 7 | 8 | 8 | 10 |
| GH44 | 4 | 7 | 8 | 4 | 5 |
| GH68 | 4 | 8 | 15 | 4 | 5 |
| GH83 | 4 | 8 | 8 | 5 | 6 |
| GT49 | 4 | 9 | 4 | 5 | 4 |
| CE2 | 4 | 10 | 11 | 6 | 7 |
| GH121 | 3 | 1 | 5 | 3 | 1 |
| GH59 | 3 | 1 | 3 | 4 | 4 |
| GH91 | 3 | 1 | 3 |  |  |
| GH131 | 3 | 1 | 2 | 1 | 2 |
| CBM54 | 3 | 2 | 8 | 6 | 2 |
| CBM16 | 3 | 2 | 7 | 6 | 5 |
| CBM35 | 3 | 2 | 4 | 2 | 4 |
| CBM43 | 3 | 2 | 3 | 1 | 3 |
| GH45 | 3 | 2 | 3 | 1 | 2 |
| GT17 | 3 | 3 | 3 | 3 | 6 |
| GH86 | 3 | 4 | 8 | 4 | 4 |
| GT89 | 3 | 5 | 2 | 3 | 2 |
| GH54 | 3 | 5 | 3 | 4 | 2 |
| GH101 | 3 | 6 | 12 | 6 | 3 |
| PL5 | 2 |  | 4 | 1 |  |
| GH117 | 2 |  | 3 | 3 | 1 |
| GT40 | 2 |  |  |  | 1 |
| GT82 | 2 |  |  | 1 |  |
| CBM42 | 2 |  |  | 1 | 2 |
| CBM4 | 2 |  | 2 | 3 | 2 |
| CBM45 | 2 | 1 | 5 | 1 | 1 |
| PL17 | 2 | 1 | 4 | 5 | 4 |
| GH52 | 2 | 1 | 3 | 3 | 2 |
| GT88 | 2 | 1 | 2 |  | 1 |
| GT75 | 2 | 1 | 2 | 1 | 1 |
| GH126 | 2 | 1 | 1 | 1 |  |
| GT37 | 2 | 2 | 6 | 5 | 8 |
| GT67 | 2 | 2 | 7 | 5 | 2 |
| GH113 | 2 | 2 | 3 | 3 | 3 |
| GH49 | 2 | 2 | 2 | 1 | 1 |
| CBM26 | 2 | 2 | 2 | 2 | 3 |
| CBM37 | 2 | 3 | 5 | 6 | 1 |
| GH22 | 2 | 3 | 2 | 2 | 2 |
| GT91 | 2 | 4 | 3 | 4 | 6 |
| GH100 | 2 | 5 | 12 | 7 | 9 |
| GH89 | 2 | 7 | 17 | 11 | 12 |
| GT38 | 1 |  |  |  |  |
| PL15 | 1 |  | 2 | 2 | 1 |
| GH58 | 1 |  | 1 | 2 | 1 |
| GH119 | 1 |  | 1 |  |  |
| CBM27 | 1 |  | 1 |  |  |
| PL2 | 1 | 1 | 5 |  | 1 |
| GH107 | 1 | 1 | 1 | 1 | 2 |
| PL20 | 1 | 1 | 1 | 1 | 2 |
| GT42 | 1 | 1 | 2 | 1 | 1 |
| GT80 | 1 | 1 |  | 1 |  |
| GT74 | 1 | 1 |  |  |  |
| GT18 | 1 | 1 | 1 | 1 | 1 |
| CBM22 | 1 | 1 |  | 1 | 1 |
| CBM38 | 1 | 2 | 4 | 1 |  |
| GT68 | 1 | 2 | 3 | 3 | 1 |
| CBM67 | 1 | 2 | 3 | 2 | 1 |
| GT70 | 1 | 2 |  | 2 |  |
| GH122 | 1 | 2 | 2 | 2 | 1 |
| PL21 | 1 | 2 | 1 | 1 |  |
| CBM52 | 1 | 2 | 1 | 1 | 1 |
| GH110 | 1 | 4 | 4 | 4 | 3 |
| GT59 | 1 | 4 | 3 | 3 | 3 |

**References**

1. Jin JM, Lee S, Lee J, Baek SR, Kim JC, Yun SH, Park SY, Kang SC, Lee YW (2010) Functional characterization and manipulation of the apicidin biosynthetic pathway in *Fusarium semitectum*. Mol Microbiol 76: 456-466.

2. Lysøe E, Pasquali M, Breakspear A, Kistler HC (2011) The transcription factor FgStuAp influences spore development, pathogenicity, and secondary metabolism in *Fusarium graminearum*. Mol Plant Microbe Interact 24: 54-67.

3. Karányi Z, Holb I, Hornok L, Pócsi I, Miskei M (2013) FSRD: fungal stress response database. Database (Oxford) 2013: bat037.

4. Bluhm BH, Zhao X, Flaherty JE, Xu JR, Dunkle LD (2007) RAS2 regulates growth and pathogenesis in *Fusarium graminearum*. Mol Plant Microbe Interact 20: 627-636.

5. Ochiai N, Tokai T, Nishiuchi T, Takahashi-Ando N, Fujimura M, Kimura M (2007) Involvement of the osmosensor histidine kinase and osmotic stress-activated protein kinases in the regulation of secondary metabolism in *Fusarium graminearum*. Biochem Biophys Res Commun 363: 639-644.

6. Urban M, Hammond-Kosack KE (2013) Molecular genetics and genomic approaches to explore *Fusarium* infection of wheat floral tissue. In: Brown DW, Proctor RH, editors. *Fusarium*: Genomics, Molecular and Cellular Biology. Norfolk, UK: Caister Academic Press. pp. 43-79.

7. Lee SH, Lee J, Lee S, Park EH, Kim KW, Kim MD, Yun SH, Lee YW (2009) GzSNF1 is required for normal sexual and asexual development in the ascomycete *Gibberella zeae*. Eukaryot Cell 8: 116-127.

8. Zhang DJ, Fan FY, Yang JR, Wang XL, Qiu DW, Jiang LH (2010) FgTep1p is linked to the phosphatidylinositol-3 kinase signalling pathway and plays a role in the virulence of *Fusarium graminearum* on wheat. Mol Plant Pathol 11: 495-502.

9. Nguyen TV, Kröger C, Bönnighausen J, Schäfer W, Bormann J (2013) The ATF/CREB transcription factor Atf1 is essential for full virulence, deoxynivalenol production and stress tolerance in the cereal pathogen *Fusarium graminearum*. Mol Plant Microbe Interact . doi: 10.1094/MPMI-04-13-0125-R.

10. Ramamoorthy V, Cahoon EB, Thokala M, Kaur J, Li J, Shah DM (2009) Sphingolipid C-9 methyltransferases are important for growth and virulence but not for sensitivity to antifungal plant defensins in *Fusarium graminearum*. Eukaryot Cell 8: 217-229.

11. Son H, Lee J, Park AR, Lee YW (2011) ATP citrate lyase is required for normal sexual and asexual development in *Gibberella zeae*. Fung Genet Biol 48: 408-417.

12. Dufresne M, van der Lee T, Ben M'Barek S, Xu XD, Zhang X, Liu TG, Waalwijk C, Zhang W, Kema GHJ, Daboussi MJ (2008) Transposon-tagging identifies novel pathogenicity genes in *Fusarium graminearum*. Fung Genet Biol 45: 1552-1561.

13. Hong SY, So J, Lee J, Min K, Son H, Park C, Yun SH, Lee YW (2010) Functional analyses of two syntaxin-like SNARE genes, GzSYN1 and GzSYN2, in the ascomycete *Gibberella zeae*. Fung Genet Biol 47: 364-372.

14. Baldwin TK, Urban M, Brown N, Hammond-Kosack KE (2010) A role for topoisomerase 1 in *Fusarium graminearum* and *F. culmorum* pathogenesis and sporulation. Mol Plant Microbe Interact 23: 566-577.

15. Jiang LH, Yang JR, Fan FY, Zhang DJ, Wang XL (2010) The Type 2C protein phosphatase FgPtc1p of the plant fungal pathogen *Fusarium graminearum* is involved in lithium toxicity and virulence. Mol Plant Pathol 11: 277-282.
